# A conserved CAF40-binding motif in metazoan NOT4 mediates association with the CCR4–NOT complex

**DOI:** 10.1101/gad.320952.118

**Published:** 2019-02-01

**Authors:** Csilla Keskeny, Tobias Raisch, Annamaria Sgromo, Cátia Igreja, Dipankar Bhandari, Oliver Weichenrieder, Elisa Izaurralde

**Affiliations:** Department of Biochemistry, Max Planck Institute for Developmental Biology, D-72076 Tübingen, Germany

**Keywords:** deadenylation, mRNA decay, translational repression, ubiquitination

## Abstract

The multisubunit CCR4–NOT mRNA deadenylase complex plays important roles in the posttranscriptional regulation of gene expression. The NOT4 E3 ubiquitin ligase is a stable component of the CCR4–NOT complex in yeast but does not copurify with the human or *Drosophila melanogaster* complex. Here we show that the C-terminal regions of human and *D. melanogaster* NOT4 contain a conserved sequence motif that directly binds the CAF40 subunit of the CCR4–NOT complex (CAF40-binding motif [CBM]). In addition, nonconserved sequences flanking the CBM also contact other subunits of the complex. Crystal structures of the CBM–CAF40 complex reveal a mutually exclusive binding surface for NOT4 and Roquin or Bag of marbles mRNA regulatory proteins. Furthermore, CAF40 depletion or structure-guided mutagenesis to disrupt the NOT4–CAF40 interaction impairs the ability of NOT4 to elicit decay of tethered reporter mRNAs in cells. Together with additional sequence analyses, our results reveal the molecular basis for the association of metazoan NOT4 with the CCR4–NOT complex and show that it deviates substantially from yeast. They mark the NOT4 ubiquitin ligase as an ancient but nonconstitutive cofactor of the CCR4–NOT deadenylase with potential recruitment and/or effector functions.

## Abstract (precis)

Keskeny et al. show that the C-terminal regions of human and *Drosophila melanogaster* NOT4 contain a conserved sequence motif that directly binds the CAF40 subunit of the CCR4–NOT complex.

The CCR4–NOT complex plays a central role in the posttranscriptional regulation of gene expression by catalyzing the removal of the mRNA poly(A) tail, thereby repressing translation and promoting mRNA degradation (Wahle and Winkler 2013; Collart 2016). In addition, the CCR4–NOT complex has the ability to repress translation independently of deadenylation (Cooke et al. 2010; Chekulaeva et al. 2011; Bawankar et al. 2013).

The CCR4–NOT complex (Fig. 1A) is a multisubunit complex (Chen et al. 2001; Lau et al. 2009; Temme et al. 2010) that assembles on the NOT1 scaffold protein, which consists of several α-helical domains that serve to dock the other subunits of the complex (Bawankar et al. 2013). Deadenylation is carried out by two interacting deadenylases; namely, CCR4 and CAF1. They dock onto a central α-helical domain in NOT1 (labeled “MIF4G”), forming the “catalytic module” of the complex (Basquin et al. 2012; Petit et al. 2012). The C-terminal end of NOT1 contains the NOT1 superfamily homology domain (SHD), which is another α-helical domain that interacts with the NOT2–NOT3 heterodimer to form the “NOT module” of the complex (Bhaskar et al. 2013; Boland et al. 2013). The catalytic module and the NOT module are connected by the CAF40-binding domain of NOT1 (labeled “CN9BD”) and a connector domain (labeled “MIF4G-C”) of unknown function (Chen et al. 2014; Mathys et al. 2014; Raisch et al. 2018). Both the NOT module and the CAF40 subunit of the CCR4–NOT complex have been reported as important peptide-docking sites for the recruitment of the complex by mRNA-associated proteins. The NOT module provides binding sites for Bicaudal-C (Chicoine et al. 2007), Nanos (Bhandari et al. 2014; Raisch et al. 2016), and Roquin (Sgromo et al. 2017). CAF40 is known to be contacted by Roquin (Sgromo et al. 2017), Bag of marbles (Bam) (Sgromo et al. 2018), and TTP (Bulbrook et al. 2018) as well as the GW182/TNRC6 family of proteins that mediates microRNA-mediated mRNA repression and decay (Chen et al. 2014; Mathys et al. 2014). The N-terminal portion of NOT1 is less well conserved than its C-terminal portion (Basquin et al. 2012) and serves to dock NOT10 and NOT11 as additional subunits of the complex in metazoan species (Bawankar et al. 2013; Mauxion et al. 2013). The CCR4–NOT complexes of *Saccharomyces cerevisiae* (*Sc*) and *Schizosaccharomyces pombe* lack NOT10 and NOT11 proteins. Furthermore, these CCR4–NOT complexes are special because they contain NOT4 as an integral component (Bai et al. 1999; Chen et al. 2001; Nasertorabi et al. 2011; Stowell et al. 2016; Ukleja et al. 2016).

**Figure 1.**
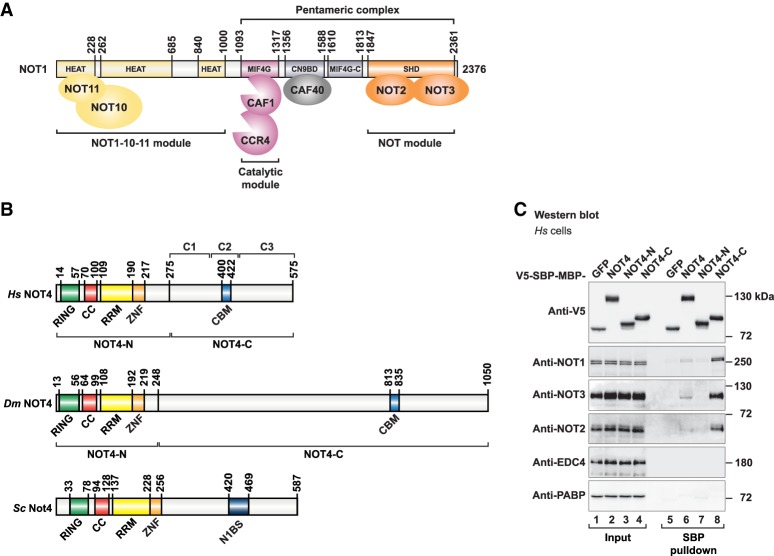
Human NOT4 interacts with the CCR4–NOT complex. (*A*) Composition of the human CCR4–NOT complex. The NOT1 scaffold protein contains N-terminal α-helical domains (classified as HEAT repeat domains) that interact with NOT10 and NOT11 to form the NOT1–10–11 module. NOT1 furthermore contains a central HEAT repeat domain (MIF4G) that binds CAF1 and CCR4 to form the catalytic module, an α-helical bundle that interacts with CAF40 (CN9BD), a connector domain (MIF4G-C), and a NOT1 SHD that forms the NOT module together with NOT2 and NOT3. A “pentameric” complex lacking CCR4 and the NOT1–10–11 module can be assembled from recombinant human CCR4–NOT proteins (Sgromo et al. 2017). (*B*) Domain composition of NOT4 proteins. The conserved N-terminal region of NOT4 (NOT4-N) comprises a RING-type E3 ubiquitin ligase domain (RING), a positively charged linker with coiled-coil propensity (CC), an RNA recognition motif (RRM) domain, and a C3H1-type zinc finger domain (ZNF). The nonconserved C-terminal region of NOT4 (NOT4-C) was found to interact with the CCR4–NOT complex. To map the interactions, *Homo sapiens* (*Hs*) NOT4-C was subdivided into three regions: C1 (residues P275–S376), C2 (residues E377–Q428), and C3 (residues P429–A575). A CAF40-binding motif (CBM) was identified in the C2 region. The CBM is conserved in metazoan NOT4, including *Dm* NOT4, but is not conserved in yeasts. Instead, *Sc* Not4-C harbors a previously characterized binding site for *Sc* Not1 (N1BS) (Bhaskar et al. 2015). (*C*) SBP pull-down of endogenous human NOT proteins with V5-SBP-MBP (V5-streptavidin-binding peptide-maltose-binding protein)-tagged *Hs* NOT4 from HEK293T cell lysates. V5-SBP-GFP-MBP served as negative control. Input samples correspond to 3% of the total lysate for V5-tagged proteins and 2% of the total lysate for NOT proteins. Pull-down samples correspond to 3% of the total pull-down for V5-tagged proteins and 35% of the total pull-down for NOT proteins. The mRNA decapping factor EDC4 and the poly(A)-binding protein PABP served as negative controls.

NOT4 (Fig. 1B) is an evolutionarily conserved E3 ubiquitin ligase that contains a RING domain, a linker region with coiled-coil propensity (CC), an RNA recognition motif (RRM) domain, and a C3H1-type zinc finger domain (ZNF). Together, they define the conserved N-terminal region of NOT4 (NOT4-N) (Fig. 1B). The C-terminal region of NOT4 (NOT4-C) (Fig. 1B) is predicted to be unstructured, and its sequence and length are not conserved among NOT4 proteins (The UniProt Consortium 2018). NOT4 causes the ubiquitination of diverse proteins in yeast and also human cells, targeting them for proteasomal degradation or resulting in regulatory changes. Ubiquitination targets include the nascent polypeptide-associated complex NAC (Panasenko et al. 2006), the histone demethylase JHD2 (Mersman et al. 2009), the transcription factor YAP1 (Gulshan et al. 2012), the master regulator of meiosis Mei2 (Simonetti et al. 2017), the cyclin C subunit of the Mediator complex (Cooper et al. 2012), the small ribosomal protein RPS7A (Panasenko and Collart 2012), and the cotranslational quality control factor ABCE1 (Wu et al. 2018). NOT4 has been implicated in cotranslational mRNA quality control and translational repression in the context of stalled ribosomes, such as in the “No-Go” mRNA decay pathway (Dimitrova et al. 2009; Matsuda et al. 2014; Panasenko 2014; Preissler et al. 2015; Wu et al. 2018).

A crystal structure demonstrated how *Sc* Not4 interacts with the SHD of *Sc* Not1 via an elongated polypeptide from the C-terminal region of *Sc* Not4 (Bhaskar et al. 2015). Using yeast two-hybrid assays, the human NOT4 and NOT1 proteins (*Homo sapiens* [*Hs*] NOT4 and *Hs* NOT1) were also shown to interact via the C-terminal portion of *Hs* NOT1 (Albert et al. 2002). However, the *Sc* Not1-binding sequence of *Sc* Not4 is only partially conserved, at best (Bhaskar et al. 2015), and NOT4 was not detected in mass spectrometric analyses of the native human and *Drosophila melanogaster* (*Dm*) CCR4–NOT complexes (Lau et al. 2009; Temme et al. 2010). This raised the question of whether NOT4 should be regarded as a component or cofactor of the CCR4–NOT complex in metazoans and how it would be recruited to the complex in species other than *S. cerevisiae*.

We therefore performed pull-down experiments from human cell extracts and with purified bacterially expressed proteins to identify and map interactions of human NOT4 with the CCR4–NOT complex. Assisted by alignments of metazoan NOT4 proteins, we uncovered a 23-amino-acid peptide motif in NOT4-C that binds to the CAF40 subunit of the CCR4–NOT complex and hence was termed the NOT4 CAF40-binding motif (CBM). Crystal structures of the CBM–CAF40 complex identified critical contacts required in human and *Dm* S2 cells for an efficient recruitment of NOT4 to the CCR4–NOT complex and for NOT4-mediated mRNA deadenylation and decay via the CCR4–NOT complex. Consequently, NOT4 emerges as a nonconstitutive cofactor of the CCR4–NOT complex in metazoans with a conserved mode of interaction via the CAF40 subunit.

## Results

### *Hs* NOT4-C shows a stable interaction with the CCR4–NOT complex

To investigate whether and how *Hs* NOT4 interacts with the CCR4–NOT complex in HEK293T cells, we expressed *Hs* NOT4 with a V5-SBP-MBP (V5-streptavidin-binding peptide-maltose-binding protein) tag in HEK293T cells and performed SBP pull-down assays. In agreement with previous reports (Lau et al. 2009; Temme et al. 2010), full-length *Hs* NOT4 failed to pull down the endogenous CCR4–NOT complex efficiently (Fig. 1C, lane 6). *Hs* NOT4-C, however, showed a stable interaction with the CCR4–NOT complex, as indicated by the detection of endogenous NOT1, NOT2, and NOT3 subunits in the pull-down fraction (Fig. 1C, lane 8). This is consistent with previous yeast two-hybrid experiments (Albert et al. 2002). In contrast, *Hs* NOT4-N did not interact with the CCR4–NOT complex (Fig. 1C, lane 7). The lack of an efficient interaction with the full-length protein remains unexplained but hints at a possible regulation of NOT4-C binding by NOT4-N. Additional SBP pull-down experiments showed that it is the presence of the positively charged CC linker and of the RRM domain in NOT4-N that prevents NOT4-C from interacting with the CCR4–NOT complex (Supplemental Fig. S1A,B).

### Tethered *Hs* NOT4 causes deadenylation-dependent mRNA decay

To address the relevance of an interaction between NOT4 and the CCR4–NOT complex with a functional assay, we investigated the molecular consequences resulting from the presence of NOT4 in the context of an mRNA. Usually, the recruitment of the CCR4–NOT complex to an mRNA target promotes its deadenylation-dependent decay (Wahle and Winkler 2013). It is unknown, however, whether NOT4 can bind to an mRNA despite the presence of putative and conserved RNA-binding domains in NOT4-N (CC-RRM-ZNF) (Fig. 1B). In the absence of known mRNA targets, we therefore used a tethering assay to direct NOT4 toward the 3′ untranslated region (UTR) of defined reporter mRNAs and tested them for NOT4-dependent deadenylation and decay.

**Figure 2.**
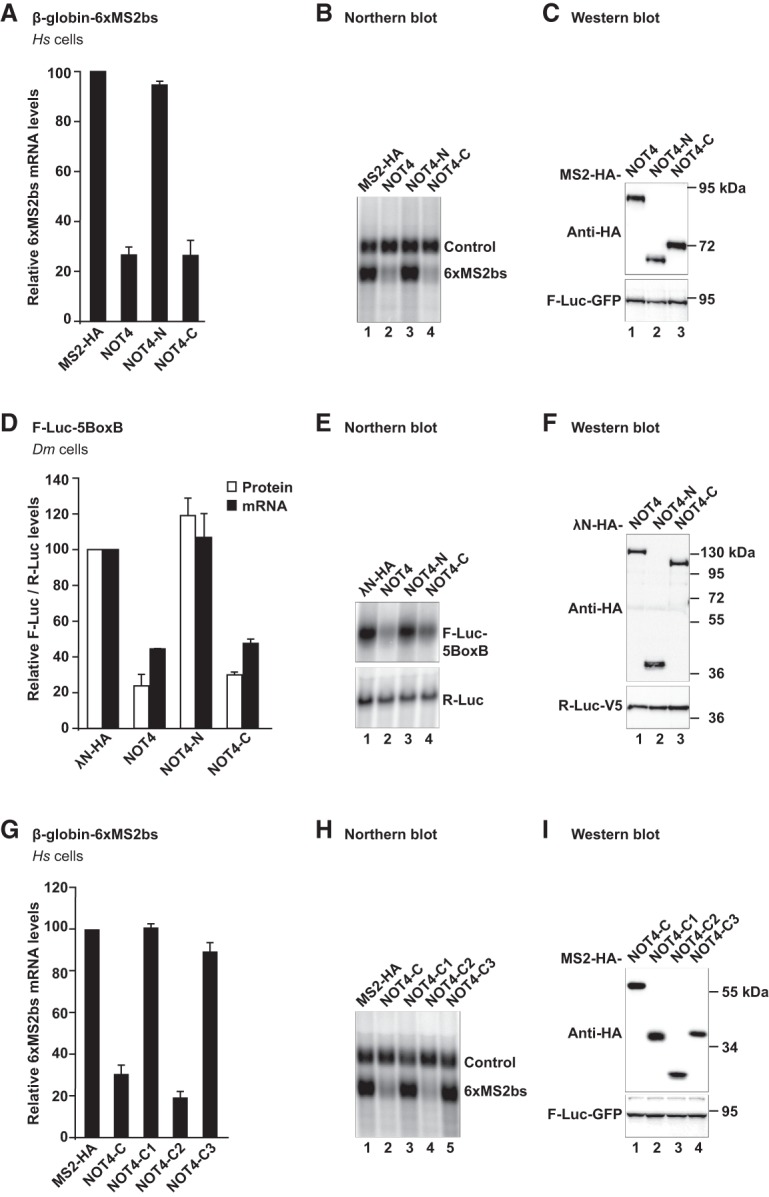
Metazoan NOT4 induces degradation of tethered mRNA reporters. (*A*–*C*) Tethering assay with *Hs* NOT4 and a β-globin mRNA reporter in HEK293T cells. *Hs* NOT4 or its fragments carried an N-terminal MS2-HA tag. β-Globin mRNA served as a reporter and contained six binding sites for the MS2 protein (6xMS2bs). β-Globin-GAPDH mRNA served as a reference and transfection control (control). (*A*) mRNA levels of the β-globin-6xMS2bs mRNA reporter normalized to the reference and plotted with respect to the values obtained from the expression of MS2-HA alone (set to 100). Error bars correspond to standard deviations. *n* = 3. (*B*) Northern blot of representative RNA samples. (*C*) Western blot demonstrating equal expression of MS2-HA-tagged proteins with F-Luc-GFP as a transfection control. (*D*–*F*) Tethering assay with *Dm* NOT4 and a luciferase reporter in *Dm* S2 cells. *Dm* NOT4 or its fragments carried an N-terminal λN-HA tag. Firefly luciferase mRNA served as a reporter and contained five BoxB binding sites for the λN peptide (F-Luc-5BoxB). Renilla luciferase mRNA served as a reference and transfection control (R-Luc). (*D*) F-Luc activity (white bars) and mRNA levels (black bars) normalized to the reference and plotted with respect to the values obtained from the expression of λN-HA alone (set to 100). Error bars correspond to standard deviations. *n* = 3. (*E*) Northern blot. (*F*) Western blot with R-Luc-V5 as a transfection control. (*G*–*I*) Tethering assay with fragments of *Hs* NOT4-C and the β-globin mRNA reporter. (*G*) Relative mRNA levels, with error bars corresponding to standard deviations. *n* = 3. For additional details, see *A*. (*H*) Northern blot. (*I*) Western blot.

In a first series of experiments, we tethered MS2-tagged *Hs* NOT4 to a β-globin mRNA reporter containing six MS2-binding sites in the 3′ UTR (β-globin-6xMS2bs) (Lykke-Andersen et al. 2000). Even though full-length *Hs* NOT4 did not associate with the CCR4–NOT complex in SBP pull-down assays (Fig. 1C, lane 6), we found that tethered MS2-HA-*Hs* NOT4 caused a substantial reduction of the β-globin-6xMS2bs mRNA level compared with the negative control MS2-HA (Fig. 2A,B). Tethering *Hs* NOT4-C also reduced mRNA levels, whereas tethering *Hs* NOT4-N had no effect (Fig. 2A,B). All *Hs* NOT4 fragments were expressed at comparable levels (Fig. 2C), and none of them affected the expression of the control β-globin mRNA lacking MS2-binding sites (Fig. 2B). Regarding full-length *Hs* NOT4, the discrepancy with the SBP pull-down assay (Fig. 1C) might be rationalized by a higher sensitivity of the tethering assay for weak and possibly transient interactions or, alternatively, by conformational changes of *Hs* NOT4 in the presence of RNA that promote the availability of NOT4-C to the deadenylase complex.

In a second series of experiments, we also tethered MS2-tagged *Hs* NOT4 to another reporter mRNA that encoded Renilla luciferase (R-Luc-6xMS2bs) instead of β-globin, allowing for the quantification of protein abundance in addition to mRNA levels. In agreement with the β-globin mRNA reporter, we observed a clear reduction of both Renilla luciferase mRNA and Renilla luciferase protein levels in the case of tethered *Hs* NOT4 or *Hs* NOT4-C (Supplemental Fig. S2A,B). By comparison, a Renilla luciferase mRNA lacking MS2-binding sites was not affected by the expression of MS2-tagged full-length *Hs* NOT4 or its fragments (Supplemental Fig. S2C,D).

To verify whether the reduction of reporter mRNA levels upon tethering *Hs* NOT4 or *Hs* NOT4-C resulted from deadenylation-dependent decay, we overexpressed a GFP-tagged catalytically inactive mutant of the human mRNA decapping enzyme DCP2 (GFP-DCP2 mut; E148Q). The presence of this mutant is known to impair mRNA decapping in a dominant-negative manner and hence 5′-to-3′ mRNA decay by XRN1 (Loh et al. 2013; Bhandari et al. 2014; Chang et al. 2014; Kuzuoğlu-Öztürk et al. 2016; Sgromo et al. 2017). Indeed, we observed the accumulation of a shorter deadenylated decay intermediate of the β-globin-6xMS2bs reporter mRNA upon tethering *Hs* NOT4 or upon tethering the *Hs* Nanos2 mRNA-binding protein (Bhandari et al. 2014), which served as a positive control (Supplemental Fig. S3A–C). We therefore attributed reporter mRNA decay to the recruitment of the CCR4–NOT complex, although we could not formally exclude contributions from other deadenylases at this stage.

### The capacity of NOT4-C to mediate tethered mRNA decay is conserved in metazoans

The C-terminal region of NOT4 is not conserved in sequence and length (Fig. 1B). We therefore investigated the functionality of *Dm* NOT4 as an example from an invertebrate species and to allow more general conclusions on NOT4 recruitment to the CCR4–NOT complex in metazoans. For *Dm* NOT4, we used a λN-based tethering assay in *Dm* S2 cells with an F-Luc-5BoxB reporter mRNA (Behm-Ansmant et al. 2006). Similar to *Hs* NOT4 and despite highly divergent sequences of NOT4-C, tethered *Dm* NOT4 and *Dm* NOT4-C efficiently mediated reporter mRNA decay (Fig. 2D,E). *Dm* NOT4 and its fragments were expressed at equal levels (Fig. 2F), and none of them affected the expression of an F-Luc reporter lacking the BoxB sites (Supplemental Fig. S2E,F).

Again, reporter mRNA decay was deadenylation-dependent. Deadenylated F-Luc-5BoxB reporter mRNA was stabilized in the presence of tethered *Dm* NOT4, when a GFP-tagged *Dm* DCP1 mutant (GFP-DCP1 mut; R70G, L71S, N72S, and T73G) known to prevent mRNA decapping in a dominant-negative manner (Chang et al. 2014; Kuzuoğlu-Öztürk et al. 2016) was overexpressed (Supplemental Fig. S3D–F). Tethered GW182 protein was used as a positive control (Kuzuoğlu-Öztürk et al. 2016). Together, our results indicate that the interaction of NOT4-C with the CCR4–NOT complex is conserved between humans and flies.

### *Hs* NOT4 directly interacts with the NOT1 and CAF40 subunits of the CCR4–NOT complex

**Figure 3.**
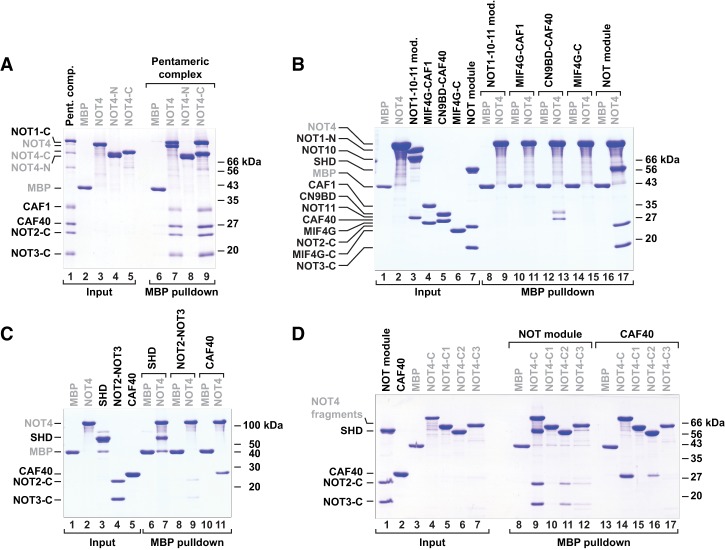
*Hs* NOT4 directly interacts with the NOT1-SHD and CAF40. (*A*–*D*) MBP pull-down assays with MBP-tagged *Hs* NOT4 and purified components of the human CCR4–NOT complex. MBP-tagged NOT4 or NOT4 fragments were used to pull down potential interaction partners. MBP alone served as a control. The respective starting materials (“input”) and pull-down samples were analyzed by SDS-PAGE and Coomassie blue staining. Potential interaction partners included a pentameric assembly of recombinant human CCR4–NOT proteins (*A*), various subassemblies of the CCR4–NOT proteins (*B*), and individual proteins (*C*). (*D*) To further confine individual interactions, *Hs* NOT4-C was subdivided into the C1, C2, and C3 regions. See Figure 1B for details. MBP-tagged constructs are labeled in gray.

To test whether the interaction between *Hs* NOT4 and the CCR4–NOT complex is direct, we expressed MBP-tagged *Hs* NOT4, *Hs* NOT4-N, and *Hs* NOT4-C in *Escherichia coli* and performed pull-down experiments with a reconstituted and purified subcomplex of human CCR4–NOT components that we had described previously (Sgromo et al. 2017) and that we here call the “pentameric” complex (Figs. 1A, 3A). This subcomplex comprises the C-terminal portion of NOT1 together with the CAF1 and CAF40 subunits and the C-terminal fragments of NOT2 and NOT3. Indeed, we observed a direct interaction with the pentameric complex. In contrast to the result from the SBP pull-down experiment in HEK293T cells (Fig. 1C, lane 6), the interaction occurred even with the full-length *Hs* NOT4 (Fig. 3A, lane 7). Furthermore, *Hs* NOT4-C interacted with the pentameric complex as efficiently as the recombinant full-length protein (Fig. 3A, lane 9), whereas recombinant *Hs* NOT4-N did not interact (Fig. 3A, lane 8).

To map the interactions to individual components of the CCR4–NOT complex, we also used previously described smaller subassemblies of the complex (Petit et al. 2012; Boland et al. 2013; Chen et al. 2014; Sgromo et al. 2017) in MBP pull-down experiments (Figs. 1A, 3B). These were the NOT1–NOT10–NOT11 module (comprising the N-terminal region of NOT1 and NOT10 and the C-terminal half of NOT11), CAF1 bound to the central α-helical domain in NOT1 (labeled “MIF4G”), CAF40 bound to the CAF40-binding domain of NOT1 (labeled “CN9BD”), the NOT1 connector domain (labeled “MIF4G-C”), and the NOT module (comprising the C-terminal regions of NOT1, NOT2, and NOT3). We detected interactions of *Hs* NOT4 with both the CAF40-containing subcomplex and the NOT module (Fig. 3B, lanes 13,17), pointing to multiple NOT4-binding sites within the CCR4–NOT complex. Finally, we narrowed down these interactions even further, primarily to the NOT1-SHD (Fig. 3C, lane 7) and CAF40 alone (Fig. 3C, lane 11), with only minor contributions from the NOT2–NOT3 heterodimer (Fig. 3C, lane 9).

### The central C2 region of *Hs* NOT4-C is sufficient to contact CCR4–NOT and trigger decay of tethered mRNA

It is not uncommon for the long unstructured portions of regulatory mRNA-associated proteins (such as in GW182/TNRC6, Nanos, or Roquin) to show multiple interactions with the CCR4–NOT complex, targeting several of its subunits (Jonas and Izaurralde 2015; Raisch et al. 2016; Sgromo et al. 2017). In order to map the interactions with the NOT module and CAF40 more precisely, we subdivided *Hs* NOT4-C into three regions (C1, C2, and C3) based on initial secondary structure and disorder prediction and tested them individually in MBP pull-down experiments with the NOT module or CAF40. These experiments identified the central C2 region (residues E377–Q428) (Fig. 1B) as a major interaction site for both the NOT module and CAF40 (Fig. 3D, lanes 11,16), although the interactions were weaker than with the entire NOT4-C region (Fig. 3D, lanes 9,14). The NOT module also interacted very weakly with the C3 region of *Hs* NOT4 (Fig. 3D, lane 12), whereas CAF40 showed no additional interactions in this context.

Considering the importance of the C2 region in the pull-down experiments, we also tested it in a tethering assay using the β-globin-6xMS2bs mRNA reporter. Strikingly, the C2 region was sufficient and highly efficient to trigger reporter mRNA decay. In contrast, the C1 or C3 region failed to elicit mRNA decay when tethered to the reporter (Fig. 2G,H). All tested fragments were expressed at comparable levels (Fig. 2I).

### Alignments of NOT4 proteins reveal highly conserved sequence motifs in NOT4-C

To identify a potential sequence motif that could be responsible for the interaction of the C2 region with the NOT module and CAF40, we generated and analyzed separate alignments of NOT4 proteins from metazoans, plants, and yeasts. These alignments revealed locally conserved sequences at different positions in the C-terminal regions of NOT4 (Fig. 1B; Supplemental Figs. S4, S5A; Supplemental Alignment Files SF1–SF3).

Most striking is the conservation of a 23-amino-acid motif with α-helical propensity in the C2 region of *Hs* NOT4, which is present throughout all metazoans (Fig. 1B; Supplemental Fig. S4). A similar sequence also exists in plants but is not found in fungi. Conversely, the NOT1-binding motif of *Sc* Not4 (Bhaskar et al. 2015) is conserved in yeast but cannot be identified in plants and metazoans (Supplemental Fig. S5A; Supplemental Alignment Files SF1–SF3).

Beside the 23-amino-acid motif, the alignments also uncovered the presence of a proline-rich PPPGΦ motif (Φ = F, L, or I) at least once in each of the NOT4 sequences from metazoans, plants, and yeasts. The position of the PPPGΦ motif within NOT4-C varies with the phylogeny and can be found before or after the 23-amino-acid motif with the potential for misaligning distantly related sequences (Supplemental Figs. S4, S5A; Supplemental Alignment Files SF1–SF3). PPPGΦ motifs are known to be recognized by proteins containing proline-binding GYF domains (Kofler and Freund 2006).

The 23-amino-acid motif (residues E400–E422) occupies the second half of the C2 region (Supplemental Fig. S5B). We therefore again performed MBP pull-down experiments with the NOT module and CAF40, where either the first half (C2a) or the second half (C2b) of the C2 region or the entire C2 region was deleted from *Hs* NOT4-C. These deletions did not detectably affect the interaction with the NOT module, indicating that the C1 and C3 regions of *Hs* NOT4-C together are still sufficient to pull down the NOT module (Supplemental Fig. S5C, lanes 9–12). However, the interaction with CAF40 was clearly diminished by deleting the second half of the C2 region (Supplemental Fig. S5C, lanes 15,17). This observation suggests that the 23-amino-acid motif acts as a conserved CBM. This assumption was confirmed in the following by X-ray crystallography. The motif is hence called the NOT4 CBM.

### Crystal structure of the CBM of *Dm* NOT4 in complex with *Hs* CAF40

**Figure 4.**
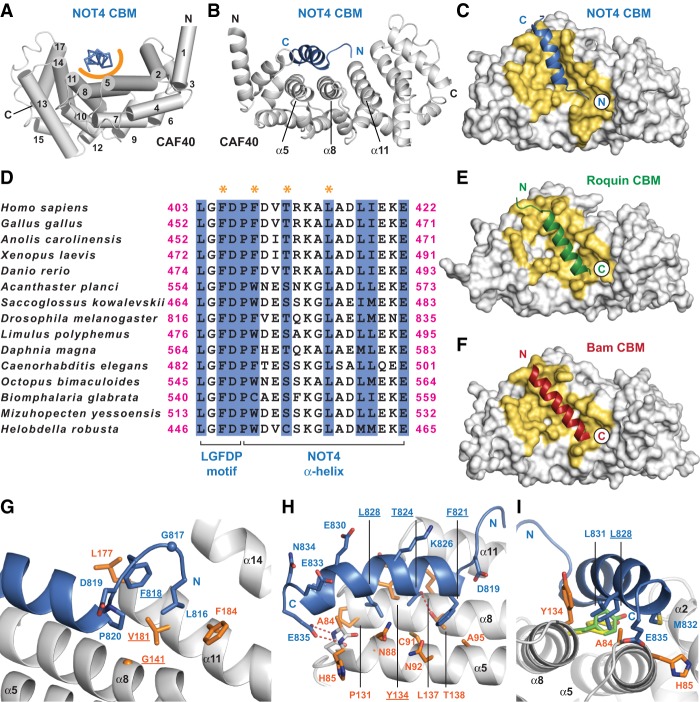
Crystal structure of the NOT4 CBM bound to CAF40. (*A*) Complex of the *Dm* NOT4 CBM peptide (blue, shown in ribbon representation) bound to *Hs* CAF40 (gray). The α helices of CAF40 are numbered and depicted as cylinders. The orange semicircle marks the predominantly hydrophobic interface between the CBM peptide and CAF40. The structurally variable flanks of the CBM peptide are excluded. (*B*) Rotated view of the CBM–CAF40 complex in cartoon representation marking the three central α helices of the concave CBM-binding surface. (*C*) Top view of CAF40 in surface representation, with the CBM of NOT4 as a cartoon. Interacting residues are colored in yellow. (*D*) Sequence alignment of metazoan NOT4 CBMs excluding the structurally variable flanks. The N-terminal portion of the CBM contains an extended LGFDP motif, and the C-terminal portion of the CBM consists of a bent α helix. Residues that directly contact CAF40 are shaded in blue, and residues that were mutated in this study are marked by orange asterisks. (*E*,*F*) Complexes of *Hs* CAF40 with the CBMs of *Dm* Roquin (*E*; Protein Data Bank [PDB] ID 5lsw) (Sgromo et al. 2017) and *Dm* Bam (*F*; PDB ID 5onb) (Sgromo et al. 2018), shown in the same style and orientation as in *C* and excluding structurally variable flanks of the CBMs. Note the inverted orientation of the CBMs. (*G*–*I*) Close-up views of the interface between *Dm* NOT4 and *Hs* CAF40. Selected side chains of NOT4 and CAF40 are shown as blue and orange sticks, respectively, with nitrogens in dark blue and oxygens in red. Hydrogen bonds are indicated by red dashed lines. Residues mutated in this study are underlined. (*I*) Rotamers of CAF40 Y134 as found in the complexes of CAF40 with the CBMs of *Dm* Roquin (yellow) and *Dm* Bam (lime).

To understand in molecular detail how NOT4 interacts with the CCR4–NOT complex, we used peptides corresponding to the putative CBM of *Hs* NOT4 or *Dm* NOT4 to set up cocrystallization trials with *Hs* CAF40. We obtained crystals—but only of a heterologous complex consisting of *Hs* CAF40 and the CBM of *Dm* NOT4. We obtained two distinct crystal forms, each with two crystallographically independent complexes per asymmetric unit and diffracting X-rays to a maximum resolution of 2.1 Å (Table 1). CBM binding is highly similar among the four CAF40–CBM complexes, and therefore only one of them (polypeptide chains A and B from space group P2_1_2_1_2_1_) is described (Fig. 4A–C; Supplemental Figs. S5D,E, S6).

**Table 1.**
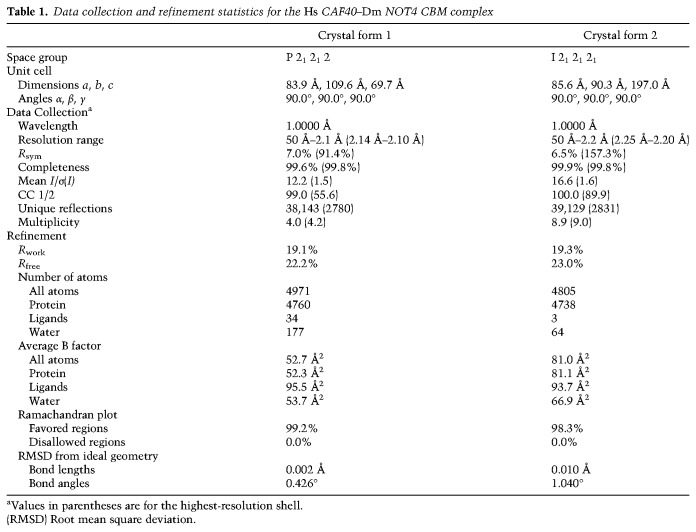
Data collection and refinement statistics for the *Hs* CAF40–*Dm* NOT4 CBM complex

From *Dm* NOT4 L816 to E835 (see Fig. 4D for human sequence numbers), the *Dm* CBM adopts a common conformation in all of the available structures, with *Dm* NOT4 P820 to E835 folding into four turns of an amphipathic α helix that is bent between turns two and three toward the surface of *Hs* CAF40. Only the very N-terminal and very C-terminal residues of the crystallized peptide show differing orientations in the four available complexes, probably due to crystal-packing interactions and indicating structural flexibility (Supplemental Fig. S5E). *Hs* CAF40 adopts its rigid and previously described crescent-like shape, made from six armadillo repeats. Despite its highly negative overall charge (pI = 3.6), the *Dm* CBM interacts with *Hs* CAF40 primarily via hydrophobic contacts. It engages the concave surface of *Hs* CAF40, contacting residues from three parallel α helices (α5, α8, and α11) and burying a surface on *Hs* CAF40 of 842 Å^2^ (Fig. 4A–C).

Importantly, the very same surface of CAF40 was described previously to be engaged also by the CBMs of Roquin and Bam (Fig. 4E,F; Sgromo et al. 2017, 2018). These CBMs also fold into amphipathic helices, covering surface areas of 841 Å^2^ and 748 Å^2^, respectively, and excluding any simultaneous associations of multiple CBMs with CAF40. Strikingly, however, whereas the CBMs of Roquin and Bam run in parallel to the α helices α5, α8, and α11, the CBM of NOT4 runs in an antiparallel fashion and hence is structurally and evolutionarily unrelated to the other two CBMs.

### Details of the interaction between the CBM of *Dm* NOT4 and *Hs* CAF40

The amphipathic α helix of the *Dm* NOT4 CBM is preceded by an “LGFDP” sequence motif that is invariant in our alignment of metazoan species (Fig. 4D). This sequence motif by itself forms a characteristic structure that helps to pin down the N-terminal half of the α helix (Fig. 4G,H). Probably due to the backbone flexibility provided by *Dm* NOT4 G817, the side chains of *Dm* NOT4 L816, F818, and P820 can join to form a small hydrophobic cluster, which is centrally contacted and completed by V181 from the α helix α11 of *Hs* CAF40. Furthermore, V181 is assisted by F184 to fix *Dm* NOT4 L816 and by L177 to fix *Dm* NOT4 F818, allowing *Dm* NOT4 L816 and F818 to intercalate between the side chains on the α helix α11 of *Hs* CAF40. *Dm* NOT4 P820 also initiates the α helix of NOT4 and is assisted by *Dm* NOT4 D819, which caps the helix and compensates for the positive charge of the helix dipole. Moreover, P820 is positioned straight over G141 in the α helix α8 of *Hs* CAF40 and would be spatially incompatible with any other residue at this position apart from a glycine.

Following P820, the hydrophobic surface of the NOT4 α helix probes the groove between α helices α8 and α5 of *Hs* CAF40 (Fig. 4D,H,I) using side chains of *Dm* NOT4 F821, T824, L828, and L831. These side chains are lined by residues from α helices α8 (*Hs* CAF40 T138, L137, Y134, and P131) and α5 (*Hs* CAF40 A95, N92, C91, N88, S87, H85, and A84), all of which are within van der Waals distance and frequently contribute to the interactions with the aliphatic portions of their side chains. At the C-terminal end of the CBM (Fig. 4D,H,I), the invariant *Dm* NOT4 E835 pins down the C-terminal half of the NOT4 α helix, using two hydrogen bonds to coordinate the free main chain nitrogens of *Hs* CAF40 A84 and H85 at the beginning of α helix α5 and compensating for the positive charge of the helix dipole. As a consequence of this interaction, the side chain of A84 gets completely surrounded by residues from NOT4 (*Dm* NOT4 L828, L831, M832, and E835), tolerating no side chains at this position that are larger than alanine. Additional specificity arises from an H bond between the side chains of *Dm* NOT4 T824 and *Hs* CAF40 T138 on α helix α8, which is deeply buried in the interface (Fig. 4D,H). Finally, it is important for *Hs* CAF40 Y134 on α helix α8 to rotate away from its preferred rotamer position that is observed in free CAF40 (Garces et al. 2007; Chen et al. 2014; Mathys et al. 2014) and in the complexes with Roquin (Sgromo et al. 2017) and Bam (Sgromo et al. 2018). Hence, the orientation of *Hs* CAF40 Y134 could help to discriminate between the three binding partners, liberating access for T824 and L828 from the *Dm* NOT4 α helix to the groove between α helices α5 and α8 (Fig. 4D,H,I). As a result, the CBM and CAF40 interact via highly complementary shapes with a hydrophobic interface that excludes any water molecule and by exposing polar residues (*Dm* NOT4 K826, E830, E833, and N834) to the solvent on the hydrophilic side of the *Dm* NOT4 α helix (Fig. 4D,H).

### Validation of the binding interface

**Figure 5.**
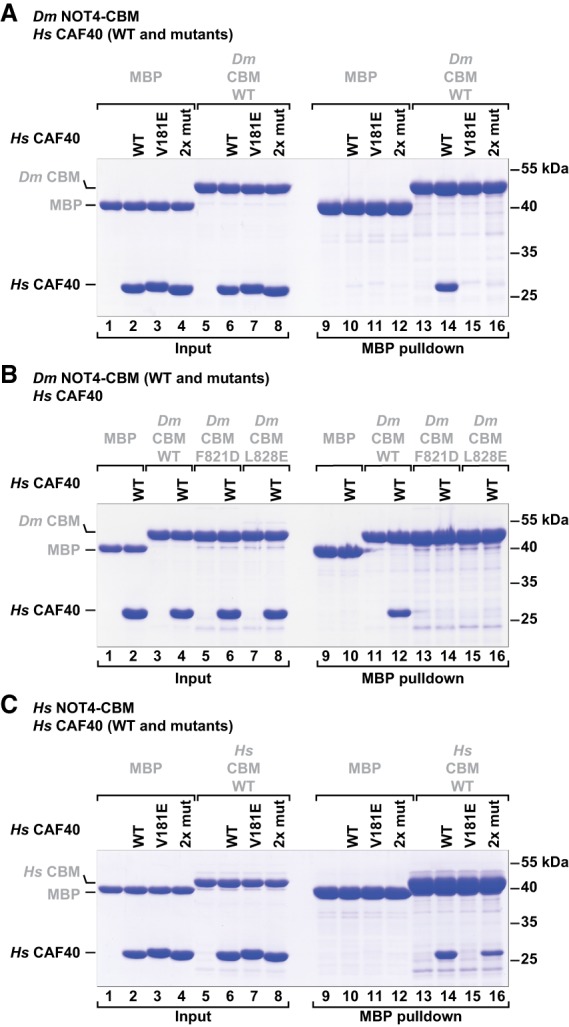
The interface between the NOT4 CBM and CAF40 is conserved in metazoans. MBP pull-down assays with MBP-tagged NOT4 CBMs and purified recombinant *Hs* CAF40 were done as described in Figure 3, A–D. (*A*) Mutants of *Hs* CAF40 in the presence of the *Dm* NOT4 CBM. Mutations target the interaction with the LGFDP motif (*Hs* CAF40 V181E) or the interaction with the CBM helix (*Hs* CAF40 2x mut; Y134D or G141W). (*B*) Mutants of the *Dm* NOT4 CBM in the presence of *Hs* CAF40. Mutations target the interaction of the CBM helix (*Dm* NOT4 F821D or L828E). (*C*) Mutants of *Hs* CAF40 in the presence of the *Hs* NOT4 CBM.

To validate the specificity of the interface observed in the crystal structure, we generated mutations in the CBM of *Dm* NOT4 and in *Hs* CAF40 and tested them in MBP pull-down assays. First, we demonstrated that the CBM of *Dm* NOT4 indeed also interacts with *Hs* CAF40 in solution (Fig. 5A, lane 14), confirming that it is a bona fide CBM. We disrupted the interaction from the side of CAF40 using either a single point mutation targeted at the “LGFDP” motif (*Hs* CAF40 V181E) (Fig. 4G) or a double point mutation targeted at the α-helical part of the CBM (*Hs* CAF40 2x mut; Y134D and G141W) (Fig. 4G,I). Both mutations had been used previously to disrupt the interactions of CAF40 with the CBMs of Roquin and Bam (Sgromo et al. 2017, 2018); in the present structural context, they abolished the interaction with the *Dm* NOT4 CBM (Fig. 5A, lanes 15,16). Conversely, single substitutions in the *Dm* NOT4 CBM (F821D or L828E) (Fig. 4D,H) were sufficient to abrogate the interaction with *Hs* CAF40 (Fig. 5B, lanes 14,16).

Furthermore, the His_6_-NusA-tagged *Dm* NOT4 CBM and the His_6_-NusA-tagged *Dm* Bam CBM competed with the MBP-tagged *Dm* NOT4 CBM for binding to *Hs* CAF40, confirming that they target overlapping binding surfaces on *Hs* CAF40 (Supplemental Fig. S5F). As in the case of the *Dm* Roquin CBM (Sgromo et al. 2018), we could not determine a precise dissociation constant for the *Dm* NOT4 CBM because it aggregated at concentrations needed to perform isothermal titration calorimetry and microscale thermophoresis experiments.

Finally, we also tested the interaction of the *Hs* NOT4 CBM with *Hs* CAF40. Similar to the results obtained with the *Dm* NOT4 CBM, the single *Hs* CAF40 V181E mutation (Fig. 4G) was sufficient to abolish the interaction with the *Hs* NOT4 CBM (Fig. 5C, lane 15 vs. 14). The double point mutation (*Hs* CAF40 2x mut) (Fig. 4G,I) also reduced the interaction but was not sufficient to abolish it, indicating species-specific adaptations in the molecular details of the coevolved interface of the human proteins (Fig. 5C, lane 16 vs. 14). Together, the results confirm that the interactions observed in the crystal structure also occur in solution.

### The CBM is essential for the interaction of NOT4 with the human CCR4–NOT complex

**Figure 6.**
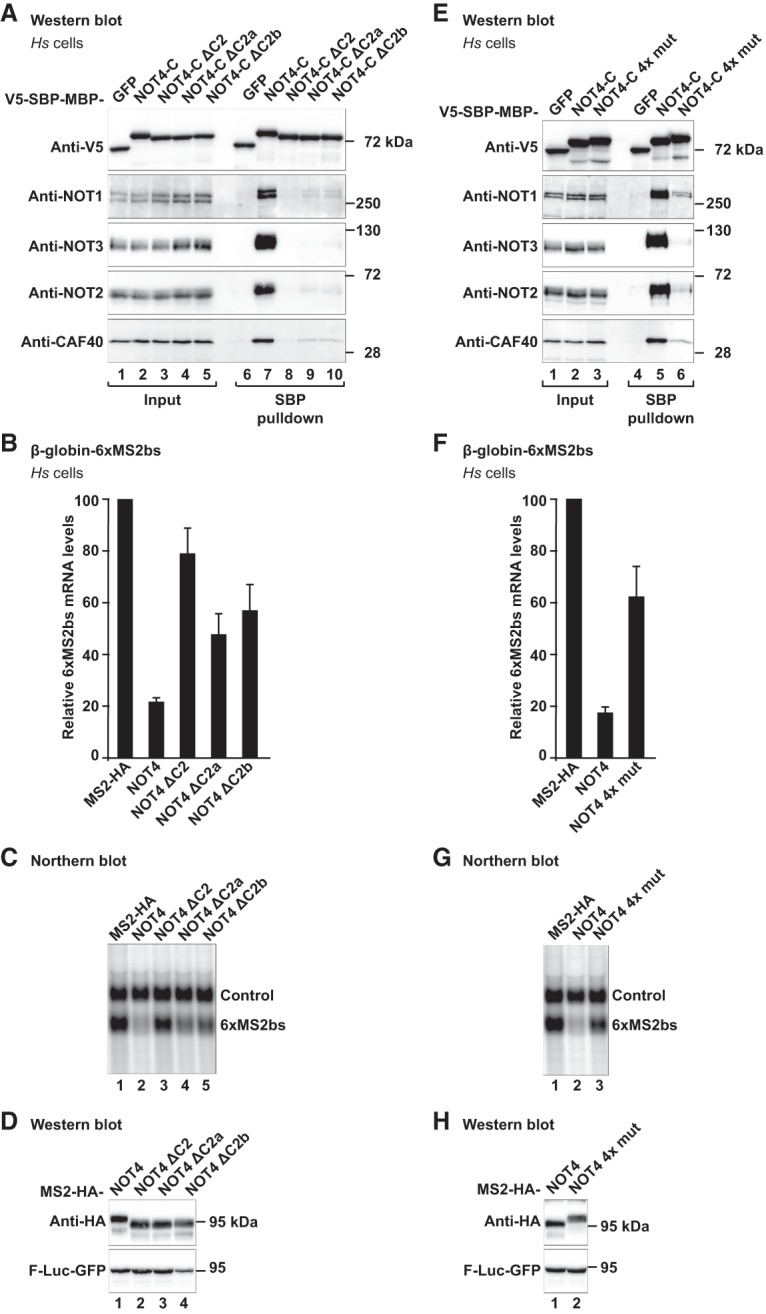
The NOT4 CBM plays a crucial role in the interaction with the CCR4–NOT complex in HEK293T cells. (*A*) SBP pull-down of endogenous human CCR4–NOT proteins with V5-SBP-MBP-tagged deletion variants of *Hs* NOT4-C. NOT4-C ΔC2 lacks residues E377–S424. NOT4-C ΔC2a lacks residues E377–D402. NOT4-C ΔC2b lacks residues E400–Q428, including the CBM. For additional details, see Figure 1C and Supplemental Figure S5B. (*B*–*D*) Tethering assay with the deletion variants of *Hs* NOT4 and the β-globin mRNA reporter. (*B*) Relative mRNA levels, with error bars corresponding to standard deviations. *n* = 3. For additional details, see Figure 2A. (*C*) Northern blot. (*D*) Western blot. (*E*) SBP pull-down of endogenous human CCR4–NOT proteins with a V5-SBP-MBP-tagged CBM mutation variant of *Hs* NOT4-C (4x mut; F405D, F408A, T411E, and L415E). For additional details, see Figure 1C. (*F*–*H*) Tethering assay with the CBM mutation variant of *Hs* NOT4 (4x mut) and the β-globin mRNA reporter. (*F*) Relative mRNA levels, with error bars corresponding to standard deviations. *n* = 3. For additional details, see Figure 2A. (*G*) Northern blot. (*H*) Western blot.

To investigate the significance of the CBM–CAF40 interaction in cells and in the context of the entire CCR4–NOT complex, we repeated SBP pull-down assays with *Hs* NOT4-C where either the CBM (ΔC2b), the C2a region (ΔC2a), or the entire C2 region (ΔC2) were deleted from the construct (see Supplemental Fig. S5B for boundaries). We found that the presence of the CBM is required to pull down the CCR4–NOT complex (Fig. 6A, lane 7), as detected by the absence of NOT1, NOT2, NOT3, and CAF40 in the pull-down fraction when the C2b region was deleted (Fig. 6A, lane 10). Consistently, the deletion of the entire C2 region as well disrupted the interaction (Fig. 6A, lane 8). Surprisingly however, the presence of the C2a region was also required for an efficient pull-down of the CCR4–NOT complex (Fig. 6A, lane 9). The C2a region could simply act as a spacer to allow for a proper interaction of the CBM with CAF40. More likely, however, its requirement reflects and underlines the importance of auxiliary and possibly species-specific interactions of NOT4-C with other parts of the CCR4–NOT complex, such as the NOT module. In line with these results, the deletion of the entire C2 region strongly diminished the NOT4-mediated degradation of the β-globin-6xMS2bs reporter mRNA in HEK293T cells (Fig. 6B,C). The separate deletion of the C2a region (ΔC2a) or the CBM (ΔC2b) did so as well but to a lesser degree. All tethered proteins were expressed at a similar level (Fig. 6D).

To probe more directly for the interface observed in the crystal structure, we engineered a quadruple point mutant of the *Hs* NOT4 CBM (4x mut; F405D, F408A, T411E, and L415E) (Fig. 4D) affecting both the LGFDP motif and the hydrophobic surface of the α helix. This mutation strongly reduced the interaction with the CCR4–NOT complex in the SBP pull-down assay (NOT4-C 4x mut) (Fig. 6E), whereas individual point mutations were not as effective (Supplemental Fig. S7A). Also in the tethering assay, the quadruple point mutation (NOT4 4x mut) impaired the ability of NOT4 to degrade the β-globin-6xMS2bs reporter mRNA to a degree that is comparable with the deletion of the entire CBM (Fig. 6, F,G vs. B,C). All of the tethered proteins were equally expressed (Fig. 6H). These findings demonstrate the importance of the CBM for the function of NOT4 and indicate that this is a major site of interaction with the CCR4–NOT complex.

### CAF40 plays a crucial and conserved role for the recruitment of NOT4 to the CCR4–NOT complex in metazoans

**Figure 7.**
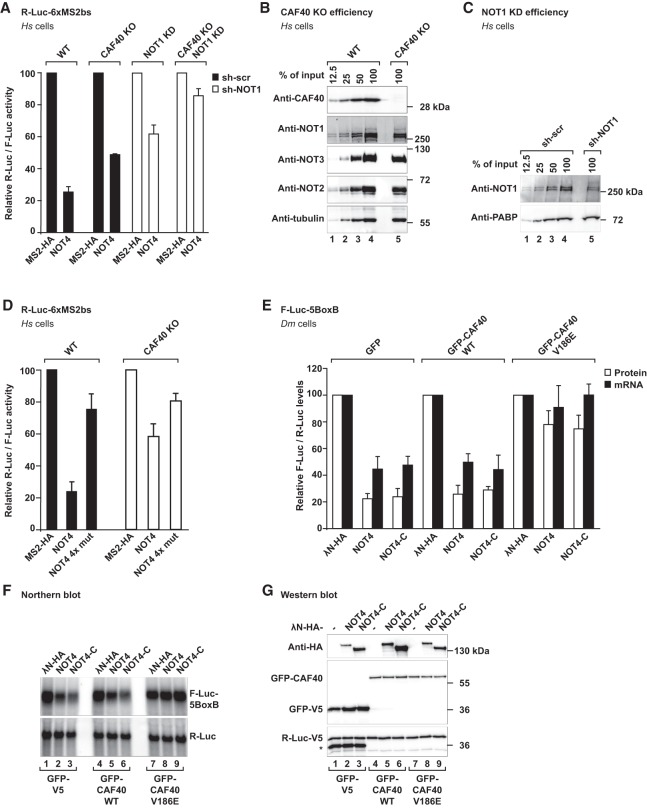
CAF40 is a central mediator for the interaction of NOT4 with the CCR4–NOT complex in HEK293T and *Dm* S2 cells. (*A*) Tethering assay with *Hs* NOT4 and a luciferase reporter in HEK293T cells lacking CAF40 (CAF40 knockout [KO]) (Sgromo et al. 2018). Renilla luciferase mRNA served as a reporter and contained six binding sites for the MS2 protein (R-Luc-6xMS2bs). Firefly luciferase mRNA served as a reference and transfection control (F-Luc). R-Luc activity was normalized to the reference and plotted with respect to the values obtained from the expression of MS2-HA alone (set to 100). The shRNA-mediated depletion of the CCR4–NOT complex is indicated by white bars (sh-NOT1 RNA and NOT1 knockdown [KD]) as compared with black bars (sh-scr RNA, control). Error bars correspond to standard deviations. *n* = 3. (*B*) Efficiency of CRISPR–Cas9-mediated gene editing of CAF40. The Western blot shows CAF40 knockout cells in comparison with a dilution series of wild-type HEK293T cells using tubulin as a loading control. (*C*) Efficiency of shRNA-mediated depletion of NOT1. The Western blot shows HEK293T cells expressing sh-NOT1 RNA in comparison with a dilution series of HEK293T cells expressing sh-scr RNA using PABP as a loading control. (*D*) Tethering assay with the CBM mutation variant of *Hs* NOT4 (4x mut) and the luciferase mRNA reporter in CAF40 knockout cells. Error bars correspond to standard deviations. *n* = 3. (*E*–*G*) Tethering assay with *Dm* NOT4 in *Dm* S2 cells overexpressing *Dm* CAF40 with a mutated CBM-binding surface. Experiments were done with λN-HA-tagged *Dm* NOT4 or *Dm* NOT4-C and analyzed as in Figure 2D, but cells were additionally overexpressing either wild-type *Dm* CAF40 or mutant *Dm* CAF40 (GFP-CAF40 V186E, corresponding to *Hs* CAF40 V181E). GFP-V5 was overexpressed as a negative control. (*E*) Relative protein and mRNA levels, with error bars corresponding to standard deviations. *n* = 3. (*F*) Northern blot. (*G*) Western blot. The asterisk denotes the additional detection of GFP-V5 on the anti-V5 blot.

The present crystal structures show how the CBM interacts with CAF40, but our previous experiments did not formally exclude that the CBM makes similarly important contacts with other subunits of the CCR4–NOT complex. Therefore, we took advantage of HEK293T cells in which CAF40 had been knocked out by CRISPR–Cas9 genome editing (CAF40 knockout cells) (Fig. 7A–D; Supplemental Fig. S7B–D; Sgromo et al. 2018). In this cell line, the levels of endogenous NOT1, NOT2, and NOT3 proteins were not altered (Fig. 7B), but the NOT4-mediated decay of the tethered R-Luc-6xMS2bs mRNA reporter (Fig. 7A,D) or of the tethered β-globin-6xMS2bs mRNA reporter (Supplemental Fig. S7B,C) was impaired. This observation underlines the importance of CAF40 for the efficient recruitment of the CCR4–NOT complex.

To test whether the remaining mRNA repression in CAF40 knockout cells (Fig. 7A; Supplemental Fig. S7B,C) was due to the CCR4–NOT complex, we additionally disrupted and depleted the remainder of the complex by a shRNA-mediated knockdown of NOT1 (Fig. 7C; Supplemental Fig. S7D; Boland et al. 2013). Under these conditions, tethering of NOT4 left reporter mRNA expression almost unaffected (CAF40 knockout + NOT1 knockdown) (Fig. 7A; Supplemental Fig. S7B,C). Consequently, the remaining subunits of the complex in CAF40 knockout cells still interact with tethered NOT4, which seems to act exclusively via the CCR4–NOT complex.

Finally, there was only a small difference between tethering the quadruple point mutation of NOT4 (NOT4 4x mut) and tethering wild-type NOT4 in CAF40 knockout cells, whereas this difference was considerable in wild-type cells (Fig. 7D). This result confirms CAF40 as the primary interaction partner of the NOT4 CBM in human cells.

The conservation of the CBM suggests that the interaction of NOT4 with CAF40 is preserved in metazoans, albeit modulated by additional contacts, such as with the NOT1-SHD (Fig. 3C). Likely due to such taxon-specific or species-specific modulation, it was therefore possible in *Dm* S2 cells to obtain a dominant-negative effect on CCR4–NOT recruitment by overexpressing a V186E mutant of GFP-tagged *Dm* CAF40 (Fig. 7E–G). This mutant (corresponding to *Hs* CAF40 V181E) was also shown previously to impair CCR4–NOT recruitment by the CBM of Bam (Sgromo et al. 2018). Apparently, in this case, the overexpression of GFP-tagged CAF40 can functionally replace the endogenous protein, and a single point mutation is then sufficient to disrupt the interaction with NOT4. Again, these observations demonstrate the central and conserved role of the CBM for the recruitment of NOT4 to the CCR4–NOT complex.

## Discussion

The present work demonstrates that metazoan NOT4 contains a conserved CBM in its variable C-terminal tail and elucidates the molecular details of the CBM–CAF40 complex. The CBM is required in cells for an efficient recruitment of NOT4 to the CCR4–NOT complex or a recruitment of the complex to NOT4-mediated cellular processes. The interaction of the CBM is assisted by auxiliary flanking sequences in NOT4-C that vary between metazoan species. These sequences also contact other subunits of the CCR4–NOT complex, such as the SHD of NOT1. From an evolutionary point of view, the CBM therefore appears to represent the ancestral mode of coupling the NOT4-dependent E3 ubiquitin ligase activity with the CCR4- and CAF1-dependent deadenylation activity of the CCR4–NOT complex. In yeast, however, the CBM seems to have become dispensable, possibly because the contacts with NOT1 are sufficient to maintain the interaction (Bhaskar et al. 2015). The conservation of the CBM marks NOT4 as a ubiquitous but apparently facultative cofactor of the metazoan CCR4–NOT complex that likely has important functions in a subset of CCR4–NOT-dependent cellular processes.

### Facultative and regulated interaction of the CCR4–NOT complex with metazoan NOT4

In *S. cerevisiae* and *S. pombe*, NOT4 copurifies with the CCR4–NOT complex (Chen et al. 2001; Stowell et al. 2016), suggesting that it is an integral component of the complex. In metazoan species, however, NOT4 is apparently not generally available to interact with the CCR4–NOT complex in a constitutive manner. This is indicated by the fact that endogenous *Hs* NOT4 or *Dm* NOT4 do not copurify with the core of the complex (Lau et al. 2009; Temme et al. 2010) and by our observation that full-length *Hs* NOT4 does not pull down the CCR4–NOT complex from HEK293T cell extracts, in contrast to *Hs* NOT4-C. However, full-length *Hs* NOT4 that was expressed in bacteria does interact with a reconstituted subassembly of the human CCR4–NOT complex.

We therefore speculate that metazoan NOT4-N somehow prevents NOT4-C from interacting with the CCR4–NOT complex, with possible assistance from posttranslational modifications or additional binding partners. For example, it is conceivable that a structural reorganization of NOT4 is required in eukaryotic cells to release the CBM for an interaction with CCR4–NOT and/or that the negatively charged CBM (pI = 4.0 in *Hs* NOT4) gets sequestered by the highly positively charged coiled-coil linker and RRM domains of NOT4-N (pI = 10.2 in *Hs* NOT4) when NOT4 is not bound to an mRNA. In this way, it would be possible to regulate the availability of the NOT4 ubiquitin ligase activity to only a subset of the CCR4–NOT-mediated cellular processes, but whether such regulation indeed exists and how it might be achieved in detail remains to be investigated.

### Mutual corecruitment of NOT4 and the CCR4–NOT complex

The widespread conservation of the CBM in NOT4 proteins reveals NOT4 as an ancient cofactor of the CCR4–NOT complex. Furthermore, we show that NOT4 is able to cause CCR4–NOT-mediated mRNA decay if tethered to an mRNA target. However, it remains unclear from these experiments whether metazoan NOT4 is needed primarily as an upstream recruitment factor that directs the CCR4–NOT complex to selective mRNA targets or rather as a downstream effector that recruits additional proteins to the CCR4–NOT complex and/or ubiquitinates nearby protein targets; e.g., to mark them for proteasomal degradation. In contrast to selective mRNA-binding proteins such as TTP, Nanos, or Roquin (Newman et al. 2016), there are currently no known RNA targets for the coiled-coil linker, RRM, or ZNF domains of NOT4; i.e., for its putative RNA-binding domains. This argues against an RNA-specific recruitment function.

In the context of cotranslational mRNA quality control, however, NOT4 could act as both an upstream recruitment factor of the CCR4–NOT complex to mRNAs with stalled ribosomes and a downstream effector for the ubiquitination and degradation of protein targets (Panasenko 2014). Additional protein-binding partners may modulate or stabilize the interactions in this case.

Finally, it is worthwhile to follow up also on the PPPGΦ motifs that we found to be highly conserved in the NOT4 proteins from metazoans, plants, and yeasts. PPPGΦ motifs tend to interact with GYF domain proteins (Kofler and Freund 2006), such as the GIGYF1/2 translational repressors (Kryszke et al. 2016; Peter et al. 2017; Amaya Ramirez et al. 2018) that were described to bind CAF40 in human cancer cells (Ajiro et al. 2009). Quite likely, therefore, the CCR4–NOT complex and NOT4 frequently support each other in a mutual corecruitment that is difficult to disentangle experimentally.

### Competition of mRNA-associated proteins for the CBM-binding site of CAF40

In most of the known cases where CCR4–NOT gets recruited to an mRNA target, the CBM-binding surface on CAF40 appears to remain available for a simultaneous recruitment of NOT4. This is, for example, the case for the TNRC6/GW182-mediated microRNA-dependent mRNA regulation, where tryptophans of TNRC6/GW182 bind to the convex side of CAF40 (Chen et al. 2014; Mathys et al. 2014). Similarly, mRNA-specific CCR4–NOT recruitment proteins such as TTP or Nanos apparently do not structurally interfere with NOT4 binding to the concave surface of CAF40 (Fabian et al. 2013; Bhandari et al. 2014; Bulbrook et al. 2018), allowing for a combinatorial mRNA regulation.

In contrast, the CBMs of Roquin and Bam were shown to target the exact same binding surface on CAF40 as the CBM of NOT4 (Sgromo et al. 2017, 2018), making their binding mutually exclusive. It is therefore possible that Roquin proteins have evolved to displace NOT4 in a context-specific manner, since they bring along their own E3 ubiquitin ligase domain. Conversely, in the case of Bam, the CBM might serve to prevent NOT4-mediated and ubiquitination-dependent processes downstream from CCR4–NOT recruitment in the germline of *D. melanogaster*. Future work will show whether such competition indeed occurs in vivo and whether there are additional CBM-containing mRNA-binding proteins in fungi, plants, or metazoans that operate in a similar manner.

Clearly, however, the present identification of a conserved CBM in the NOT4 protein underlines the role of CAF40 as a hub for peptide-mediated interactions and adds to the ever more complex regulation of mRNA expression in eukaryotic cells.

## Materials and methods

### DNA plasmid constructs

For bacterial expression of recombinant *Hs* NOT4 constructs in *E. coli*, cDNA sequences were inserted between the XhoI and NheI restriction sites of the pnEA-pM plasmid*,* resulting in fusion proteins carrying N-terminal MBP tags cleavable by the human rhinovirus 3C (HRV3C) protease and, in addition, C-terminal GB1 and hexahistidine tags. For bacterial expression of recombinant *Dm* NOT4 constructs, cDNA sequences were inserted between the XhoI and BamHI restriction sites of the pnYC-vM plasmid, resulting in tobacco etch virus (TEV) protease-cleavable MBP fusion proteins. For the expression of *Hs* NOT4 constructs in human (HEK293T) cells, cDNA sequences were inserted into the pCIneo-V5-SBP-MBP plasmid or the pcDNA3.1-MS2-HA plasmid using the XhoI and NotI restriction sites. For the expression of *Dm* NOT4 constructs in *Dm* S2 cells, cDNA sequences were inserted into the pAc5.1B-λN-HA plasmid using the NotI and BstBI restriction sites. All of the plasmid constructs generated in this study, including backbone references, are listed in Supplemental Table S1.

### MBP pull-down assays with bacterially expressed proteins

For initial pull-down assays with full-length *Hs* NOT4 and its fragments, the proteins were expressed in *E. coli* BL21 (DE3) Star cells (Invitrogen) overnight in LB medium at 20°C. Cells were homogenized in lysis buffer (50 mM Na/HEPES at pH 7.5, 300 mM NaCl, 5 µg/mL DNaseI, 1 mg/mL lysozyme, Roche “Complete” EDTA-free protease inhibitors) supplemented with 20 mM imidazole and 2 mM β-mercaptoethanol. The proteins were immobilized and isolated from the lysate on Ni-NTA resin (Qiagen) followed by elution in lysis buffer supplemented with 500 mM imidazole. They were then immobilized on 50 µL of amylose resin and incubated with an excess of purified CCR4–NOT proteins for 1 h in 500 µL of binding buffer (50 mM Na/HEPES at pH 7.5, 300 mM NaCl, 2 mM β-mercaptoethanol). Finally, the amylose beads were washed five times with binding buffer, and the proteins were eluted in 50 µL of binding buffer supplemented with 25 mM D(+)-maltose.

For pull-down assays with *Hs* and *Dm* NOT4 CBM constructs, proteins were purified from cells homogenized in lysis buffer supplemented with 2 mM DTT. Proteins were immobilized and isolated from the lysate on an amylose resin (New England Biolabs) followed by anion exchange chromatography over a HiTrap Q column (GE Healthcare) and size exclusion chromatography over a Superdex 200 26/600 column (GE Healthcare) in a buffer containing 10 mM Na/HEPES (pH 7.5), 200 mM NaCl, and 2 mM DTT. Forty micrograms of purified MBP-tagged NOT4 fragments or 20 µg of MBP were then incubated with approximately equimolar amounts of the respective purified CCR4–NOT proteins and 50 µL of amylose resin in 500 µL of binding buffer (50 mM Na/HEPES at pH 7.5, 200 mM NaCl, 2 mM DTT). After the incubation and washing steps, the proteins were eluted in 200 µL of binding buffer supplemented with 25 mM D(+)-maltose and precipitated with trichloroacetic acid.

The purifications of other human CCR4–NOT proteins, including *Hs* CAF40 for crystallization, were described previously (Petit et al. 2012; Boland et al. 2013; Bhandari et al. 2014; Chen et al. 2014; Raisch et al. 2016; Sgromo et al. 2017, 2018). The protein samples were resolved and analyzed by SDS-PAGE.

### Crystallization

*Hs* CAF40 (GPHMLE-R19–E285) (Chen et al. 2014) was mixed with a twofold molar excess of the *Dm* NOT4 CBM peptide (D813–Q838, chemically synthesized and purchased from EMC Microcollections) in 10 mM Na/HEPES (pH 7.5), 200 mM NaCl, and 2 mM DTT. Initial screens were carried out in sitting drops at 22°C by mixing 200 nL of sample solution (6 mg/mL CAF40, 1.2 mg/mL NOT4) with 200 nL of reservoir solution. Crystals appeared within 1 d in many conditions. Crystals of crystal form 1 appeared in the initial screen over a reservoir containing 0.2 M ammonium sulfate, 0.1 M Bis-Tris/Cl (pH 5.5), and 25% (w/v) PEG 3350. The crystals were cryoprotected in reservoir solution supplemented with 25% glycerol and flash-cooled in liquid nitrogen. Optimized crystals of crystal form 2 grew at 18°C in hanging drops mixing 1 µL of sample solution and 1 µL of reservoir solution containing 0.9 M K_2_HPO_4_ and 0.3 M NaH_2_PO_4_. Crystals were cryoprotected in 4.0 M sodium formate and flash-cooled in liquid nitrogen.

### Data collection and structure determination

X-ray diffraction data were collected at a wavelength of 1.0000 Å on a Pilatus 6M detector (Dectris) at the PXII beamline of the Swiss Light Source (Villigen) and processed using XDS and XSCALE (Kabsch 2010). Crystal form 1 (space group P2_1_2_1_2) diffracted X-rays to a resolution of 2.1 Å, whereas crystal form 2 (space group I2_1_2_1_2_1_) diffracted X-rays to a comparable resolution of 2.2 Å but with an increased B_Wilson_ (56.7 Å^2^ vs. 39.2 Å^2^). For each crystal form, we identified two copies of *Hs* CAF40 per asymmetric unit by molecular replacement using PHASER (McCoy et al. 2007) from the CCP4 package (Winn et al. 2011) and using chain A of Protein Data Bank (PDB) ID 2fv2 (Garces et al. 2007) as a search model. Initial models were improved and completed by iterative cycles of model building in COOT (Emsley et al. 2010) and refinement using PHENIX (Afonine et al. 2012). The NOT4 CBM peptides were then built manually into the remaining electron density and improved by additional building and refinement cycles. For crystal form 1, final refinement rounds were done using PHENIX with one TLS group per polypeptide chain and including small molecule ligands (one molecule each of Tris and glycerol plus four sulfate ions) in addition to 177 water molecules. This resulted in an R_work_ of 19.0% and an *R*_free_ of 21.8%. For crystal form 2, final refinement rounds were done using BUSTER (https://www.globalphasing.com/buster), also with one TLS group per polypeptide chain but in addition to small molecule ligands (one sodium and two chloride ions) and 64 water molecules, also autorefining NCS restraints. This resulted in an *R*_work_ of 19.3% and an *R*_free_ of 23.0% (Table 1). Illustrations were prepared in PyMOL (http://www.pymol.org).

### SBP pull-down assays from HEK293T cells

HEK293T cells were seeded in 10-cm dishes (4 × 10^6^ cells per plate and experiment) and transfected with pCIneo-V5-SBP-MBP plasmids after 1 d using Turbofect (Thermo Scientific) according to the manufacturer's protocol. Two days after transfection, the cells were lysed on ice in 1 mL of NET lysis buffer (50 mM Tris/Cl at pH 7.5, 150 mM NaCl, 0.1% Triton X-100, 1 mM EDTA, 10% glycerol) supplemented with protease inhibitors (Roche). After 15 min, lysates were centrifuged at 20,000*g* for 15 min at 4°C. The cleared lysate was then treated with 200 µg/mL RNase A (Qiagen) for 30 min at 4°C and centrifuged again as before, resulting in the input fraction for the experiment (1 mL = 100%). The input fraction was then incubated for 1 h at 4°C with 50 µL of streptavidin sepharose resin (GE Healthcare). The beads were washed three times with NET buffer and finally resuspended in protein sample buffer, resulting in the pull-down fraction (100 µL = 100%). The samples were analyzed by Western blot (for antibodies, see Supplemental Table S2) using the ECL Western blotting detection system (GE Healthcare).

### Tethering assays in HEK293T cells

For MS2-dependent tethering assays with the β-globin mRNA reporter (Lykke-Andersen et al. 2000), HEK293T cells were seeded in six-well plates (0.7 × 10^6^ cells per well) and transfected on the following day using Lipofectamine 2000 (Invitrogen). The transfection mixtures contained 0.5 μg of the β-globin reporter plasmid encoding six MS2-binding sites (β-globin-6xMS2bs); 0.5 μg of the β-globin reference and transfection control plasmid lacking MS2-binding sites and containing a partial sequence of GAPDH (control; β-globin-GAPDH) (Lykke-Andersen et al. 2000); and variable amounts (0.05–0.75 μg) of pcDNA3.1-MS2-HA plasmids (Supplemental Table S1) to achieve equivalent expression of MS2-HA fusion proteins. The cells were harvested 2 d after transfection. The total RNA was isolated using the peqGOLD TriFast reagent (Peqlab) and analyzed by Northern blot as described previously (Behm-Ansmant et al. 2006). Equivalent expression of MS2-HA-tagged proteins was tested in parallel by Western blot, expressing F-Luc-GFP (Kuzuoğlu-Öztürk et al. 2016) as a transfection control.

For the experiment shown in Supplemental Figure S3, A–C, cells were additionally cotransfected with 0.5 µg of a plasmid expressing either wild-type *Hs* DCP2 (GFP-DCP2 wild-type) or the *Hs* DCP2 mutant (GFP-DCP2 mut; E148Q) (Loh et al. 2013). Equivalent expression of the GFP-tagged proteins was tested in parallel by Western blot, expressing V5-SBP-MBP as a transfection control.

For MS2-dependent tethering assays with the luciferase reporter system (Kuzuoğlu-Öztürk et al. 2016), the transfection mixtures contained 0.2 µg of reporter plasmid containing or lacking six MS2-binding sites (R-Luc-6xMS2bs or R-Luc), 0.2 µg of reference and transfection control plasmid lacking six MS2-binding sites (F-Luc-GFP), and variable amounts of pcDNA3.1-MS2-HA plasmids (0.1–1.5 μg) (Supplemental Table S1). The cells were harvested 2 d after transfection, mRNA levels were determined by Northern blot, and R-Luc and F-Luc activities were measured using a “dual-luciferase reporter assay” system (Promega).

### Tethering assays in HEK293T cells with knockdown of NOT1

The shRNA-mediated depletion of NOT1 has been described previously (Boland et al. 2013) using shRNA (*Hs* NOT1 target: ATT CAACATTCCCTTATA) and control shRNA (scr, control target: ATTCTCCGAACGTGTCACG). For tethering assays in cells depleted of NOT1, wild-type HEK293T cells or HEK293T CAF40 knockout cells (Sgromo et al. 2018) were transfected twice. For the first transfection, cells were seeded in six-well plates (0.7 × 10^6^ cells per well) and transfected on the following day with mixtures containing 4 μg of plasmid expressing the respective shRNA. After 1 d, cells were selected for 24 h in DMEM supplemented with 1.5 µg/mL puromycin and subsequently seeded in six-well plates in medium without puromycin (0.7 × 10^6^ cells per well). The following day, cells were transfected again with mixtures containing 2 µg of the respective shRNA plasmids but also containing the reporter and reference/transfection control plasmids (0.2 μg of R-Luc-6xMS2bs and 0.2 μg of F-Luc-GFP) and 0.125–0.25 μg of pcDNA3.1-MS2-HA plasmids (Supplemental Table S1). After 1 d, cells were selected for 48 h in DMEM (supplemented with 1.5 µg/mL puromycin) and analyzed as before.

### Tethering assays in *Dm* S2 cells

For the λN-dependent tethering assay (Behm-Ansmant et al. 2006) with the luciferase reporter system in *Dm* S2 cells, cells were seeded in six-well plates (2.5 × 10^6^ cells per well) and transfected just thereafter using Effectene (Qiagen). The transfection mixtures contained 0.1 μg of the F-Luc-5BoxB reporter plasmid, 0.4 μg of an R-Luc reference and transfection control plasmid encoding a deadenylation-resistant mRNA lacking BoxB sequences (R-Luc; R-Luc-A_90_-HhR) (Raisch et al. 2016), and variable amounts (0.01–0.08 μg) of pAC5.1B-λN-HA plasmids (Supplemental Table S1) to achieve equivalent expression of λN-HA-fusion proteins. The cells were harvested 3 d after transfection and analyzed as described.

For the experiments in Supplemental Figure S3, D–F, *Dm* S2 cells were additionally cotransfected with 1 µg of a plasmid expressing either wild-type *Dm* DCP1 (GFP-DCP1 wild-type) or the *Dm* DCP1 mutant (GFP-DCP1 mut; R70G, L71S, N72S, and T73G) (Kuzuoğlu-Öztürk et al. 2016), and, for the experiments in Figure 7, E–G, cells were cotransfected with 1.5 µg of a plasmid expressing either wild-type *Dm* CAF40 (GFP-CAF40 wild-type) or the *Dm* CAF40 mutant (GFP-CAF40 V186E) (Sgromo et al. 2018).

### Accession numbers

Atomic coordinates and structure factors for the reported crystal structures have been deposited with the PDB under accession number 6hom for space group P2_1_2_1_2 and 6hon for space group I2_1_2_1_2_1_.

## Supplementary Material

Supplemental Material
