# Supplementary material for: A conserved CAF40-binding motif in metazoan NOT4 mediates association with the CCR4–NOT complex
Source: Genes Dev. 2019 Feb 1;33(3-4):236–52. doi: 10.1101/gad.320952.118 (PMC6362812; doi:10.1101/gad.320952.118)
Supplement: Supplemental Material [file supp_33_3-4_236__index.html]

A conserved CAF40-binding motif in metazoan NOT4 mediates association with the CCR4–NOT complex — Supplemental Material 

# A conserved CAF40-binding motif in metazoan NOT4 mediates association with the CCR4–NOT complex

## Supplemental Material

- Supplemental\_Material.pdf
- Supplemental\_Alignment\_File\_SF3.zip
- Supplemental\_Alignment\_File\_SF1.zip
- Supplemental\_Alignment\_File\_SF2.zip
