# Supplementary material for: A conserved CAF40-binding motif in metazoan NOT4 mediates association with the CCR4–NOT complex
Source: Genes Dev. 2019 Feb 1;33(3-4):236–52. doi: 10.1101/gad.320952.118 (PMC6362812; doi:10.1101/gad.320952.118)
Supplement: Supplemental Material [file supp_gad.320952.118_Supplemental_Material.pdf]

A conserved CAF40 binding motif in metazoan NOT4 mediates association with the CCR4-NOT complex

Csilla Keskeny, Tobias Raisch, Annamaria Sgromo, Cátia Igreja, Dipankar Bhandari*, Oliver Weichenrieder* and Elisa Izaurralde†

Department of Biochemistry, Max Planck Institute for Developmental Biology, Max-Planck-Ring 5, D-72076 Tübingen, Germany
* To whom correspondence should be addressed. Tel: +49-7071-601-1358, Fax: +49-7071-601-1353;
Email: oliver.weichenrieder@tuebingen.mpg.de or dipankar.bhandari@tuebingen.mpg.de
† deceased 30 April 2018

Present Address:
Tobias Raisch, Department of Structural Biochemistry, Max Planck Institute of Molecular Physiology, Otto-Hahn-Strasse 11, D-44227 Dortmund, Germany.
Annamaria Sgromo, IMBA - Institute of Molecular Biotechnology GmbH, Dr. Bohr-Gasse 3, 1030 Vienna, Austria.

- Supplemental Material -

Supplemental figures S1–S7

Supplemental tables S1–S2

Supplemental alignment files

Supplemental references

Supplemental Figure S1. The presence of the positively charged CC linker and RRM domain prevents NOT4-C from interacting with the CCR4-NOT complex

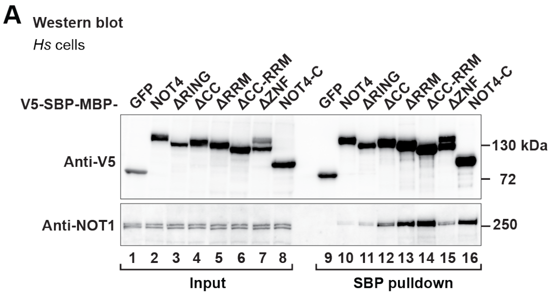

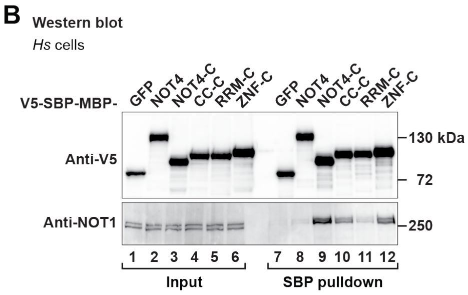

Supplemental Figure S1. The presence of the positively charged CC linker and RRM domain prevents NOT4-C from interacting with the CCR4-NOT complex

(A) SBP pulldown of endogenous human NOT1 from HEK293T cell lysates with V5-SBP-MBP-tagged deletion variants of *Hs* NOT4. V5-SBP-GFP-MBP served as negative control. Input samples correspond to 3 % of the total lysate for V5-tagged proteins and to 2 % for NOT1. Pulldown samples correspond to 7 % of the total pulldown for V5-tagged proteins and to 35 % for NOT1. For abbreviations and construct details see Figure 1B and Supplemental Table S1.

(B) SBP pulldown of endogenous human NOT1 from HEK293T cell lysates with V5-SBP-MBP-tagged fragments of *Hs* NOT4-N fused to *Hs* NOT4-C. CC-C: Putative coiled coil region fused to NOT4-C; RRM-C: RRM domain fused to NOT4-C; ZNF-C: zinc-finger domain fused to NOT4-C.

Supplemental Figure S2. Metazoan NOT4 induces degradation of tethered mRNA reporters

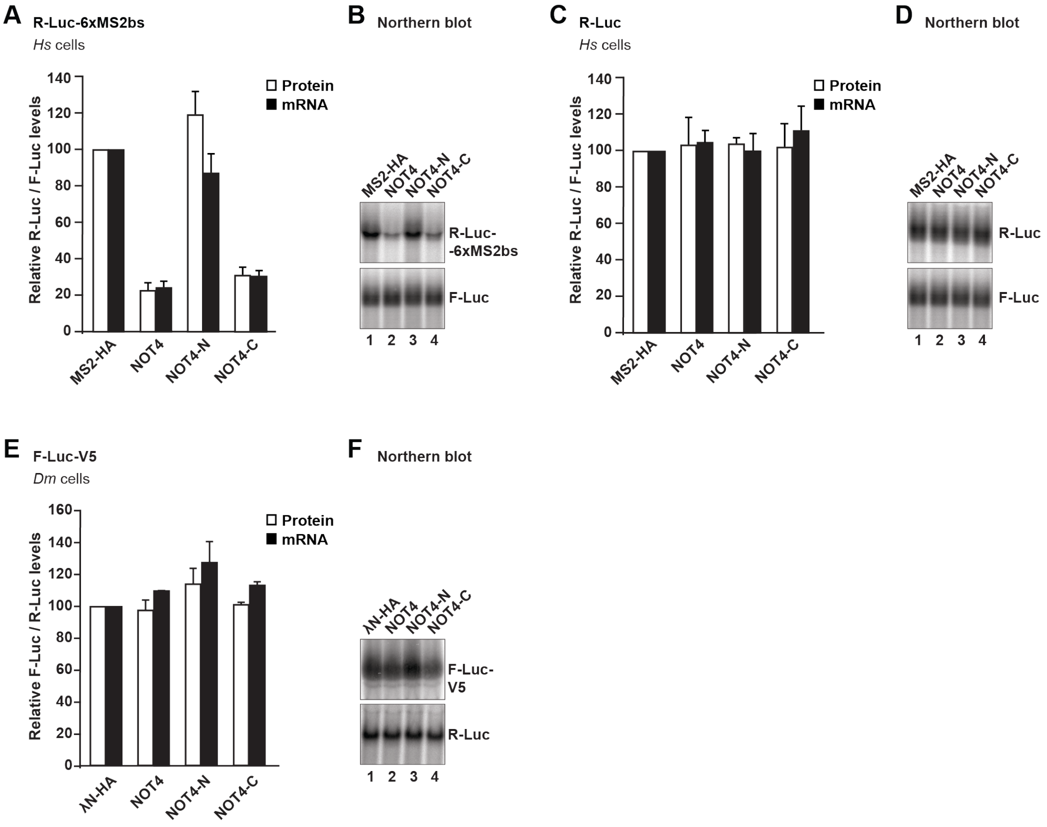

Supplemental Figure S2. Metazoan NOT4 induces degradation of tethered mRNA reporters

(A, B) Tethering assay with *Hs* NOT4 and a luciferase reporter in HEK293T cells. *Hs* NOT4 or its fragments carried an N-terminal MS2-HA tag. Renilla luciferase mRNA served as a reporter and contained six binding sites for the MS2 protein (R-Luc-6xMS2bs). Firefly luciferase mRNA served as a reference and transfection control (F-Luc). (A) R-Luc activity (white bars) and mRNA levels (black bars) normalized to the reference and plotted with respect to the values obtained from the expression of MS2-HA alone (set to 100). Error bars correspond to standard deviations (n=3). (B) Representative Northern blot.

(C, D) Control experiment for the tethering assay in panels (A, B). Renilla luciferase mRNA without binding sites for the MS2 protein (R-Luc) served as a tethering control. (C) Relative protein and mRNA levels analyzed as described in (A). (D) Northern blot.

(E, F) Control experiment for the tethering assay in Figure 2D, E with *Dm* NOT4 and a luciferase reporter in *Dm* S2 cells. Firefly luciferase mRNA without BoxB binding sites (F-Luc-V5) served as a tethering control. (E) Relative protein and mRNA levels analyzed as described in Figure 2D. (F) Northern blot.

Supplemental Figure S3. Tethered NOT4 causes reporter mRNA degradation via the 5’-to-3’ decay pathway

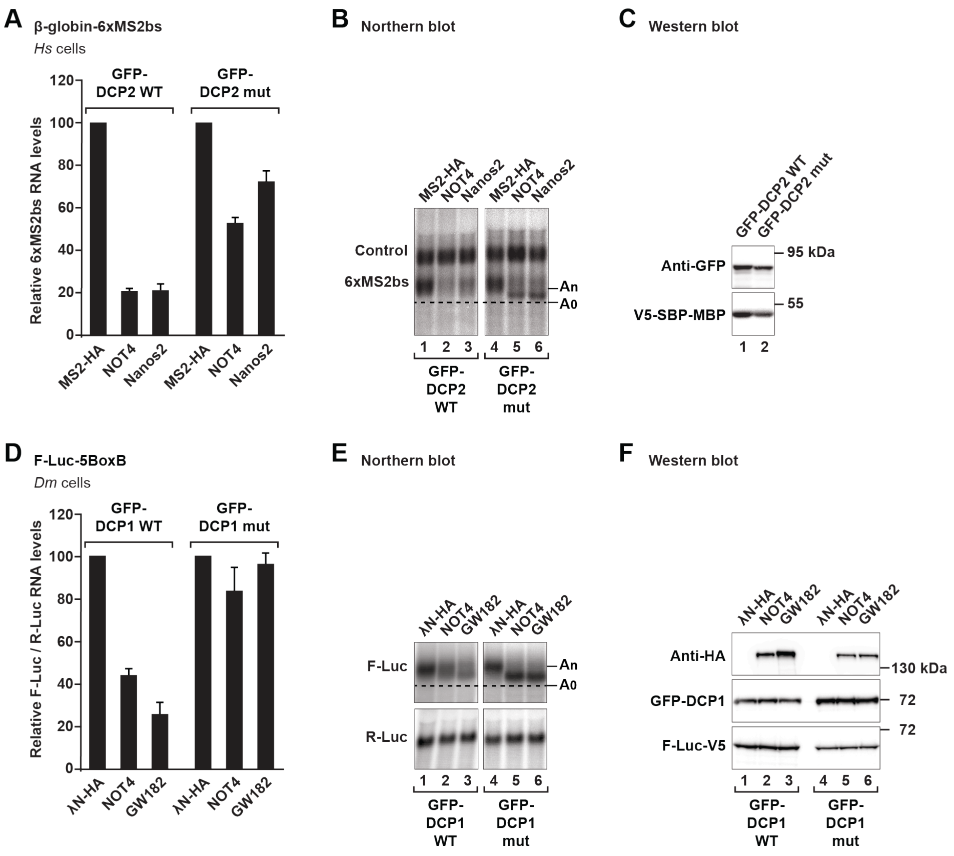

Supplemental Figure S3. Tethered NOT4 causes reporter mRNA degradation via the 5’-to-3’ decay pathway

(A–C) Tethering assay with *Hs* NOT4 and the β-globin mRNA reporter in HEK293T cells overexpressing a DCP2 catalytically inactive mutant. Experiments were done and analyzed as described in Figure 2A, but cells were additionally overexpressing either wildtype *Hs* DCP2 (GFP-DCP2 WT) or the *Hs* DCP2 mutant (GFP-DCP2 mut; E148Q). MS2-HA-tagged *Hs* Nanos2 was overexpressed as a positive control for a protein eliciting 5’-to-3’ mRNA decay (Bhandari et al. 2014). (A) Relative mRNA levels with error bars corresponding to standard deviations (n=3). (B) Northern blot demonstrating the accumulation of deadenylated mRNA degradation intermediates (A0) as compared to polyadenylated mRNA (An). (C) Western blot demonstrating equivalent expression of GFP-tagged DCP2 proteins with V5-SBP-MBP as a transfection control.

(D–F) Tethering assay with *Dm* NOT4 in *Dm* S2 cells overexpressing a DCP1 mutant. Experiments were done and analyzed as described in Figure 2D, but cells were additionally overexpressing either wildtype *Dm* DCP1 (GFP-DCP1 WT) or the *Dm* DCP1 mutant (GFP-DCP1 mut; R70G, L71S, N72S, T73G). λN-HA-tagged GW182 was overexpressed as a positive control for a protein eliciting 5’-to-3’ mRNA decay (Kuzuoğlu-Öztürk et al. 2016). (D) Relative mRNA levels with error bars corresponding to standard deviations (n=3). (E) Northern blot demonstrating the accumulation of deadenylated mRNA degradation intermediates (A0) as compared to polyadenylated mRNA (An). (F) Western blot demonstrating equivalent expression of GFP-tagged DCP1 proteins and λN-HA-tagged proteins (NOT4 and GW182), with F-Luc-V5 as a transfection control.

Supplemental Figure S4. Sequence alignment of metazoan NOT4

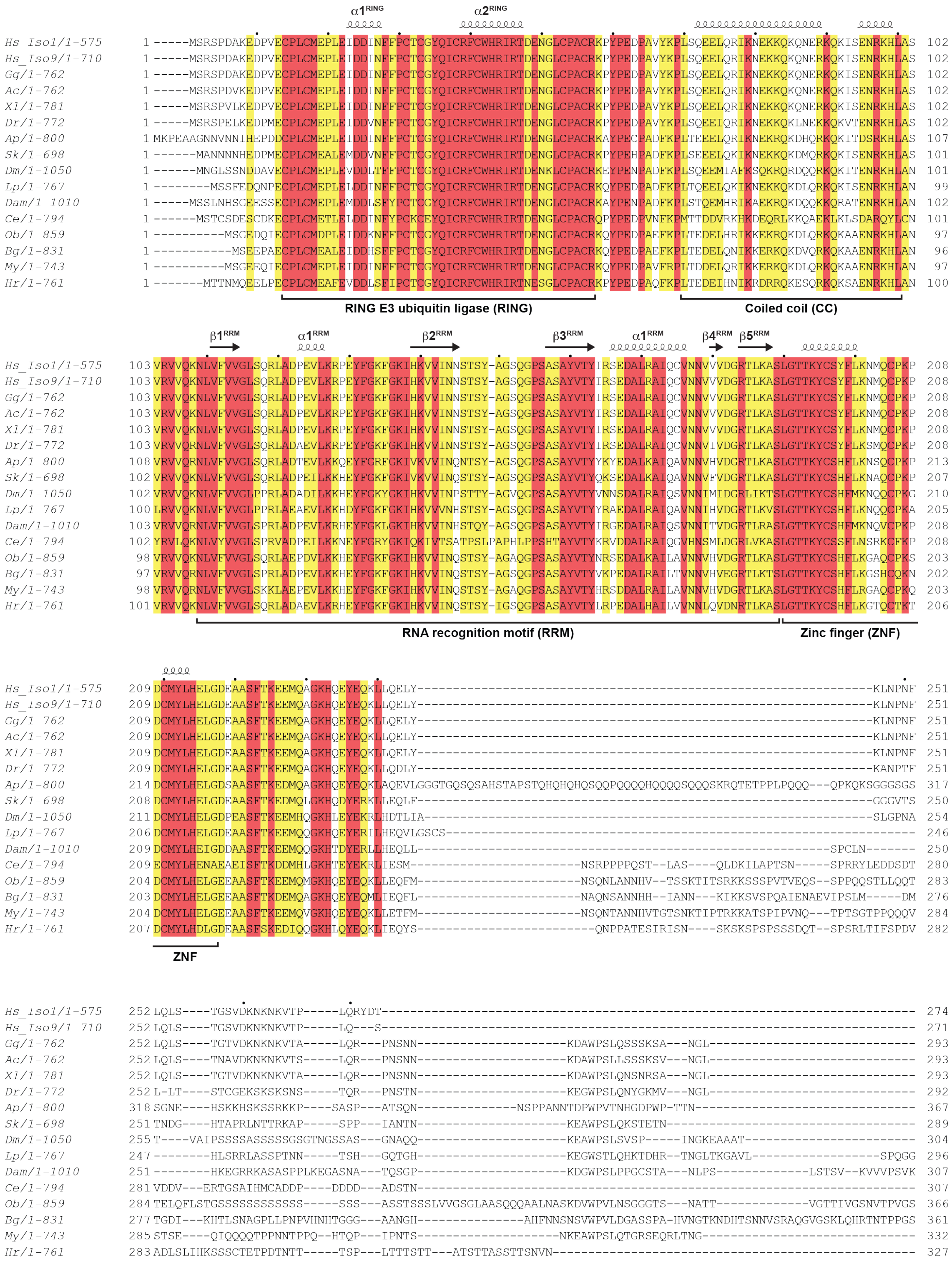

Supplemental Figure S4, continued. Sequence alignment of metazoan NOT4

```
Hs_Iso1/1-575   275 ----------------------------------------------------------------------------PIDK----------------PSDSLSIGNG- 288
Hs_Iso9/1-710   272 ----------------------------------------------------------------------------PIDK----------------PSDSLSIGNG- 285
Gg/1-762        294 -----------------TMEHRKTPPILENGTD-------SEHMTPDGADSDFG----------------------PIDK----------------PSDSLSIGNG- 337
Ac/1-762        294 -----------------TMEHRKTPPILENGTD-------SEHMTPDGPDSDFG----------------------PVDK----------------PSDSLSIGNC- 337
Xl/1-781        294 -------------------EHRKSPPILDNGLD-------PDHMTPDGPDSDFGLFWESAEHNVAKFGRGIEDDTSSIDK----------------SSESLSIGNG- 357
Dr/1-772        293 -----------------TTEHRKSPPLLDCLTD-------SDHMTPDEPDLEQGT---EQNTGLPPFPSALE-PTSPIDK----------------PSEPISIGNG- 354
Ap/1-800        368 ---SSDTWPALAGQAKSSEPYSQSPEDTPPLV-----------------------------------------SNHTPETLGQ------------SPPTGISIQTK- 417
Sk/1-698        290 ----------------------------------------KQPPSPPHPQLPQEM--------SPTL------VPNDIDD----------------INHTVTVGSPP 326
Dm/1-1050       305 --ATSSSGKSKREKLRNEKRHEKNKAKNKNGSN------TNANASNKENYVPETR------------------SSTSTETFAEATADAPASTKA-EPPQASSNRTRA 384
Lp/1-767        297 G-----QKSLGGGR-QSSKKHDSGEE--------------GRKGKGRERSLEESL------------------SRGSSSH--------------------------- 338
Dam/1-1010      308 SEPPLTKEKSGKIKAKPTQQPTVHLP--------------VAVVQPEEVTLPQAK------------------ANGSNRKTKEKTVVTAVLTAVLNPTAPVAAQPT- 381
Ce/1-794        308 -------------------------------------------------------------------------TSNSIDD----------------PAPSNPADETV 325
Ob/1-859        367 K-ATSAERILNGNKVPNTTRHHTHRPSTPTKQNKCL----RDKRDPDKLSMVENY------------------SNGSIDS----------------PNIRVSTSSSY 434
Bg/1-831        362 GGGSSASRAVGNGRQRGSNSLNSSEENHIQNQI-NVGSGNRSPTSTPPGDVEKNS------------------PNDQVNH---RGLP--------LPTPSTSQPVSP 438
My/1-743        333 ----------------NRPNHNQQKPDTQPLPNHNRAPGNHKARTKTEADIENNA------------------LNNTIDK----------------PTDTSAKSDIP 389
Hr/1-761        328 -----------------AHVHNGLLDNIDYIENSNR------------IQLLDDL------------------ANSTTST----------------STTASSIVET- 370
```

```
Hs_Iso1/1-575   289 --------DNSQQISNSDTPSPPPGLSKS--------------NPVIPISSSNHSAR-------------------------------------------------- 323
Hs_Iso9/1-710   286 --------DNSQQISNSDTPSPPPGLSKS--------------NPVIPISSSNHSAR-------------------------------------------------- 320
Gg/1-762        338 --------DSSQQITNSDTPSPPPGLTKP--------------NPVIPISSSNHSAR-------------------------------------------------- 372
Ac/1-762        338 --------DNSQQISNSDTPSPPPGLSKS--------------NSVIPISSSNHSAR-------------------------------------------------- 372
Xl/1-781        358 --------DNLQQILTSDTPSPPPGLSKP--------------NPSAPISSANHSAR-------------------------------------------------- 392
Dr/1-772        355 --------ENISQTSSSDSPSPPPGLTKP--------------SLVVPISVAELTAR-------------------------------------------------- 389
Ap/1-800        418 ----GVRQPSTEGLPPPSPEQSQPVPTGS--------------------TSSQSNQK-------------------------------------------------- 450
Sk/1-698        327 CNHQLPSPRGDLHITTNGIATTNGIRTTNGILTTNDTLLTTQIPPTVEPLARSIEQRI------------------------------------------------- 384
Dm/1-1050       385 DRGKDRTTASAKEQKKSKEAAPAPAASKPA-------ERVETSESTIRQKKAEVTESCEDNLPQ--------------------------------KRLAGTNVQRS 452
Lp/1-767        339 -----SNSSSRESLHTFRSQATPPVPSFPQ------------QSWSREPDSNPGTNE-------------------------------------------------- 378
Dam/1-1010      382 ----KPERQRSTSLSPALSTSSNPTPTLI--------------SSSSSPSSSQDSGFLPSNQHEFEILKQGASMAAEEEEIDRARDEADDYFDPAGNKVSG-NSASF 469
Ce/1-794        326 NSRRSNQTARERQWSERDEISVAPSNTPP-----------------TDPEGNENEFE---------------------------------------EDIHR-NDVSD 375
Ob/1-859        435 ---SAPSPTSAVHMNDAFATAMNPSLTF---------------------MGINQVSR-------------------------------------------------- 467
Bg/1-831        439 ---RAGGSITTSQMTTPNSNTVPIISTQ-----------------LQQQQHADLSSTVGA-------------------------------------------IGSN 482
My/1-743        390 ---TVSPPQSTSSLGTGSPVSLPPGLTTS--------------THSKAPLGAQLTARLMANPGL------------------------------------------- 436
Hr/1-761        371 ----QVKSYTTANNNTASEAVEPTTTTT---------------------TSDEQPQQ-------------------------------------------------- 402
                                         PPPGL
```

```
Hs_Iso1/1-575   324 --SPFEGAVTE-SQSLFSD--NFRH----------------------------------P-NPIP-----SGLPPFP------------------------SSPQTS 361
Hs_Iso9/1-710   321 --SPFEGAVTE-SQSLFSD--NFRH----------------------------------P-NPIP-----SGLPPFP------------------------SSPQTS 358
Gg/1-762        373 --SPFEGAVTE-SQSLFSD--NFRH----------------------------------P-NPIP-----SGLPPFP------------------------SSPQTS 410
Ac/1-762        373 --SPFEGAITE-SQSLFSD--NFRH----------------------------------P-NPIP-----SGLPPFP------------------------SSPQTS 410
Xl/1-781        393 --SPFEDAMTE-SQSLFSD--NFRH----------------------------------P-NPIP-----SGLPPFP------------------------SSPQTS 430
Dr/1-772        390 --SPFEGAAAE-SQSLFSDNSNFRH----------------------------------P-NPIP-----SGLPPFS------------------------NSPQGA 429
Ap/1-800        451 --SSFEADKSL-PSFLAAAN-GYMN-----------------------------------QGVLP-----GLAPPHPHVQPPVNGSGFQGGSTVPPLIPPRQGPTSA 513
Sk/1-698        385 --SPFNGLHTDNSSSLFSN--TFMP----------------------------------PLRPVP--------PPPV------------------------SVPNAN 421
Dm/1-1050       453 VSSCSENSEGHVSESSLSEK-SLTGDYVEEKCNSVNSESQQESVKFQEELEKSNEAIVEAETILPTAESSEDISPAAVAPG--NGE----VEGCLPVVDPVEPPSLA 552
Lp/1-767        379 --ETLEPSDCKAESSTLQAY----------------------------------------CNIIE-----NQSPPMC----------------ETPKLDSLQNEPTSA 423
Dam/1-1010      470 AHSILDTNNSSNSHSFFSTG--------------------------------------------P-------LPAYT-AQS--NG----------------TSPSGC 506
Ce/1-794        376 LMSKLDVNDDRLARTSFSEN-DYLG--------------------------------------IP-APAKHQEAPAPLMQW--------------EALLGLSSPSAQ 428
Ob/1-859        468 --QVLEKRNAD-SLSFFSGN-GFSSG------------SERETTKNS--------TIQHS-TGIP-----ESLP--------------------------------- 511
Bg/1-831        483 RRPGLQEGNSAQGLSFFEM------------------------------------------------------------------------------------PNVS 505
My/1-743        437 -KPILFDNPAQ-SLSLFSNGNGFHG---------------KRFQKL-------------PVPTVP-----AEVPEPV------------------------------ 478
Hr/1-761        403 ------------HGFLLSN---------------------------------------------------------------------------------------- 409
```

```
Hs_Iso1/1-575   362 -------------------SDWPTAPEPQ----------SLFTSETIP-----------VSSSTDWQAAF------------------------------------- 391
Hs_Iso9/1-710   359 -------------------SDWPTAPEPQ----------SLFTSETIP-----------VSSSTDWQAAF------------------------------------- 388
Gg/1-762        411 -------------------NDWPTAPEPQ----------SLFTSETIP-----------VSSSTDWQAAF------------------------------------- 440
Ac/1-762        411 -------------------NDWPMAPEPQ----------SLFTSETIP-----------VSSSTDWQAAF------------------------------------- 440
Xl/1-781        431 -------------------SEWPTAPEPQ----------SLFTSETIP-----------VSSSTDWQAAF------------------------------------- 460
Dr/1-772        430 -------------------SDWPMTPEPQ----------SLFTSETIP-----------VSSSTDWQAAF------------------------------------- 459
Ap/1-800        514 -------------------GDWSEG--------------VILNNDLLP-----------VASHTDWQAAF------------------------------------- 539
Sk/1-698        422 TL--------------PTTNDW------Q---------DSMFGNDTLP-----------ISSSTNWQEAF------------------------------------- 451
Dm/1-1050       553 DNGSRVTDALSKLNIFDDTPSFFTSPSFQQAPILKNKLDLEMRQSHLPDLVNDIDGIQKASNTNEWEEAFKNVMMGNTQHMEEQLLQQQHLQQHQNLRHQLVLQQEE 659
Lp/1-767        424 LSTYK--------ELDDSEEDWLNHP------------DLAFHSESIP-----------VNSSTDWQAAF------------------------------------- 462
Dam/1-1010      507 ---------------------WLANGA-----------EEEAITSTLP-----------NQSAADWQLAF------------------------------------- 533
Ce/1-794        429 ST-------------------------------------IVEPSSLFPTF--------KMDSGFNSQSLF------------------------------------- 453
Ob/1-859        512 ---------------------------------------SLEIPDTIP-----------VTSATDWQAAF------------------------------------- 531
Bg/1-831        506 LV-------------------------------------NHEMADTLP-----------VSTSTDWVEAF------------------------------------- 527
My/1-743        479 --------------------------------------STPEIAESIQ-----------VTSCTDWQAAF------------------------------------- 499
Hr/1-761        410 ----------------------------------------------VD-----------SSSPTNENANT------------------------------------- 422
```

Supplemental Figure S4, continued. Sequence alignment of metazoan NOT4

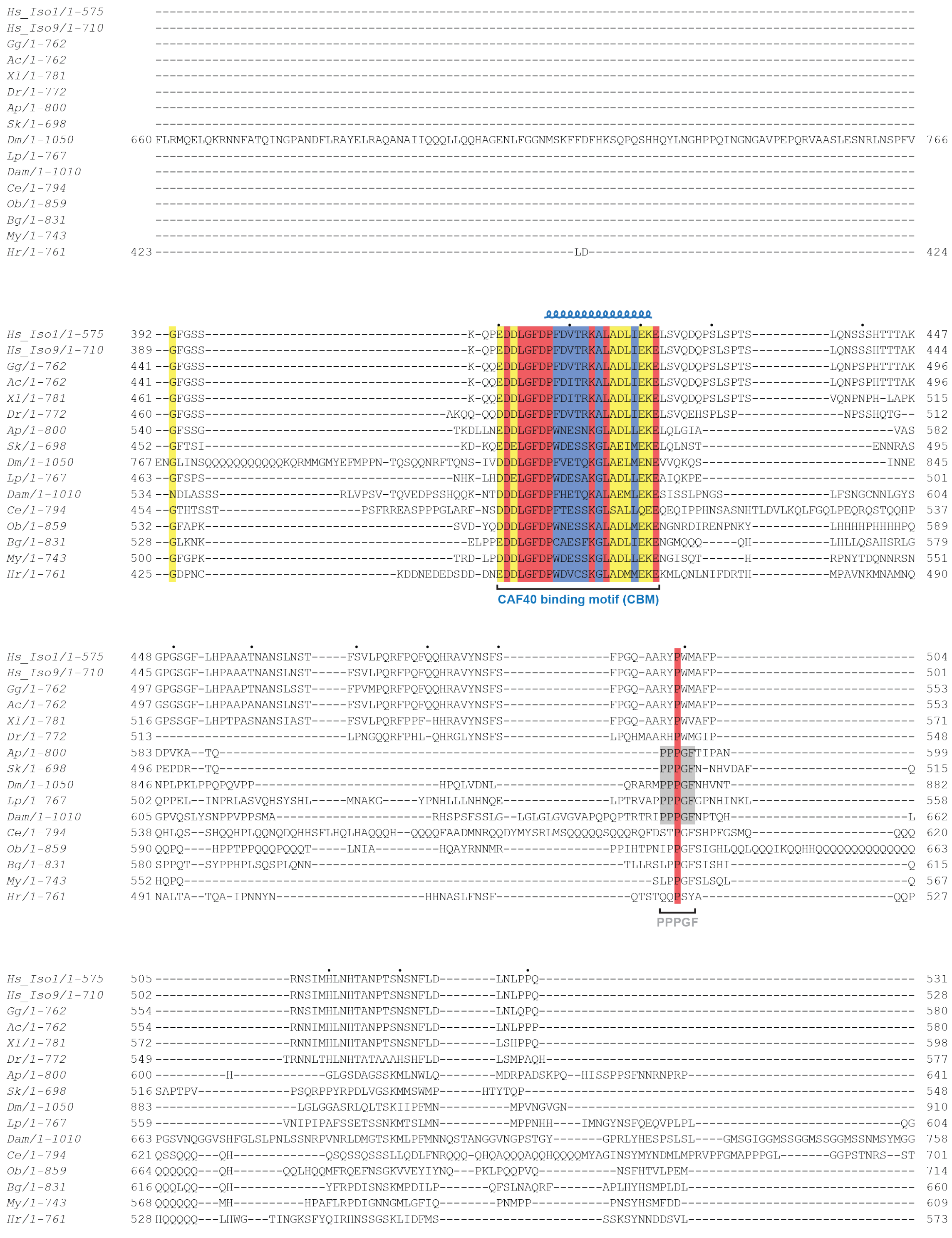

Supplemental Figure S4, continued. Sequence alignment of metazoan NOT4

```
Hs_Iso1/1-575     532 ------HNTGL----------GGIPVA------------GEEEVKVSTMPLSTSSHSLQQGQQPTSLHTTVA---------------------------------------  575
Hs_Iso9/1-710     529 ------HNTGL----------GGIPVADNSSSV--------------------------------------------ESLNMKEWQDGLRALLP-NININFGGLPN  573
Gg/1-762          581 ------HSTGL----------GGIPISDNSSSV--------------------------------------------ESLNMKEWQDGLRALLP-NINISFGGLPN  625
Ac/1-762          581 ------HGTGL----------GGIPIADNNSSV--------------------------------------------ESLNMKEWQDGLRALLP-NININFGGLPN  625
Xl/1-781          599 ------HNTGL----------GGITVADHSNSI--------------------------------------------EGLNMKEWQDGLKALLP-NININFGGLSN  643
Dr/1-772          578 ------HSTGL----------GGIPISENNGSV--------------------------------------------ESINVKEWQDGLRALLP-NININFGGLPN  622
Ap/1-800          642 ------HH-------------SPSQAQTKHPE-------------------------------------------------SVKSWQDGLRALLPNNINISFDVANP  680
Sk/1-698          549 ---------------------SNSVQHSSEPR-------------------------------------------------TVKDWQDGLRALLP-NINISFGQQGL  584
Dm/1-1050         911 ---------------------SGAQGQHQMPMGV------------------------------------------------------------NWNAPMGMHQN  934
Lp/1-767          605 LNNHIKHL-------------PVHSMQSLRPKIG-------------------------------------------NALNMKDWQESLRSMFP-NINISFGPPTT  653
Dam/1-1010        759 QNGSLSHMSNNKPQGYSTGMGSNMPYSNGSNSLGL-------------------------------------AASSIGD-TAPSMKDWQDGLRALFP-NVNISMGNGGA  828
Ce/1-794          702 TQQAPPHMTQQ----------SQQQQQQQQSSMGLFGMGG--------------------------------HQSMMQDHQSQQPQSAQDAFKALLP-NVNVRF-----  762
Ob/1-859          715 ------HLQ------------GKEQLMQNSSQH--------------------------------------------DGFHLKDLQDGLRALLP-NINISFGASPS  757
Bg/1-831          661 ------H----------------TPQHQHQPQR--------------------------------------------ELYNMKDMQEQLRSMLP-NINISFGSIPH  699
My/1-743          610 ---------------------GKEQNHVNAPR---------------------------------------------DVFNTKDWQDGLRALLP-NINISFGAA--  646
Hr/1-761          574 -----------------------------------------------------------------------NSECAVSQDWQDNLKALLP-NINISFSPSQT  603


Hs_Iso1/1-575         ------------------------------------------------------------------------------------------------------------
Hs_Iso9/1-710     574 SSSPSNANHSA--------PTSNTATTDS----------------------LSWDSPGS---------W---------------TDPAIITGIPASSGNSL-----  620
Gg/1-762          626 ASSPSNANHSV--------PTSNTATTDS----------------------LNWDSSGS---------W---------------MDPAIITGIPASTGNSL-----  672
Ac/1-762          626 ATSPSNANHSV--------PTSNTATTDS----------------------LNWDSPGS---------W---------------MDPAIITGIPASAGNSL-----  672
Xl/1-781          644 ASTPSNATHSA--------PTSNTATTNS----------------------LNWDSSGS---------W---------------MDPAIISGIPSSTGNGL-----  690
Dr/1-772          623 STSSSSSSSTSSVNHIG-VPIGSAGISHS----------------------LSWDSTAS---------W---------------MDPAIITGIPASTGNSL-----  676
Ap/1-800          681 AGNTARSATHKN---------------------------------------SDIWPGMQS-------QTWM-------------VHDPAIISVNSHSTHAPG-FTE-  726
Sk/1-698          585 SVPPQKDN------------------------------------------WNSASNQ------SNWP-------------TSDPAIITSITVSSAPSTSYTNA  626
Dm/1-1050         935 PGQPVGDSQLQH-------PMAHNKVYNN-------------------------------------SDWT-------------SMDPAILSFRQYSSFPQ------  977
Lp/1-767          654 QGLPPPNAPPNP----------------------KVH-------------HMWNPYG--------PSWV-------------TQDPAILSSGSIAD---------  693
Dam/1-1010        829 TNSNGNSSNHGG--RIGAFPPGLSGLGNSQQQTMHHQHHQHQLAQQPNNLATKGWRNNG--------SDWT-------------SLDPAIVSSGQITD---------  903
Ce/1-794              ------------------------------------------------------------------------------------------------------------
Ob/1-859          758 QPPPPQQQPSSS--------TPCTVQQSQ---------------------------------------------------NNP--------TQKSNF-----  787
Bg/1-831          700 QQQQQQQAQQHQLQQV---PTSLFSQQSQ--------H------------LNKSWPLSSSP----DSSPW---------------NDPAIVSTSQLTESGSL-----  759
My/1-743          647 -----QQANNAM--------SAHTSLQQQ-------------------------------------------------------------QKSSA-----  667
Hr/1-761          604 SAAGTLQQQQQQMRRNHVHAGGITIHPAQ----------------------NGFNSYHNLLHSQDNTDWLNSGGSMLNTNQSITSDPAIISTGVVTDGMSI-----  682


Hs_Iso1/1-575         ------------------------------------------------------------------------------------------------------------
Hs_Iso9/1-710     621 ----DSLQDDNPPHWLKS----LQALTEMDGP-SAAPSQTH-----------HSAPF------------STQIPLHR-ASWNPYPPPS--NPSSFHSPPPGFQTAFR  692
Gg/1-762          673 ----DTLQDDNPPHWLKS----LQALTEVDGP-SAAPSQTH-----------HSNPF------------GTQIPLHR-ASWNPYSPPS--NPTSFHSPPPGFQTAFR  744
Ac/1-762          673 ----DSLQDDNPPHWLKS----LQALTEMDGP-SAAPTQTP-----------HSNPF------------STQIPLHR-ASWNPYSPPS--NPASFHSPPPGFQTAFR  744
Xl/1-781          691 ----ESLQDDNPPHWLKS----LQALTEMDGP-SSSLLPHHP----------HNNLF------------SAQIPLHR-GNWSPYPPPS--NPASFHSPPPGFQTAFR  763
Dr/1-772          677 ----DCLQDDNPPHWLKS----LQALTEMDGPPSSSALPQPP----------HTGLL----------DAAAHLSLHR-AAWAPYLPPPTLTPNQFHSPPPGFQTAFR  754
Ap/1-800          727 ----KEVPTEEQPHWLKS----LQTLTDGDTPSHPSAN--------------HLFP-------------AHAYANRG-PAWTT------------HAPPPGLRPPTN  785
Sk/1-698          627 NSDRESNLSESPPHWMKS----LHTLTEVD-----SPT--------------HINNF------------YPAHIGRA-GSWFSQ-----------QPPPPGFPTSTA  686
Dm/1-1050         978 --------NQIPPHPQQQQDLFLQHLAQQQNSQSGGFN----------NQAQQMLPM-----------GMPNSLLNG-Q--------------QTQPPQVNANVQ 1038
Lp/1-767          694 ------CRSDSPPHWTKS----LHQLTTEGGT-MNNYN--------------HHMPF------------PMPFAYRA-TQWQGIP----------QSPPPGFSPPFQ  752
Dam/1-1010        904 ------SRSDSPPHWLRS----LEQLTETGNSPAQQPPSNPSNLFGLANSTHYTLPSLTGRSNVTPASGLDGLNSMG-SFWPPSV-----SHTTPSMPPPGF-SHIR  993
Ce/1-794          763 ---------------------------------------------------------------MDDNSM-SRWSHENS----LRSSAVPPPPGFSSVMN  793
Ob/1-859          788 ------SHMPDNPHWMKS----IQQLTEFD---SQGHT--------------HR-PF------------VQHFPVRG-TGWVPQT----------HNPPPGFHTQIR  843
Bg/1-831          760 ------FPETSAPHWLKS----LQHLTESDGP-------------------SHRLPF------------VQQFPLGGNGGWPPHT----------HNPPPGFRAAGL  815
My/1-743          668 ------LSSDSPPHWLKS----IHQLTDIEGSHTNSQN--------------HRLPF------------VQQFPLRN-TGWPTQT----------QTPPPGFQTSIR  727
Hr/1-761          683 ------------PRWMDQ----YSDLAADASCHIQHHN--------------HLQYRQ--QNNHYHNYGMHTEPQSS-SSWVQQH--------TSRYPPPGFASSRQ  748
                                                                                                                   PPPGF


Hs_Iso1/1-575         -----------------
Hs_Iso9/1-710     693 PPSKTPTDLLQSSTLDRH  710
Gg/1-762          745 PPSKTPTDLLQSSALDRH  762
Ac/1-762          745 PPSKTPTDLLQSSALDRH  762
Xl/1-781          764 PPTKTPTDLLQSSALDRH  781
Dr/1-772          755 PQAQTATDILQSAGIDRH  772
Ap/1-800          786 ST--DTHSHTLTENPAV-  800
Sk/1-698          687 GVHSPTEPQQSL------  698
Dm/1-1050        1039 GMLEFLKSRQFV------ 1050
Lp/1-767          753 PSLKSTEPQTLAEHL---  767
Dam/1-1010        994 PTPKTASTAETHKIDNL- 1010
Ce/1-794          794 R-----------------  794
Ob/1-859          844 PPTQTTEPHKMTEGLQ--  859
Bg/1-831          816 GSQANTDTHQMAEVLQ--  831
My/1-743          728 PPTHPTEPHKISEGLQ--  743
Hr/1-761          749 HT-EAASEHRLPAI----  761
```

Supplemental Figure S4. Sequence alignment of metazoan NOT4

Sequences cover the following phyla. Chordata: *Hs*, *Homo sapiens*; *Gg*, *Gallus gallus*; *Ac*, *Anolis carolinensis*; *Xl*, *Xenopus laevis*; *Dr*, *Danio rerio*. Echinodermata: *Ap*, *Acanthaster planci*. Hemichordata: *Sk*, *Saccoglossus kowalevskii*. Arthropoda: *Dm*, *Drosophila melanogaster*; *Lp*, *Limulus polyphemus*; *Dam*, *Daphnia magna*. Nematoda: *Ce*, *Caenorhabditis elegans*. Mollusca: *Ob*, *Octopus bimaculoides*; *Bg*, *Biomphalaria glabrata*; *My*, *Mizuhopecten yessoensis*. Annelida: *Hr*, *Helobdella robusta*. Uniprot or NCBI accession numbers are provided in the Supplemental alignment file SF1. Sequences were aligned using MAFFT (Katoh et al. 2002) as implemented in the MPI Bioinformatics Toolkit (Zimmermann et al. 2018). Structural domains in the conserved N-terminal region of NOT4 are marked below the alignment. Similarly, the presently identified CBM and PPPGΦ motifs are marked in the non-conserved C-terminal region of NOT4 and respectively shaded in blue and gray. Conserved residues are shaded in red, and residues with >70% similarity are shaded in yellow, with conservation scores calculated using the SCORECONS webserver (Valdar 2002). Secondary structure elements are indicated above the alignment and taken from the previously reported NMR structures of the RING and RRM domains (PDB-ID 1ur6, Dominguez et al. 2004, and PDB-ID 2cpi), from our presently determined crystal structure of the CBM, or from PSIPRED (http://bioinf.cs.ucl.ac.uk/psipred/) secondary structure prediction.

Supplemental Figure S5. Identification and analysis of the NOT4 CBM

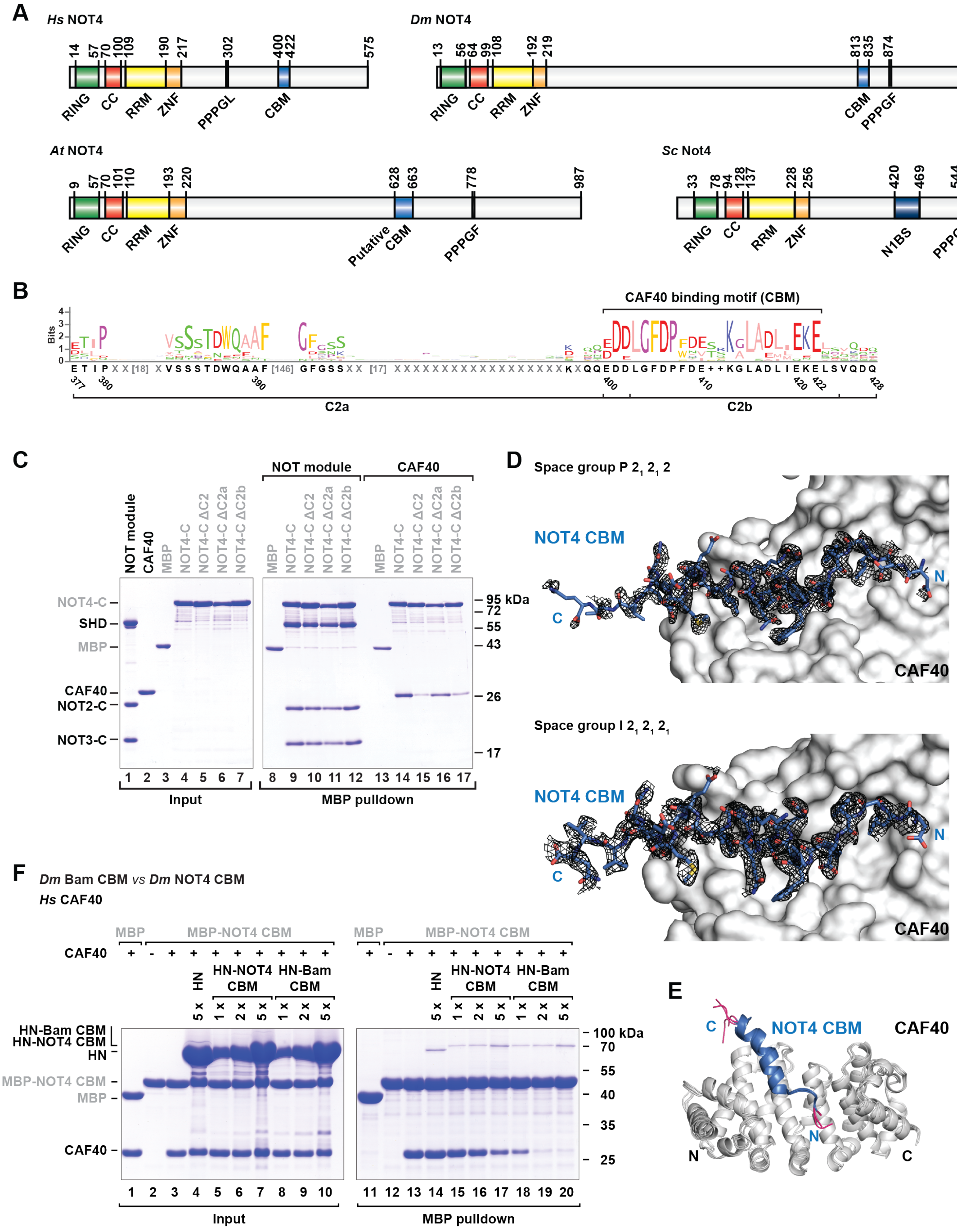

Supplemental Figure S5. Identification and analysis of the NOT4 CBM

(A) Domain composition of selected NOT4 proteins from *Homo sapiens*, *Drosophila melanogaster*, *Arabidopsis thaliana* and *Saccharomyces cerevisiae*, indicating the relative positions of conserved sequence motifs in NOT4-C. The sequences corresponding to the 23 amino acid motif are labelled 'CBM' or 'CBM-like' in *Arabidopsis thaliana*, and the sequences corresponding to the PPPGΦ motifs are listed as PPPGL, PPPGF or PPPGI. For alignments see Supplemental alignment files SF1–SF3.

(B) Sequence logo (Waterhouse et al. 2009) of the NOT4 C2 region as derived from the alignment in Supplemental Figure S4. The consensus sequence is indicated below the logo, along with residue numbers corresponding to the human sequence. The separately deleted C2a and C2b regions are indicated as well as the 23 amino acid motif which is labelled as 'CBM'.

(C) MBP pulldown assay with MBP-tagged deletion variants of *Hs* NOT4-C and purified recombinant NOT module or CAF40. Experiments were done as described in Figure 3A–D, identifying the 23 amino acid motif as a CAF40 binding motif (CBM). NOT4-C ΔC2 lacks residues E377–S424. NOT4-C ΔC2a lacks residues E377–D402. NOT4-ΔC2b lacks residues E400–Q428 including the CBM (E400–E422).

(D) Composite omit maps for the NOT4 CBM peptide. $2F_O$-F_C type electron density surrounding the CBM peptide is contoured at 1.0 σ. The maps were generated with phenix.composite_omit_map (Afonine et al. 2012) using the final refined models, respectively. Top panel: space group $P2_12_12$. Bottom panel: space group $I2_12_12_1$.

(E) Superposition of the four crystallographically independent complexes of CAF40 with the NOT4 CBM. The structurally variable and non-conserved flanks of the CBM are shown in magenta. The C-terminal flanks of the *Dm* NOT4 CBM (V836–Q838) mediate crystal contacts and differ from the *Hs* NOT4 CBM (L423–V425), possibly explaining why the *Hs* NOT4 CBM did not crystallize.

(F) Competition assay. *Hs* CAF40 was incubated with equimolar amounts of MBP-tagged *Dm* NOT4 CBM and increasing amounts of His_6-NusA-tagged *Dm* NOT4 CBM (HN-NOT4 CBM) or His_6-NusA-tagged *Dm* Bam CBM (HN-Bam CBM). His_6-NusA (HN) was used as a negative control. The amount of *Hs* CAF40 pulled down with the MBP-tagged *Dm* NOT4 CBM was analyzed by SDS-PAGE and subsequent Coomassie staining. Molar equivalents (1x, 2x, 5x) are relative to the MBP-tagged *Dm* NOT4 CBM. MBP-tagged constructs are labelled in gray. See Sgromo et al. (2018) for additional experimental details.

Supplemental Figure S6. Difference electron density for the CBM peptide

Space group P 2_1 2_1 2

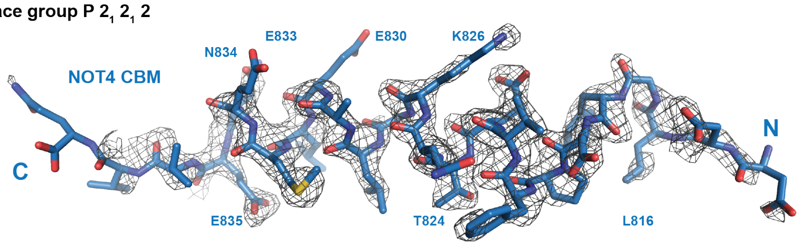

Space group I 2_1 2_1 2_1

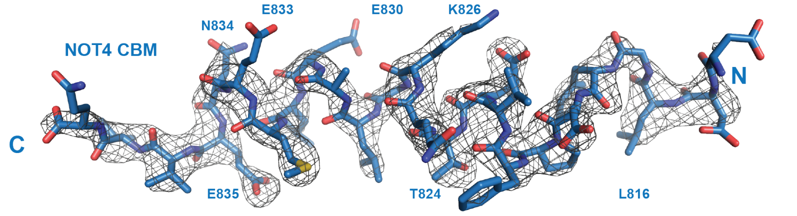

Supplemental Figure S6. Difference electron density for the CBM peptide

Classic F_O-F_C type difference electron density for the CBM peptide was calculated using the final refined models, respectively, but with the CBM peptide, ligands and water molecules omitted. The maps are contoured at 2.5 σ. The difference density is rather poor for the N-terminal and C-terminal residues of the modeled peptide and for the solvent-exposed side chains of K826, E830, E833 and N834. These residues were nevertheless modeled without truncating their side chains. The structurally conserved part of the CBM peptide as shown in Figure 4 ranges from L816 to E835. Top panel: space group $P2_12_12$. Bottom panel: space group $I2_12_12_1$.

Supplemental Figure S7. Importance of the NOT4 CBM and of the CCR4-NOT deadenylase for the degradation of tethered mRNA

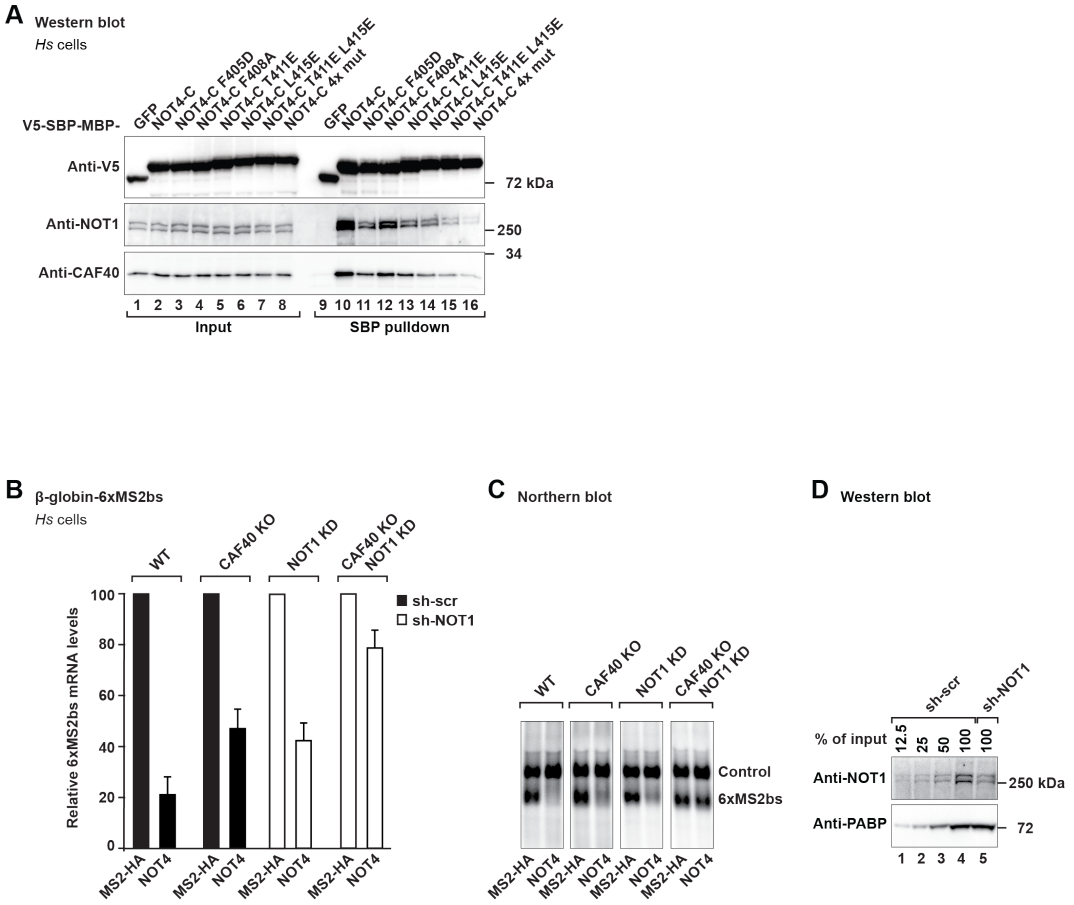

Supplemental Figure S7. Importance of the NOT4 CBM and of the CCR4-NOT deadenylase for the degradation of tethered mRNA

(A) SBP pulldown of endogenous *Hs* NOT1 and *Hs* CAF40 proteins with V5-SBP-MBP-tagged CBM mutation variants of *Hs* NOT4-C. The CBM contained single or multiple point mutations. 4x mut; F405D, F408A, T411E, L415E. For additional details see Figure 6E.

(B–D) Tethering assay with *Hs* NOT4 and the β-globin mRNA reporter in HEK293T cells lacking CAF40 (CAF40 KO) (Sgromo et al. 2018). Efficiency of CRISPR-Cas9-mediated gene editing of CAF40 is shown in Figure 7B. Experiments were done and analyzed as described in Figure 2A. (B) Relative mRNA levels with error bars corresponding to standard deviations (n=3). The shRNA-mediated depletion of the CCR4-NOT complex is indicated by white bars (sh-NOT1 RNA, NOT1 KD) as compared to black bars (sh-scr RNA, control). WT: wildtype, KO: knockout, KD: knockdown. (C) Northern blot. **(D)** Efficiency of shRNA-mediated depletion of NOT1. The Western blot shows HEK293T cells expressing sh-NOT1 RNA in comparison to a dilution series of HEK293T cells expressing sh-scr RNA using PABP as a loading control.

Supplemental Table S1. Generated plasmid constructs

Plasmids expressing *Hs* NOT4 protein and *Hs* NOT4 fragments (Uniprot-ID O95628-1)

Name	Construct details	Plasmid backbone *
Hs NOT4	MS2-HA-*Hs* NOT4	pcDNA3.1-MS2-HA
	V5-SBP-MBP-*Hs* NOT4	pCIneo-V5-SBP-MBP
	MBP-*Hs* NOT4-GB1-6xHis	pnEA-pM, GB1
Hs NOT4-N	MS2-HA-*Hs* NOT4_1–274	pcDNA3.1-MS2-HA
	V5-SBP-MBP-*Hs* NOT4_1–274	pCIneo-V5-SBP-MBP
	MBP-*Hs* NOT4_1–274-GB1-6xHis	pnEA-pM, GB1
Hs NOT4-C	MS2-HA-*Hs* NOT4_275–575	pcDNA3.1-MS2-HA
	V5-SBP-MBP-*Hs* NOT4_275–575	pCIneo-V5-SBP-MBP
	MBP-*Hs* NOT4_275–575-GB1-6xHis	pnEA-pM, GB1
Hs NOT4 ΔRING	V5-SBP-MBP-*Hs* NOT4 (Δ1–63)	pCIneo-V5-SBP-MBP
Hs NOT4 ΔCC	V5-SBP-MBP-*Hs* NOT4 (Δ64–104)	pCIneo-V5-SBP-MBP
Hs NOT4 ΔRRM	V5-SBP-MBP-*Hs* NOT4 (Δ109–189)	pCIneo-V5-SBP-MBP
Hs NOT4 ΔCC-RRM	V5-SBP-MBP-*Hs* NOT4 (Δ64–189)	pCIneo-V5-SBP-MBP
Hs NOT4 ΔZNF	V5-SBP-MBP-*Hs* NOT4 (Δ190–274)	pCIneo-V5-SBP-MBP
Hs NOT4-CC-C	V5-SBP-MBP-*Hs* NOT4_64–104_GSSG_275–575	pCIneo-V5-SBP-MBP
Hs NOT4-RRM-C	V5-SBP-MBP-*Hs* NOT4_109–189_GSSG_275–575	pCIneo-V5-SBP-MBP
Hs NOT4-ZNF-C	V5-SBP-MBP-*Hs* NOT4_190–575	pCIneo-V5-SBP-MBP
Hs NOT4-C1	MS2-HA-*Hs* NOT4_275–376	pcDNA3.1-MS2-HA
	MBP-*Hs* NOT4_275–376-GB1-6xHis	pnEA-pM, GB1
Hs NOT4-C2	MS2-HA-*Hs* NOT4_377–428	pcDNA3.1-MS2-HA
	MBP-*Hs* NOT4_377–428-GB1-6xHis	pnEA-pM, GB1
Hs NOT4-C3	MS2-HA-*Hs* NOT4_429–575	pcDNA3.1-MS2-HA
	MBP-*Hs* NOT4_429–575-GB1-6xHis	pnEA-pM, GB1
Hs NOT4 ΔC2	MS2-HA-*Hs* NOT4 (Δ377–424)	pcDNA3.1-MS2-HA
Hs NOT4-C ΔC2	V5-SBP-MBP-*Hs* NOT4_275–575 (Δ377–424)	pCIneo-V5-SBP-MBP
	MBP-*Hs* NOT4_275–575-GB1-6xHis (Δ377–424)	pnEA-pM, GB1
Hs NOT4 ΔC2a	MS2-HA-*Hs* NOT4 (Δ377–402)	pcDNA3.1-MS2-HA
Hs NOT4-C ΔC2a	V5-SBP-MBP-*Hs* NOT4_275–575 (Δ377–402)	pCIneo-V5-SBP-MBP
	MBP-*Hs* NOT4_275–575-GB1-6xHis (Δ377–402)	pnEA-pM, GB1
Hs NOT4 ΔC2b	MS2-HA-*Hs* NOT4 (Δ400–428)	pcDNA3.1-MS2-HA
Hs NOT4-C ΔC2b	V5-SBP-MBP-*Hs* NOT4_275–575 (Δ400–428)	pCIneo-V5-SBP-MBP
	MBP-*Hs* NOT4_275–575-GB1-6xHis (Δ400–428)	pnEA-pM, GB1
Hs NOT4-CBM	MBP-*Hs* NOT4_400–427	pnEA-pM

* Plasmid backbones are described in Jonas et al. (2013) for pcDNA3.1-MS2-HA and pCIneo-V5-SBP-MBP, in Diebold et al. (2011) for pnEA-pM and in Cheng and Patel (2004) for GB1.

Supplemental Table S1, continued. Generated plasmid constructs

Plasmids expressing *Hs* NOT4 point mutations (Uniprot-ID O95628-1)

Name	Construct details	Plasmid backbone *
Hs NOT4 4x mut	MS2-HA-*Hs* NOT4 (F405D, F408A, T411E, L415E)	pcDNA3.1-MS2-HA
Hs NOT4-C 4x mut	V5-SBP-MBP-*Hs* NOT4_275–575 (F405D, F408A, T411E, L415E)	pCIneo-V5-SBP-MBP
Hs NOT4-C 2x mut	V5-SBP-MBP-*Hs* NOT4_275–575 (T411E, L415E)	pCIneo-V5-SBP-MBP
Hs NOT4-C F405D	V5-SBP-MBP-*Hs* NOT4_275–575 (F405D)	pCIneo-V5-SBP-MBP
Hs NOT4-C F408A	V5-SBP-MBP-*Hs* NOT4_275–575 (F408A)	pCIneo-V5-SBP-MBP
Hs NOT4-C T411E	V5-SBP-MBP-*Hs* NOT4_275–575 (T411E)	pCIneo-V5-SBP-MBP
Hs NOT4-C L415E	V5-SBP-MBP-*Hs* NOT4_275–575 (L415E)	pCIneo-V5-SBP-MBP

* Plasmid backbones are described in Jonas et al. (2013).

Plasmids expressing *Dm* NOT4 proteins (Uniprot-ID M9PCL9)

Name	Construct details	Plasmid backbone *
Dm NOT4	λN-HA-*Dm* NOT4	pAc5.1B-λN-HA
Dm NOT4-N	λN-HA-*Dm* NOT4_1–249	pAc5.1B-λN-HA
Dm NOT4-C	λN-HA-*Dm* NOT4_248–1050	pAc5.1B-λN-HA
Dm NOT4-CBM	MBP-*Dm* NOT4_813–836	pnYC-vM
	His_6-NusA-*Dm* NOT4_813–836	pnYC-vHN
Dm NOT4-CBM F821D	MBP-*Dm* NOT4_813–836 (F821D)	pnYC-vM
Dm NOT4-CBM L828E	MBP-*Dm* NOT4_813–836 (L828E)	pnYC-vM

* Plasmid backbones are described in Rehwinkel et al. (2005) for pAc5.1B-λN-HA and in Diebold et al. (2011) for pnYC-vM.

Supplemental Table S2. Antibodies

**Antibody**	**Source**	**Catalog number**	**Dilution**	**Monoclonal/polyclonal**
Anti-NOT1	In house	-	1:1000	Rabbit polyclonal
Anti-NOT2	Bethyl	A302-562A	1:2000	Rabbit polyclonal
Anti-NOT3	Abcam	ab55681	1:2000	Mouse monoclonal
Anti-CAF40	Proteintech	22503-1-AP	1:1000	Rabbit polyclonal
Anti-EDC4	Santa Cruz Biotech	SC-8418	1:1000	Mouse monoclonal
Anti-PABP	Abcam	ab21060	1:10000	Rabbit polyclonal
Anti-tubulin	Sigma	T6199	1:5000	Mouse monoclonal
Anti-V5	LSBio	LS-C57305	1:5000	Mouse monoclonal
Anti-GFP	Roche	11 814 460 001	1:2000	Mouse monoclonal
Anti-HA-HRP	Roche	12 013 819 001	1:5000	Rat monoclonal

Supplemental alignment files

Supplemental alignment files contain CLUSTAL-formatted text files with sub-alignments of NOT4 proteins and their Uniprot or NCBI accession codes.

SF1: SF1_metazoan_NOT4_alg.rtf

SF2: SF2_plant_NOT4_alg.rtf

SF3: SF3_yeast_NOT4_alg.rtf

Supplemental references

Afonine PV, Grosse-Kunstleve RW, Echols N, Headd JJ, Moriarty NW, Mustyakimov M, Terwilliger TC, Urzhumtsev A, Zwart PH, Adams PD. 2012. Towards automated crystallographic structure refinement with phenix.refine. *Acta Crystallogr D Biol Crystallogr* **68**: 352-367.

Bhandari D, Raisch T, Weichenrieder O, Jonas S, Izaurralde E. 2014. Structural basis for the Nanos-mediated recruitment of the CCR4-NOT complex and translational repression. *Genes Dev* **28**: 888-901.

Cheng Y, Patel DJ. 2004. An efficient system for small protein expression and refolding. *Biochem Biophys Res Commun* **317**: 401-405.

Diebold ML, Fribourg S, Koch M, Metzger T, Romier C. 2011. Deciphering correct strategies for multiprotein complex assembly by co-expression: application to complexes as large as the histone octamer. *J Struct Biol* **175**: 178-188.

Dominguez C, Bonvin AM, Winkler GS, van Schaik FM, Timmers HT, Boelens R. 2004. Structural model of the UbcH5B/CNOT4 complex revealed by combining NMR, mutagenesis, and docking approaches. *Structure* **12**: 633-644.

Jonas S, Weichenrieder O, Izaurralde E. 2013. An unusual arrangement of two 14-3-3-like domains in the SMG5-SMG7 heterodimer is required for efficient nonsense-mediated mRNA decay. *Genes Dev* **27**: 211-225.

Katoh K, Misawa K, Kuma K, Miyata T. 2002. MAFFT: a novel method for rapid multiple sequence alignment based on fast Fourier transform. *Nucleic Acids Res* **30**: 3059-3066.

Kuzuoğlu-Öztürk D, Bhandari D, Huntzinger E, Fauser M, Helms S, Izaurralde E. 2016. miRISC and the CCR4-NOT complex silence mRNA targets independently of 43S ribosomal scanning. *EMBO J* **35**: 1186-1203.

Rehwinkel J, Behm-Ansmant I, Gatfield D, Izaurralde E. 2005. A crucial role for GW182 and the DCP1:DCP2 decapping complex in miRNA-mediated gene silencing. *RNA* **11**: 1640-1647.

Sgromo A, Raisch T, Backhaus C, Keskeny C, Alva V, Weichenrieder O, Izaurralde E. 2018. Drosophila Bag-of-marbles directly interacts with the CAF40 subunit of the CCR4-NOT complex to elicit repression of mRNA targets. *RNA* **24**: 381-395.

Valdar WS. 2002. Scoring residue conservation. *Proteins* **48**: 227-241.

Waterhouse AM, Procter JB, Martin DM, Clamp M, Barton GJ. 2009. Jalview Version 2--a multiple sequence alignment editor and analysis workbench. *Bioinformatics* **25**: 1189-1191.

Zimmermann L, Stephens A, Nam SZ, Rau D, Kübler J, Lozajic M, Gabler F, Söding J, Lupas AN, Alva V. 2018. A Completely Reimplemented MPI Bioinformatics Toolkit with a New HHpred Server at its Core. *J Mol Biol* **430**: 2237-2243.
