# Supplementary material for: A conserved CAF40-binding motif in metazoan NOT4 mediates association with the CCR4–NOT complex
Source: Genes Dev. 2019 Feb 1;33(3-4):236–52. doi: 10.1101/gad.320952.118 (PMC6362812; doi:10.1101/gad.320952.118)
Supplement: Supplemental Material [file supp_gad.320952.118_Supplemental_Alignment_File_SF3.zip › Supplemental_Alignment_FileSF3.rtf]

CLUSTAL W (1.7) multiple sequence alignment of yeast NOT4Saccharomyces_cerevisiae_sp|P34909|   MMNPHVQENLQAIHNALSNFDTSFLSEDEED-YCPLCIEPMDITDKNFFPCPCGYQICQFLachancea_thermotolerans_tr|C5DBQ4|   MNKTTTAANMHAIQNALANYDTSFLSDDEED-YCPLCIETLDITDKNFKPCPCGYQICQFCandida_albicans_tr|Q9P927|           M----------------IQADDTFISDDEEE-YCPLCVEEMDISDKNFKPCPCGYQICQFKomagataella_pastoris_tr|A0A1B2JGF5|  M----------------MNNDDTFISDQEED-VCPLCVEEMDISDKNFRPCPCGYQVCQFYarrowia_lipolytica_tr|Q6C372|        M----------------AFQADSFISDEEEE-VCPLCVEEMDISDRNFKPCPCGYQICQFSchizosaccharomyces_pombe_sp|Q09818|  M----------------LKTQEIYSSEDEDDMCCPLCMEEIDISDKNFKPCQCGYRVCRFSaccharomyces_cerevisiae_sp|P34909|   CYNNIRQNPELNGRCPACRRKYDDENVRYVTLSPEELKMERAKLARKEKERKHREKERKELachancea_thermotolerans_tr|C5DBQ4|   CYNNIRQNPELNGRCPACRRKYDDESVEYIVLTPEEMKLEQAKQVRKEKERKLRDKERKECandida_albicans_tr|Q9P927|           CYNNIRQNPELNGRCPGCRRLYDDESVEYKTVSAEEYKLMQLKKEKRDREKKQKEKEKKEKomagataella_pastoris_tr|A0A1B2JGF5|  CYNNIRQNPELNGKCPACRRPYEDKNVEYKVISQEEWKLNQAKQARKEKERKQREKEKKDYarrowia_lipolytica_tr|Q6C372|        CYNNIRQNPQLNGRCPGCRRPYDDESVEYKVISPEEWKKHHVKQTKQERERKQKEREKKESchizosaccharomyces_pombe_sp|Q09818|  CWHHIKE--DLNGRCPACRRLYTEENVQWRPVTAEEWKMDL----HRKNERKKREKERKESaccharomyces_cerevisiae_sp|P34909|   NEYTNRKHLSGTRVIQKNLVYVVGINPPVPYEEVAPTLKSEKYFGQYGKINKIVVNRKTPLachancea_thermotolerans_tr|C5DBQ4|   NEFANRKHLAGMRVIQKNLVYVVGLNPPVPYEDVAALLKSDRYFGQYGKINKIVVNKKTACandida_albicans_tr|Q9P927|           MEMVNKKHLAGLRVVQKNLVYVTGLNPPCNPDDLHSVLRSDKYFGQYGKISKIVINKKTPKomagataella_pastoris_tr|A0A1B2JGF5|  AEQASRRHLAGMRVIQKNLVYVVGLNPPVSPEELHNVLRSEKYFGQYGKILKIVINKRNRYarrowia_lipolytica_tr|Q6C372|        MEQSSRKHLSGMRVIQKNLVYVIGLNPDIPTEDLHNTLRGEQFFGQYGRIQKIVINRRN-Schizosaccharomyces_pombe_sp|Q09818|  VELSNRKHLANIRVVQKNLAYVNGLSPKVANEENINVLKGPEYFGQYGKIIKIAINKKAASaccharomyces_cerevisiae_sp|P34909|   HSNNTT--SEHYHHHSPGYGVYITFGSKDDAARCIAQVDGTYMDGRLIKAAYGTTKYCSSLachancea_thermotolerans_tr|C5DBQ4|   HNDHHSGPASGSHGHHTGYGIYVTFAKKDDAARCIAAVDGTYLDGRIVKAAYGTTKYCSSCandida_albicans_tr|Q9P927|           TTQT-----STHHHQNPGLVVYVTFTRKEDALRCITELDGSLCDGRVLRAAHGTTKYCSSKomagataella_pastoris_tr|A0A1B2JGF5|  ---------TNNHNHNPGFGVYVTFARKEDASRCIAAVDGSISDGRVLRAAHGTTKYCSSYarrowia_lipolytica_tr|Q6C372|        -----------NVNGTPGLGVYVTFSKKEDAARCIAAVDGSMNDGKYLRAAYGTTKYCSSSchizosaccharomyces_pombe_sp|Q09818|  ANSA-----------NGHVGVYITYQRKEDAARAIAAIDGSVSDGRHLRASYGTTKYCTSSaccharomyces_cerevisiae_sp|P34909|   YLRGLPCPNPNCMFLHEPGEEADSFNKRELHNKQQAQQQSG-------------------Lachancea_thermotolerans_tr|C5DBQ4|   YLRGQSCPNPNCMFLHEPGEEADSFNRRELTNKQHDHSAFK-----QLGGSSSSLPSNKACandida_albicans_tr|Q9P927|           YLRGQPCPNPNCMFLHEPGEEADSYTRKDLSTQQGIKMGMT-----ARSHTTS-------Komagataella_pastoris_tr|A0A1B2JGF5|  YLKGQNCPNPNCMFLHEPGEEADSYTRQDLSTRQGLKMGES-----PRAAVHGLIPPPDMYarrowia_lipolytica_tr|Q6C372|        YLRGQPCPNPNCMFLHEPGEEADSYTRQDLSTIQHAARQGIQKQQRQQQQQQRY------Schizosaccharomyces_pombe_sp|Q09818|  YLRNQQCPNPSCMYLHEPGDEVDSYTKEDLASLQHTRPLST-----KPNVVNGATHSPSPSaccharomyces_cerevisiae_sp|P34909|   GTAFTRS-GIHNNISTSTAGSNTNL--LSENFTGTPSP---AAMRAQLHH----------Lachancea_thermotolerans_tr|C5DBQ4|   ----------------------AASAIVPPSASPAPSP---LPIKTHLNSSHN-------Candida_albicans_tr|Q9P927|           ---------------------------FGEESSNNSQSN--QAGSDDDHH----------Komagataella_pastoris_tr|A0A1B2JGF5|  ----------------------------------VPSPSIEEPVATERH-----------Yarrowia_lipolytica_tr|Q6C372|        ---------------------------GDFQQHSQPSP---VQPNTPIRH----------Schizosaccharomyces_pombe_sp|Q09818|  SLPFKTPL-----LPVT----------KTPLEEANSSP---AAQNQHITTVDHVHPQVSMSaccharomyces_cerevisiae_sp|P34909|   --------------------D--------------SHTNAGTPVLTPAPVPAGSNPWGVTLachancea_thermotolerans_tr|C5DBQ4|   ------------------QEEIS---------SSSSTSAAHTPVLTPALVPSGSNPWGVNCandida_albicans_tr|Q9P927|           --------------------------------------EAHKEHL-PSTA-----HWAAAKomagataella_pastoris_tr|A0A1B2JGF5|  -----------------V-----------------DTQVSDGPIL-PSTA-----SWGKNYarrowia_lipolytica_tr|Q6C372|        ------------------QSGHA-------AVAQHATEDIHGNAL-PATA-----SWAKQSchizosaccharomyces_pombe_sp|Q09818|  TPSLSTNNTATSVPAPY---SSAASVNVVPGHATTILHHEESSAL-PPTA-----AWAKLSaccharomyces_cerevisiae_sp|P34909|   ----QSAT--PV---TSINLSKNSSSINLPTLNDSLGHHT---TPTTENTITSTTTTTNTLachancea_thermotolerans_tr|C5DBQ4|   ----SAAA--PS---FSSAAAKTTTSSAFPTLGEATLAQS---MAALSTK-------DSGCandida_albicans_tr|Q9P927|           GTTSGSNS--PS----VNNNIPLANSAAFPTLGEIVRDQKQ--QQKKETK----------Komagataella_pastoris_tr|A0A1B2JGF5|  --TKASSTILPTFHSPSVEHSTLKNASSFPSFAETQQLHA---NLHTPPA----------Yarrowia_lipolytica_tr|Q6C372|        ----SPTT--PA----GKLHTPAQASPVKPTPVEPAVNYPMPD-----------------Schizosaccharomyces_pombe_sp|Q09818|  ---------SPS--VLQERLRAAVNQQPLDALKSSSTQTS--------------------Saccharomyces_cerevisiae_sp|P34909|   NATS--HSHG-SKKKQSLAAEEYKDPYD-ALGNAVDFLDARLHSL-SNYQKRPISI-KSNLachancea_thermotolerans_tr|C5DBQ4|   AGT----AHSSTKSNKKNLEKKYIDPYD-PLSSAVRFIDDTIKFL-SEYRNCHFKL-RSNCandida_albicans_tr|Q9P927|           ----------PRSKTTKSNNTLVPDELDAGDNSVFKFVETTTEQL-RGLEDIR----NVRKomagataella_pastoris_tr|A0A1B2JGF5|  ----------PKKKDKKSDGHESLDEVSKEILSAVENIEETVKNF-FQTQNSGYHL-KDEYarrowia_lipolytica_tr|Q6C372|        -----------------------PDPKR-QMDFTLPLFKNTLKSL-SK-NKFDFVF-SSASchizosaccharomyces_pombe_sp|Q09818|  ----IP-------KIQKLKAAKLPS----EEENTTKWLNKAINDLVSSLSKINFSTEGTESaccharomyces_cerevisiae_sp|P34909|   I--IDE----ETYKKYPSLFS--WDKI-----EASKKSDNTLANKLVEILAIKPIDYTASLachancea_thermotolerans_tr|C5DBQ4|   V--IDE----ETYKSYPSLFS--FENV-----PVSKTSDNTLSRKLVDMLAIKPVDHSASCandida_albicans_tr|Q9P927|           FKGFDN-------GKILPLFS--FKGN--FSNDTQLEEEKVIARQVIEKFLLRPLKNYHLKomagataella_pastoris_tr|A0A1B2JGF5|  YK--------YRKRDIPRLFLSGFQQAPAFF-EESKDGNSEDMSHLVDLLLFSPGVKNYSYarrowia_lipolytica_tr|Q6C372|        I--LKSLDLDDDKNSLPNLFA--FSKD-NIMDPSKHESSEFLEGQFTPFAIFSGGKAAALSchizosaccharomyces_pombe_sp|Q09818|  FDKKQI----EMIQNLPPLFV--FNARSVIDKEVVPEQEK--------------------Saccharomyces_cerevisiae_sp|P34909|   VVQ---------FLQS-VNVG---VNDNITITDN------------TKTP---TQP----Lachancea_thermotolerans_tr|C5DBQ4|   VLP---------YLQA--TPQ---ADPLVL-----------------HQL---QQQ----Candida_albicans_tr|Q9P927|           AYQNHPITQQQAILQRQQQQQ---QQQQQQQ-----TPALQVAQ--LQQNKNDEQQ----Komagataella_pastoris_tr|A0A1B2JGF5|  FYRTTP-SQQQ--LQQQQQSQQAQQAQQAQQAQQVQQQAQQVQQAQQQQAQQAQQQQAQQYarrowia_lipolytica_tr|Q6C372|        PLASDE------------------------------------------------------Schizosaccharomyces_pombe_sp|Q09818|  ----------------SAENQ---------------PPTSLGIN----------------Saccharomyces_cerevisiae_sp|P34909|   IRLQTV------------------SQQIQPP-----------------------------Lachancea_thermotolerans_tr|C5DBQ4|   QQQQQLLLAK--------------QQQQLQQQ------------VQTHQQT---------Candida_albicans_tr|Q9P927|           KQAQQQLLLLQL------------QHQQQLQQ------------QQQQRQLQ--------Komagataella_pastoris_tr|A0A1B2JGF5|  QQQQQQLQQQHIQQLQQIQQLQLAQQQQQQQQQRQQQEHLLQQLQQQQQQYHQPQTQGQNYarrowia_lipolytica_tr|Q6C372|        -----------------------TQSFQQLMK----------------------------Schizosaccharomyces_pombe_sp|Q09818|  ------------------------------------------------------------Saccharomyces_cerevisiae_sp|P34909|   -------------------------------------------------LNV-STPPPGILachancea_thermotolerans_tr|C5DBQ4|   --------QQPTPPGMTP-QLLHAQLQ------------------QQ--RAVNTPPPPGICandida_albicans_tr|Q9P927|           ----------------QQQN-QPHQLNILK--------------TSDR-VNTSTPPPPGLKomagataella_pastoris_tr|A0A1B2JGF5|  FPQQYLMQ---------QAQ-AQAQAQVLAHAQHDSPSNPMFTNIREGSGVSATPPPPGLYarrowia_lipolytica_tr|Q6C372|        ----------GGPTDKVPEQ--------------------------------AATPPPGLSchizosaccharomyces_pombe_sp|Q09818|  -------------------------------------------------NGNPVMPPPGFSaccharomyces_cerevisiae_sp|P34909|   FGPQHKVPIQQQ--QMGDTSSRNSSDLLNQLINGRKIIAGN-Lachancea_thermotolerans_tr|C5DBQ4|   FSPSN-PPTSA---NQVPVTGSNSSDLLNQLINGKRISTSS-Candida_albicans_tr|Q9P927|           FAGKD-VAASSDMSNTGAPLPSSSSQLLTQLMSGKR------Komagataella_pastoris_tr|A0A1B2JGF5|  FSGVTN--------HNTETHNTPSSELLNQLMNGEGRNSINAYarrowia_lipolytica_tr|Q6C372|        MGDSNQ-------------KQHHSQELLAHLMNGGKKGEN--Schizosaccharomyces_pombe_sp|Q09818|  QS----------------------------------------
