# Supplementary material for: A conserved CAF40-binding motif in metazoan NOT4 mediates association with the CCR4–NOT complex
Source: Genes Dev. 2019 Feb 1;33(3-4):236–52. doi: 10.1101/gad.320952.118 (PMC6362812; doi:10.1101/gad.320952.118)
Supplement: Supplemental Material [file supp_gad.320952.118_Supplemental_Alignment_File_SF1.zip › Supplemental_Alignment_File_SF1.rtf]

CLUSTAL W (1.7) multiple sequence alignment of metazoan NOT4Homo_sapiens_sp|O95628|                  -----MSRSPDAKEDPVECPLCMEPLEIDDINFFPCTCGYQICRFCWHRIRTDENGLCPAHomo_sapiens_sp|O95628-9|                -----MSRSPDAKEDPVECPLCMEPLEIDDINFFPCTCGYQICRFCWHRIRTDENGLCPAGallus_gallus_tr|Q5ZJC9|                 -----MSRSPDAKEDPVECPLCMEPLEIDDINFFPCTCGYQICRFCWHRIRTDENGLCPAAnolis_carolinensis_tr|G1KKZ3|           -----MSRSPDVKEDPVECPLCMEPLEIDDINFFPCTCGYQICRFCWHRIRTDENGLCPAXenopus_laevis_XP_018109568.1            -----MSRSPVLKEDPVECPLCMEPLEIDDINFFPCTCGYQICRFCWHRIRTDENGLCPADanio_rerio_tr|E7F2Q8|                   -----MSRSPELKEDPMECPLCMEPLEIDDVNFFPCTCGYQICRFCWHRIRTDENGLCPAAcanthaster_planci_XP_022096362.1        MKPEAAGNNVNNIHEPDDCPLCMEPLEIDDINFFPCTCGYQICRFCWHRIRTDENGLCPASaccoglossus_kowalevskii_XP_006816490.1  ------MANNNNHEDPMECPLCMEALEMDDVNFFPCTCGYQICRFCWHRIRTDENGLCPADrosophila_melanogaster_tr|M9PCL9|       ------MNGLSSNDDAVECPLCMEPLEVDDLTFFPCTCGYQICRFCWHRIRTDENKLCPALimulus_polyphemus_XP_013776233.1        --------MSSFEDQNPECPLCMEPLELDDINFFPCTCGYQICRFCWHRIRTDENGLCPADaphnia_magna_tr|A0A0P5C974|             -----MSSLNHSGEESSECPLCMEPLEMDDLSFYPCTCGYQICRFCWHRIRTDENGLCPACaenorhabditis_elegans_tr|W6RRU8|        ------MSTCSDESCDKECPLCMETLELDDINFYPCKCEYQICRFCWHRIRTDENGLCPAOctopus_bimaculoides_tr|A0A0L8FVC4|      ----------MSGEDQIECPLCMDPLEIDDKNFFPCTCGYQICRFCWHRIRTDENGLCPABiomphalaria_glabrata_tr|A0A2C9JPB9|     -----------MSEEPAECPLCMEALEIDDHSFFPCTCGYQICRFCWHRIRTDENGLCPAMizuhopecten_yessoensis_tr|A0A210Q4R3|   ----------MSGEEQIECPLCMEPLEIDDINFFPCTCGYQICRFCWHRIRTDENGLCPAHelobdella_robusta_tr|T1EF86|            -------MTTNMQEELPECPLCMEAFEVDDLSFIPCTCGYQICRFCWHRIRTNESGLCPAHomo_sapiens_sp|O95628|                  CRKPYPEDPAVYKPLSQEELQRIKNEKKQKQNERKQKISENRKHLASVRVVQKNLVFVVGHomo_sapiens_sp|O95628-9|                CRKPYPEDPAVYKPLSQEELQRIKNEKKQKQNERKQKISENRKHLASVRVVQKNLVFVVGGallus_gallus_tr|Q5ZJC9|                 CRKPYPEDPAVYKPLSQEELQRIKNEKKQKQNERKQKISENRKHLASVRVVQKNLVFVVGAnolis_carolinensis_tr|G1KKZ3|           CRKPYPEDPAVYKPLSQEELQRIKNEKKQKQNERKQKISENRKHLASVRVVQKNLVFVVGXenopus_laevis_XP_018109568.1            CRKPYPEDPAVYKPLSQEELQRIKNEKKQKQNERKQKISENRKHLASVRVVQKNLVFVVGDanio_rerio_tr|E7F2Q8|                   CRKPYPEDPAVYKPLSQEEIQRIKNEKKQKLNEKKQKVTENRKHLASVRVVQRNLVFVVGAcanthaster_planci_XP_022096362.1        CRKAYPECPADFKPLTEEELQRIKNEKRQKDHQRKQKITDSRKHLASVRVVQRNLVFVVGSaccoglossus_kowalevskii_XP_006816490.1  CRKPYPEHPADFKPLSEEELQKIKNEKKQKDMQRKQKISENRKHLASVRVVQKNLVFVVGDrosophila_melanogaster_tr|M9PCL9|       CRKEYPENPADFKPLSQEEMIAFKSQKRQRDQQRKQKITENRKHLANVRVVQKNLVFVVGLimulus_polyphemus_XP_013776233.1        CRKQYPEDPADFKPLTQEELQKIKNEKKQKDLQRKQKISENRKHLANLRVVQKNLVFVVGDaphnia_magna_tr|A0A0P5C974|             CRKAYPENPADFKPLSTQEMHRIKAEKRQKDQQKKQRATENRKHLANVRVVQRNLVFVVGCaenorhabditis_elegans_tr|W6RRU8|        CRQPYPEDPVNFKPMTTDDVRKHKDEQRLKKQAEKLKLSDARQYLCNYRVLQKNLVYVVGOctopus_bimaculoides_tr|A0A0L8FVC4|      CRKQYPEDPAEFKPLTEDELHRIKKEKRQKDLQRKQKAAENRKHLANVRVVQKNLVFVVGBiomphalaria_glabrata_tr|A0A2C9JPB9|     CRKQYPEDPAEFKPLTEDELQNIKKERKQKDVQRKQKAAENRKHLANVRVVQRNLVFVVGMizuhopecten_yessoensis_tr|A0A210Q4R3|   CRKQYPEDPAVFRPLTDDELQRIKKERKQKDLQRKQKAAENRKHLANVRVVQRNLVFVVGHelobdella_robusta_tr|T1EF86|            CRKPYPEDPAQFKPLTEDEIHNIKRDRRQKESQRKQKSAENRKHLANVRVVQKNLVFVVGHomo_sapiens_sp|O95628|                  LSQRLADPEVLKRPEYFGKFGKIHKVVINNSTSYAGSQ---GPSASAYVTYIRSEDALRAHomo_sapiens_sp|O95628-9|                LSQRLADPEVLKRPEYFGKFGKIHKVVINNSTSYAGSQ---GPSASAYVTYIRSEDALRAGallus_gallus_tr|Q5ZJC9|                 LSQRLADPEVLKRPEYFGKFGKIHKVVINNSTSYAGSQ---GPSASAYVTYIRSEDALRAAnolis_carolinensis_tr|G1KKZ3|           LSQRLADPEVLKRPEYFGKFGKIHKVVINNSTSYAGSQ---GPSASAYVTYIRSEDALRAXenopus_laevis_XP_018109568.1            LSQRLADPEVLKRPEYFGKFGKIHKVVINNSTSYAGSQ---GPSASAYVTYIRSEDALRADanio_rerio_tr|E7F2Q8|                   LSQRLADAEVLKRPEYFGKFGKIHKVVINNSTSYAGSQ---GPSASAYVTYIRSEDALRAAcanthaster_planci_XP_022096362.1        LSQRLADTEVLKKQEYFGRFGKIVKVVINQNTSYAGSQ---GPSASAYVTYYKYEDALKASaccoglossus_kowalevskii_XP_006816490.1  LSQRLADPEILKKHEYFGKFGKIVKVVINQSTSYAGSQ---GPSASAYVTYQKSEDALKADrosophila_melanogaster_tr|M9PCL9|       LPPRLADADILKKHEYFGKYGKIHKVVINPSTTYAGVQVRVGPSASAYVTYVNNSDALRALimulus_polyphemus_XP_013776233.1        LPPRLAEAEVLKKHDYFGKFGKIHKVVVNHSTSYAGSQ---GPSASAYVTYYRAEDALRADaphnia_magna_tr|A0A0P5C974|             LSPRLADPEVLKRHEYFGKLGKIHKVVINHSTQYAGSQ---GPSASAYVTYIRGEDALRACaenorhabditis_elegans_tr|W6RRU8|        LSPRVADPEILKKNEYFGRYGKIQKIVTSATPSLPAPH--LPPSHTAYVTYKRVDDALRAOctopus_bimaculoides_tr|A0A0L8FVC4|      LSQRLAEPDVLKKHEYFGKFGKIHKVVINQSTSYAGAQ---GPSASAYVTYNRSEDALKABiomphalaria_glabrata_tr|A0A2C9JPB9|     LSPRLADPEVLKKHEYFGKFGKIHKVVINQSTSYAGSQ---GPSASAYVTYVKPEDALRAMizuhopecten_yessoensis_tr|A0A210Q4R3|   LSKKLAEPEVLKKHEYFGKFGKIHKVVINQSTSYAGAQ---GPSASAYVTYHRSDDALRAHelobdella_robusta_tr|T1EF86|            LSQRLADAEVLKRHEYFGKFGKIHKVVINQSTSYIGSQ---GPSASAYVTYLRPEDALHAHomo_sapiens_sp|O95628|                  IQCVNNVVVDGRTLKASLGTTKYCSYFLKNMQCPKPDCMYLHELGDEAASFTKEEMQAGKHomo_sapiens_sp|O95628-9|                IQCVNNVVVDGRTLKASLGTTKYCSYFLKNMQCPKPDCMYLHELGDEAASFTKEEMQAGKGallus_gallus_tr|Q5ZJC9|                 IQCVNNVVVDGRTLKASLGTTKYCSYFLKNMQCPKPDCMYLHELGDEAASFTKEEMQAGKAnolis_carolinensis_tr|G1KKZ3|           IQCVNNVVVDGRTLKASLGTTKYCSYFLKNMQCPKPDCMYLHELGDEAASFTKEEMQAGKXenopus_laevis_XP_018109568.1            IQCVNNVVVDGRTLKASLGTTKYCSYFLKNMQCPKPDCMYLHELGDEAASFTKEEMQAGKDanio_rerio_tr|E7F2Q8|                   IQCVNNVIVDGRTLKASLGTTKYCSYFLKSMQCPKPDCMYLHELGDEAASFTKEEMQAGKAcanthaster_planci_XP_022096362.1        IQAVNNVHVDGRTLKASLGTTKYCSHFLKNSQCPKPDCMYLHELGDSAASFTKEDMQAGKSaccoglossus_kowalevskii_XP_006816490.1  IQAVNNVFVDGRTLKASLGTTKYCSHFLKNAQCPKPDCMYLHELGDEAASFTKEDMQLGKDrosophila_melanogaster_tr|M9PCL9|       IQSVNNIMIDGRLIKTSLGTTKYCSHFMKNQQCPKGDCMYLHELGDPEASFTKEEMHQGKLimulus_polyphemus_XP_013776233.1        IQAVNNIHVDGRTLKVSLGTTKYCSHFLKNQQCPKADCMYLHELGDEAASFTKEEMQQGKDaphnia_magna_tr|A0A0P5C974|             IQSVNNITVDGRTLRASLGTTKYCSHFMKNQVCPKPDCMYLHEIGDDAASFTKEEMQQGKCaenorhabditis_elegans_tr|W6RRU8|        IQGVHNSMLDGRLVKASLGTTKYCSSFLNSRKCFKPECMYLHENAEAEISFTKDDMHLGKOctopus_bimaculoides_tr|A0A0L8FVC4|      ILAVNNVHVDGRTLKASLGTTKYCSHFLKGAQCPKSDCMYLHELGEEAASFTKEEMQMGKBiomphalaria_glabrata_tr|A0A2C9JPB9|     ILTVNNVHVEGRTLKTSLGTTKYCSHFLKGSHCQKNDCMYLHELGEEAASFTKDEMQAGKMizuhopecten_yessoensis_tr|A0A210Q4R3|   ILAVNNVHVDGRTLKASLGTTKYCSHFLRGAQCPKQDCMYLHELGEEAASFTKEEMQVGKHelobdella_robusta_tr|T1EF86|            ILVVNNLQVDNRTLKASLGTTKYCSHFLKGTQCTKTDCMYLHDLGDEAASFSKEDIQQGKHomo_sapiens_sp|O95628|                  HQEYEQKLLQELY-----------------------------------------------Homo_sapiens_sp|O95628-9|                HQEYEQKLLQELY-----------------------------------------------Gallus_gallus_tr|Q5ZJC9|                 HQEYEQKLLQELY-----------------------------------------------Anolis_carolinensis_tr|G1KKZ3|           HQEYEQKLLQELY-----------------------------------------------Xenopus_laevis_XP_018109568.1            HQEYEQKLLQELY-----------------------------------------------Danio_rerio_tr|E7F2Q8|                   HQEYEQKLLQDLY-----------------------------------------------Acanthaster_planci_XP_022096362.1        HQEYEQKLAQEVLGGGTGQSQSAHSTAPSTQHQHQHQSQQPQQQQHQQQQSQQQSKRQTESaccoglossus_kowalevskii_XP_006816490.1  HQDYERKLLEQLF-----------------------------------------------Drosophila_melanogaster_tr|M9PCL9|       HLEYEKRLHDTLIA----------------------------------------------Limulus_polyphemus_XP_013776233.1        HQEYERILHEQVLGSCS-------------------------------------------Daphnia_magna_tr|A0A0P5C974|             HTDYERLLHEQLL-----------------------------------------------Caenorhabditis_elegans_tr|W6RRU8|        HTEYEKRLIESM------------------------NSRPPPPQST--LAS----QLDKIOctopus_bimaculoides_tr|A0A0L8FVC4|      HQEYEQKLLEQFM-----------------------NSQNLANNHV--TSSKTITSRKKSBiomphalaria_glabrata_tr|A0A2C9JPB9|     HQEYEQMLIEQFL-----------------------NAQNSANNHH--IANN---KIKKSMizuhopecten_yessoensis_tr|A0A210Q4R3|   HQEYEQKLLETFM-----------------------NSQNTANNHVTGTSNKTIPTRKKAHelobdella_robusta_tr|T1EF86|            HLQYEQKLIEQYS-------------------------QNPPATESIRISN----SKSKSHomo_sapiens_sp|O95628|                  -----------------KLNPNFLQLS----TGSVDKNKNKVTP-----LQRYDT-----Homo_sapiens_sp|O95628-9|                -----------------KLNPNFLQLS----TGSVDKNKNKVTP-----LQ---S-----Gallus_gallus_tr|Q5ZJC9|                 -----------------KLNPNFLQLS----TGTVDKNKNKVTA-----LQR---PNSNNAnolis_carolinensis_tr|G1KKZ3|           -----------------KLNPNFLQLS----TNAVDKNKNKVTS-----LQR---PNSNNXenopus_laevis_XP_018109568.1            -----------------KLNPNFLQLS----TGTVDKNKNKVTA-----LQR---PNSNNDanio_rerio_tr|E7F2Q8|                   -----------------KANPTFL-LT----STCGEKSKSKSNS-----TQR---PNSTNAcanthaster_planci_XP_022096362.1        TPPLPQQQ---QPKQKSGGGSGSSGNE----HSKKHSKSSRKKP----SASP---ATSQNSaccoglossus_kowalevskii_XP_006816490.1  -----------------GGGVTSTNDG----HTAPRLNTTRKAP-----SPP---IANTNDrosophila_melanogaster_tr|M9PCL9|       -----------------SLGPNAT----VAIPSSSSASSSSSGSGTNGSSAS---GNAQQLimulus_polyphemus_XP_013776233.1        -------------------------------HLSRRLASSPTNN-----TSH---GQTGHDaphnia_magna_tr|A0A0P5C974|             -----------SPCLN---------------HKEGRRKASASPPLKEGASNA---TQSGPCaenorhabditis_elegans_tr|W6RRU8|        LAPTSN-----SPRRYLEDDSDTVDDV---ERTGSAIHMCADDP----DDDD---ADSTNOctopus_bimaculoides_tr|A0A0L8FVC4|      SSPVTVEQS--SPPQQSTLLQQTTELQFLSTGSSSSSSSSSSSS-----SSS---ASSTSBiomphalaria_glabrata_tr|A0A2C9JPB9|     VSPQAIENAEVIPSLM-----DMTGDI---KHTLSNAGPLLPNPVHNHTGGG---AANGHMizuhopecten_yessoensis_tr|A0A210Q4R3|   TSPIPVNQ---TPTSGTPPQQQVSTSE----QIQQQQTPPNNTPPQ--HTQP---IPNTSHelobdella_robusta_tr|T1EF86|            PSPSSSDQT--SPSRLTIFSPDVADLSLIHKSSSCTETPDTNTT-----TSP---LTTTSHomo_sapiens_sp|O95628|                  ------------------------------------------------------------Homo_sapiens_sp|O95628-9|                ------------------------------------------------------------Gallus_gallus_tr|Q5ZJC9|                 ---------------------KDAWPSLQSSSKSA---NGL-------------------Anolis_carolinensis_tr|G1KKZ3|           ---------------------KDAWPSLLSSSKSV---NGL-------------------Xenopus_laevis_XP_018109568.1            ---------------------KDAWPSLQNSNRSA---NGL-------------------Danio_rerio_tr|E7F2Q8|                   ---------------------KEGWPSLQNYGKMV---NGL-------------------Acanthaster_planci_XP_022096362.1        --------------NSPPANNTDPWPVTNHGDPWP-TTN---------------------Saccoglossus_kowalevskii_XP_006816490.1  ---------------------KEAWPSLQKSTETN-------------------------Drosophila_melanogaster_tr|M9PCL9|       ---------------------KEAWPSLSVSP-----INGKEAAAT--------------Limulus_polyphemus_XP_013776233.1        ---------------------KEGWSTLQHKTDHR--TNGLTKGAVL-------------Daphnia_magna_tr|A0A0P5C974|             ---------------------KDGWPSLPPGCSTA---NLPS-------------LSTSVCaenorhabditis_elegans_tr|W6RRU8|        ------------------------------------------------------------Octopus_bimaculoides_tr|A0A0L8FVC4|      SSLVVGSGLAASQQQAALNASKDVWPVLNSGGGTS---NATT-------------VGTTIBiomphalaria_glabrata_tr|A0A2C9JPB9|     ---------------AHFNNSNSVWPVLDGASSPA-HVNGTKNDHTSNNVSRAQGVGSKLMizuhopecten_yessoensis_tr|A0A210Q4R3|   --------------------NKEAWPSLQTGRSEQRLTNG--------------------Helobdella_robusta_tr|T1EF86|            TT---ATSTTASSTTSNVN-----------------------------------------Homo_sapiens_sp|O95628|                  ------------------------------------------------------------Homo_sapiens_sp|O95628-9|                ------------------------------------------------------------Gallus_gallus_tr|Q5ZJC9|                 ---------------------------TMEHRKTPPILENGTD-------SEHMTPDGADAnolis_carolinensis_tr|G1KKZ3|           ---------------------------TMEHRKTPPILENGTD-------SEHMTPDGPDXenopus_laevis_XP_018109568.1            -----------------------------EHRKSPPILDNGLD-------PDHMTPDGPDDanio_rerio_tr|E7F2Q8|                   ---------------------------TTEHRKSPPLLDCLTD-------SDHMTPDEPDAcanthaster_planci_XP_022096362.1        -------------SSDTWPALAGQAKSSEPYSQSPEDTPPLV------------------Saccoglossus_kowalevskii_XP_006816490.1  --------------------------------------------------KQPPSPPHPQDrosophila_melanogaster_tr|M9PCL9|       ------------ATSSSGKSKREKLRNEKRHEKNKAKNKNGSN------TNANASNKENYLimulus_polyphemus_XP_013776233.1        -----SPQGGG-----QKSLGGGR-QSSKKHDSGEE--------------GRKGKGRERSDaphnia_magna_tr|A0A0P5C974|             --KVVVPSVKSEPPLTKEKSGKIKAKPTQQPTVHLP--------------VAVVQPEEVTCaenorhabditis_elegans_tr|W6RRU8|        ------------------------------------------------------------Octopus_bimaculoides_tr|A0A0L8FVC4|      VGSNVTPVGSK-ATSAERILNGNKVPNTTRHHTHRPSTPTKQNKCL----RDKRDPDKLSBiomphalaria_glabrata_tr|A0A2C9JPB9|     QHRTNTPPGSGGGSSASRAVGNGRQRGSNSLNSSEENHIQNQI-NVGSGNRSPTSTPPGDMizuhopecten_yessoensis_tr|A0A210Q4R3|   --------------------------NRPNHNQQKPDTQPLPNHNRAPGNHKARTKTEADHelobdella_robusta_tr|T1EF86|            ---------------------------AHVHNGLLDNIDYIENSNR------------IQHomo_sapiens_sp|O95628|                  --------------------------PIDK----------------PSDSLSIGNG----Homo_sapiens_sp|O95628-9|                --------------------------PIDK----------------PSDSLSIGNG----Gallus_gallus_tr|Q5ZJC9|                 SDFG----------------------PIDK----------------PSDSLSIGNG----Anolis_carolinensis_tr|G1KKZ3|           SDFG----------------------PVDK----------------PSDSLSIGNC----Xenopus_laevis_XP_018109568.1            SDFGLFWESAEHNVAKFGRGIEDDTSSIDK----------------SSESLSIGNG----Danio_rerio_tr|E7F2Q8|                   LEQGT---EQNTGLPPFPSALE-PTSPIDK----------------PSEPISIGNG----Acanthaster_planci_XP_022096362.1        -----------------------SNHTPETLGQ------------SPPTGISIQTK----Saccoglossus_kowalevskii_XP_006816490.1  LPQEM--------SPTL------VPNDIDD----------------INHTVTVGSPPCNHDrosophila_melanogaster_tr|M9PCL9|       VPETR------------------SSTSTETFAEATADAPASTKA-EPPQASSNRTRADRGLimulus_polyphemus_XP_013776233.1        LEESL------------------SRGSSSH------------------------------Daphnia_magna_tr|A0A0P5C974|             LPQAK------------------ANGSNRKTKEKTVVTAVLTAVLNPTAPVAAQPT----Caenorhabditis_elegans_tr|W6RRU8|        -----------------------TSNSIDD----------------PAPSNPADETVNSROctopus_bimaculoides_tr|A0A0L8FVC4|      MVENY------------------SNGSIDS----------------PNIRVSTSSSY---Biomphalaria_glabrata_tr|A0A2C9JPB9|     VEKNS------------------PNDQVNH---RGLP--------LPTPSTSQPVSP---Mizuhopecten_yessoensis_tr|A0A210Q4R3|   IENNA------------------LNNTIDK----------------PTDTSAKSDIP---Helobdella_robusta_tr|T1EF86|            LLDDL------------------ANSTTST----------------STTASSIVET----Homo_sapiens_sp|O95628|                  -----DNSQQISNSDTPSPPPGLSKS--------------NPVIPISSSNHSAR------Homo_sapiens_sp|O95628-9|                -----DNSQQISNSDTPSPPPGLSKS--------------NPVIPISSSNHSAR------Gallus_gallus_tr|Q5ZJC9|                 -----DSSQQITNSDTPSPPPGLTKP--------------NPVIPISSSNHSAR------Anolis_carolinensis_tr|G1KKZ3|           -----DNSQQISNSDTPSPPPGLSKS--------------NSVIPISSSNHSAR------Xenopus_laevis_XP_018109568.1            -----DNLQQILTSDTPSPPPGLSKP--------------NPSAPISSANHSAR------Danio_rerio_tr|E7F2Q8|                   -----ENISQTSSSDSPSPPPGLTKP--------------SLVVPISVAELTAR------Acanthaster_planci_XP_022096362.1        -GVRQPSTEGLPPPSPEQSQPVPTGS--------------------TSSQSNQK------Saccoglossus_kowalevskii_XP_006816490.1  QLPSPRGDLHITTNGIATTNGIRTTNGILTTNDTLLTTQIPPTVEPLARSIEQRI-----Drosophila_melanogaster_tr|M9PCL9|       KDRTTASAKEQKKSKEAAPAPAASKPA-------ERVETSESTIRQKKAEVTESCEDNLPLimulus_polyphemus_XP_013776233.1        --SNSSSRESLHTFRSQATPPVPSFPQ------------QSWSREPDSNPGTNE------Daphnia_magna_tr|A0A0P5C974|             -KPERQRSTSLSPALSTSSNPTPTLI--------------SSSSSPSSSQDSGFLPSNQHCaenorhabditis_elegans_tr|W6RRU8|        RSNQTARERQWSERDEISVAPSNTPP-----------------TDPEGNENEFE------Octopus_bimaculoides_tr|A0A0L8FVC4|      SAPSPTSAVHMNDAFATAMNPSLTF---------------------MGINQVSR------Biomphalaria_glabrata_tr|A0A2C9JPB9|     RAGGSITTSQMTTPNSNTVPIISTQ-----------------LQQQQHADLSSTVGA---Mizuhopecten_yessoensis_tr|A0A210Q4R3|   TVSPPQSTSSLGTGSPVSLPPGLTTS--------------THSKAPLGAQLTARLMANPGHelobdella_robusta_tr|T1EF86|            -QVKSYTTANNNTASEAVEPTTTTT---------------------TSDEQPQQ------Homo_sapiens_sp|O95628|                  ----------------------------------------------SPFEGAVTE-SQSLHomo_sapiens_sp|O95628-9|                ----------------------------------------------SPFEGAVTE-SQSLGallus_gallus_tr|Q5ZJC9|                 ----------------------------------------------SPFEGAVTE-SQSLAnolis_carolinensis_tr|G1KKZ3|           ----------------------------------------------SPFEGAITE-SQSLXenopus_laevis_XP_018109568.1            ----------------------------------------------SPFEDAMTE-SQSLDanio_rerio_tr|E7F2Q8|                   ----------------------------------------------SPFEGAAAE-SQSLAcanthaster_planci_XP_022096362.1        ----------------------------------------------SSFEADKSL-PSFLSaccoglossus_kowalevskii_XP_006816490.1  ----------------------------------------------SPFNGLHTDNSSSLDrosophila_melanogaster_tr|M9PCL9|       Q--------------------------------KRLAGTNVQRSVSSCSENSEGHVSESSLimulus_polyphemus_XP_013776233.1        ----------------------------------------------ETLEPSDCKAESSTDaphnia_magna_tr|A0A0P5C974|             EFEILKQGASMAAEEEEIDRARDEADDYFDPAGNKVSG-NSASFAHSILDTNNSSNSHSFCaenorhabditis_elegans_tr|W6RRU8|        ---------------------------------EDIHR-NDVSDLMSKLDVNDDRLARTSOctopus_bimaculoides_tr|A0A0L8FVC4|      ----------------------------------------------QVLEKRNAD-SLSFBiomphalaria_glabrata_tr|A0A2C9JPB9|     ----------------------------------------IGSNRRPGLQEGNSAQGLSFMizuhopecten_yessoensis_tr|A0A210Q4R3|   L--------------------------------------------KPILFDNPAQ-SLSLHelobdella_robusta_tr|T1EF86|            --------------------------------------------------------HGFLHomo_sapiens_sp|O95628|                  FSD--NFRH----------------------------------P-NPIP-----SGLPPFHomo_sapiens_sp|O95628-9|                FSD--NFRH----------------------------------P-NPIP-----SGLPPFGallus_gallus_tr|Q5ZJC9|                 FSD--NFRH----------------------------------P-NPIP-----SGLPPFAnolis_carolinensis_tr|G1KKZ3|           FSD--NFRH----------------------------------P-NPIP-----SGLPPFXenopus_laevis_XP_018109568.1            FSD--NFRH----------------------------------P-NPIP-----SGLPPFDanio_rerio_tr|E7F2Q8|                   FSDNSNFRH----------------------------------P-NPIP-----SGLPPFAcanthaster_planci_XP_022096362.1        AAAN-GYMN-----------------------------------QGVLP-----GLAPPHSaccoglossus_kowalevskii_XP_006816490.1  FSN--TFMP----------------------------------PLRPVP--------PPPDrosophila_melanogaster_tr|M9PCL9|       LSEK-SLTGDYVEEKCNSVNSESQQESVKFQEELEKSNEAIVEAETILPTAESSEDISPALimulus_polyphemus_XP_013776233.1        LQAY----------------------------------------CNIIE-----NQSPPMDaphnia_magna_tr|A0A0P5C974|             FSTG--------------------------------------------P-------LPAYCaenorhabditis_elegans_tr|W6RRU8|        FSEN-DYLG--------------------------------------IP-APAKHQEAPAOctopus_bimaculoides_tr|A0A0L8FVC4|      FSGN-GFSSG------------SERETTKNS--------TIQHS-TGIP-----ESLP--Biomphalaria_glabrata_tr|A0A2C9JPB9|     FEM---------------------------------------------------------Mizuhopecten_yessoensis_tr|A0A210Q4R3|   FSNGNGFHG---------------KRFQKL-------------PVPTVP-----AEVPEPHelobdella_robusta_tr|T1EF86|            LSN---------------------------------------------------------Homo_sapiens_sp|O95628|                  P------------------------SSPQTS-------------------SDWPTAPEPQHomo_sapiens_sp|O95628-9|                P------------------------SSPQTS-------------------SDWPTAPEPQGallus_gallus_tr|Q5ZJC9|                 P------------------------SSPQTS-------------------NDWPTAPEPQAnolis_carolinensis_tr|G1KKZ3|           P------------------------SSPQTS-------------------NDWPMAPEPQXenopus_laevis_XP_018109568.1            P------------------------SSPQTS-------------------SEWPTAPEPQDanio_rerio_tr|E7F2Q8|                   S------------------------NSPQGA-------------------SDWPMTPEPQAcanthaster_planci_XP_022096362.1        PHVQPPVNGSGFQGGSTVPPLIPPRQGPTSA-------------------GDWSEG----Saccoglossus_kowalevskii_XP_006816490.1  V------------------------SVPNANTL--------------PTTNDW------QDrosophila_melanogaster_tr|M9PCL9|       AVAPG--NGE----VEGCLPVVDPVEPPSLADNGSRVTDALSKLNIFDDTPSFFTSPSFQLimulus_polyphemus_XP_013776233.1        C---------------ETPKLDSLQNEPTSALSTYK--------ELDDSEEDWLNHP---Daphnia_magna_tr|A0A0P5C974|             T-AQS--NG----------------TSPSGC---------------------WLANGA--Caenorhabditis_elegans_tr|W6RRU8|        PLMQW--------------EALLGLSSPSAQST---------------------------Octopus_bimaculoides_tr|A0A0L8FVC4|      ------------------------------------------------------------Biomphalaria_glabrata_tr|A0A2C9JPB9|     ---------------------------PNVSLV---------------------------Mizuhopecten_yessoensis_tr|A0A210Q4R3|   V-----------------------------------------------------------Helobdella_robusta_tr|T1EF86|            ------------------------------------------------------------Homo_sapiens_sp|O95628|                  ----------SLFTSETIP-----------VSSSTDWQAAF-------------------Homo_sapiens_sp|O95628-9|                ----------SLFTSETIP-----------VSSSTDWQAAF-------------------Gallus_gallus_tr|Q5ZJC9|                 ----------SLFTSETIP-----------VSSSTDWQAAF-------------------Anolis_carolinensis_tr|G1KKZ3|           ----------SLFTSETIP-----------VSSSTDWQAAF-------------------Xenopus_laevis_XP_018109568.1            ----------SLFTSETIP-----------VSSSTDWQAAF-------------------Danio_rerio_tr|E7F2Q8|                   ----------SLFTSETIP-----------VSSSTDWQAAF-------------------Acanthaster_planci_XP_022096362.1        ----------VILNNDLLP-----------VASHTDWQAAF-------------------Saccoglossus_kowalevskii_XP_006816490.1  ---------DSMFGNDTLP-----------ISSSTNWQEAF-------------------Drosophila_melanogaster_tr|M9PCL9|       QAPILKNKLDLEMRQSHLPDLVNDIDGIQKASNTNEWEEAFKNVMMGNTQHMEEQLLQQQLimulus_polyphemus_XP_013776233.1        ---------DLAFHSESIP-----------VNSSTDWQAAF-------------------Daphnia_magna_tr|A0A0P5C974|             ---------EEEAITSTLP-----------NQSAADWQLAF-------------------Caenorhabditis_elegans_tr|W6RRU8|        ----------IVEPSSLFPTF--------KMDSGFNSQSLF-------------------Octopus_bimaculoides_tr|A0A0L8FVC4|      ----------SLEIPDTIP-----------VTSATDWQAAF-------------------Biomphalaria_glabrata_tr|A0A2C9JPB9|     ----------NHEMADTLP-----------VSTSTDWVEAF-------------------Mizuhopecten_yessoensis_tr|A0A210Q4R3|   ---------STPEIAESIQ-----------VTSCTDWQAAF-------------------Helobdella_robusta_tr|T1EF86|            -----------------VD-----------SSSPTNENANT-------------------Homo_sapiens_sp|O95628|                  ------------------------------------------------------------Homo_sapiens_sp|O95628-9|                ------------------------------------------------------------Gallus_gallus_tr|Q5ZJC9|                 ------------------------------------------------------------Anolis_carolinensis_tr|G1KKZ3|           ------------------------------------------------------------Xenopus_laevis_XP_018109568.1            ------------------------------------------------------------Danio_rerio_tr|E7F2Q8|                   ------------------------------------------------------------Acanthaster_planci_XP_022096362.1        ------------------------------------------------------------Saccoglossus_kowalevskii_XP_006816490.1  ------------------------------------------------------------Drosophila_melanogaster_tr|M9PCL9|       HLQQHQNLRHQLVLQQEEFLRMQELQKRNNFATQINGPANDFLRAYELRAQANAIIQQQLLimulus_polyphemus_XP_013776233.1        ------------------------------------------------------------Daphnia_magna_tr|A0A0P5C974|             ------------------------------------------------------------Caenorhabditis_elegans_tr|W6RRU8|        ------------------------------------------------------------Octopus_bimaculoides_tr|A0A0L8FVC4|      ------------------------------------------------------------Biomphalaria_glabrata_tr|A0A2C9JPB9|     ------------------------------------------------------------Mizuhopecten_yessoensis_tr|A0A210Q4R3|   ------------------------------------------------------------Helobdella_robusta_tr|T1EF86|            ------------------------------------------------------------Homo_sapiens_sp|O95628|                  ------------------------------------------------------------Homo_sapiens_sp|O95628-9|                ------------------------------------------------------------Gallus_gallus_tr|Q5ZJC9|                 ------------------------------------------------------------Anolis_carolinensis_tr|G1KKZ3|           ------------------------------------------------------------Xenopus_laevis_XP_018109568.1            ------------------------------------------------------------Danio_rerio_tr|E7F2Q8|                   ------------------------------------------------------------Acanthaster_planci_XP_022096362.1        ------------------------------------------------------------Saccoglossus_kowalevskii_XP_006816490.1  ------------------------------------------------------------Drosophila_melanogaster_tr|M9PCL9|       LQQHAGENLFGGNMSKFFDFHKSQPQSHHQYLNGHPPQINGNGAVPEPQRVAASLESNRLLimulus_polyphemus_XP_013776233.1        ------------------------------------------------------------Daphnia_magna_tr|A0A0P5C974|             ------------------------------------------------------------Caenorhabditis_elegans_tr|W6RRU8|        ------------------------------------------------------------Octopus_bimaculoides_tr|A0A0L8FVC4|      ------------------------------------------------------------Biomphalaria_glabrata_tr|A0A2C9JPB9|     ------------------------------------------------------------Mizuhopecten_yessoensis_tr|A0A210Q4R3|   ------------------------------------------------------------Helobdella_robusta_tr|T1EF86|            -----------------LD-----------------------------------------Homo_sapiens_sp|O95628|                  -------GFGSS-------------------------------------K-QPEDDLGFDHomo_sapiens_sp|O95628-9|                -------GFGSS-------------------------------------K-QPEDDLGFDGallus_gallus_tr|Q5ZJC9|                 -------GFGSS-------------------------------------K-QQEDDLGFDAnolis_carolinensis_tr|G1KKZ3|           -------GFGSS-------------------------------------K-QQEDDLGFDXenopus_laevis_XP_018109568.1            -------GFGSS-------------------------------------K-QQEDDLGFDDanio_rerio_tr|E7F2Q8|                   -------GFGSS----------------------------------AKQQ-QQDDDLGFDAcanthaster_planci_XP_022096362.1        -------GFSSG-----------------------------------TKDLLNEDDLGFDSaccoglossus_kowalevskii_XP_006816490.1  -------GFTSI------------------------------------KD-KQEDELGFDDrosophila_melanogaster_tr|M9PCL9|       NSPFVENGLINSQQQQQQQQQQQKQRMMGMYEFMPPN-TQSQQNRFTQNS-IVDDDLGFDLimulus_polyphemus_XP_013776233.1        -------GFSPS-----------------------------------NHK-LHDDELGFDDaphnia_magna_tr|A0A0P5C974|             -------NDLASSS-----------------RLVPSV-TQVEDPSSHQQK-NTDDDLGFDCaenorhabditis_elegans_tr|W6RRU8|        -------GTHTSST--------------------PSFRREASPPPGLARF-NSDDDLGFDOctopus_bimaculoides_tr|A0A0L8FVC4|      -------GFAPK-----------------------------------SVD-YQDDDLGFDBiomphalaria_glabrata_tr|A0A2C9JPB9|     -------GLKNK-------------------------------------ELPPEDDLGFDMizuhopecten_yessoensis_tr|A0A210Q4R3|   -------GFGPK-----------------------------------TRD-LPDDDLGFDHelobdella_robusta_tr|T1EF86|            -------GDPNC---------------------------KDDNEDEDSDD-DNEDDLGFDHomo_sapiens_sp|O95628|                  PFDVTRKALADLIEKELSVQDQPSLSPTS-----------LQNSSSHTTTAKGPGSGF-LHomo_sapiens_sp|O95628-9|                PFDVTRKALADLIEKELSVQDQPSLSPTS-----------LQNSSSHTTTAKGPGSGF-LGallus_gallus_tr|Q5ZJC9|                 PFDVTRKALADLIEKELSVQDQPSLSPTS-----------LQNPSPHTTTAKGPGSGF-LAnolis_carolinensis_tr|G1KKZ3|           PFDITRKALADLIEKELSVQDQPSLSPTS-----------LQNPSPHTTTAKGSGSGF-LXenopus_laevis_XP_018109568.1            PFDITRKALADLIEKELSVQDQPSLSPTS-----------VQNPNPH-LAPKGPSSGF-LDanio_rerio_tr|E7F2Q8|                   PFDVTRKALADLIEKELSVQEHSPLSP---------------NPSSHQTG----------Acanthaster_planci_XP_022096362.1        PWNESNKGLADLLEKELQLGIA---------------------------VASDPVKA--TSaccoglossus_kowalevskii_XP_006816490.1  PWDESSKGLAEIMEKELQLNST------------------------ENNRASPEPDR--TDrosophila_melanogaster_tr|M9PCL9|       PFVETQKGLAELMENEVVQKQS--------------------------INNENPLPKLPPLimulus_polyphemus_XP_013776233.1        PWDESAKGLADLLEKEAIQKPE------------------------------QPPEL--IDaphnia_magna_tr|A0A0P5C974|             PFHETQKALAEMLEKESISSLPNGS---------------LFSNGCNNLGYSGPVQSLYSCaenorhabditis_elegans_tr|W6RRU8|        PFTESSKGLSALLQEEQEQIPPHNSASNHTLDVLKQLFGQLPEQRQSTQQHPQHLQS--SOctopus_bimaculoides_tr|A0A0L8FVC4|      PWNESSKALADLMEKENGNRDIRENPNKY-----------LHHHHPHHHHPQQQPQ----Biomphalaria_glabrata_tr|A0A2C9JPB9|     PCAESFKGLADLIEKENGMQQQ-----QH-----------LHLLQSAHSRLGSPPQT--SMizuhopecten_yessoensis_tr|A0A210Q4R3|   PWDESSKGLADLLEKENGISQT------H-----------RPNYTDQNNRSNHQPQ----Helobdella_robusta_tr|T1EF86|            PWDVCSKGLADMMEKEKMLQNLNIFDRTH-----------MPAVNKMNAMNQNALTA--THomo_sapiens_sp|O95628|                  HPAAATNANSLNST-----FSVLPQRFPQFQQHRAVYNSFS---------------FPGQHomo_sapiens_sp|O95628-9|                HPAAATNANSLNST-----FSVLPQRFPQFQQHRAVYNSFS---------------FPGQGallus_gallus_tr|Q5ZJC9|                 HPAAPTNANSLSST-----FPVMPQRFPQFQQHRAVYNSFS---------------FPGQAnolis_carolinensis_tr|G1KKZ3|           HPAAPANANSLNST-----FSVLPQRFPQFQQHRAVYNSFS---------------FPGQXenopus_laevis_XP_018109568.1            HPTPASNANSIAST-----FSVLPQRFPPF-HHRAVYNSFS---------------FPGQDanio_rerio_tr|E7F2Q8|                   -------------------LPNGQQRFPHL-QHRGLYNSFS---------------LPQHAcanthaster_planci_XP_022096362.1        Q-----------------------------------------------------------Saccoglossus_kowalevskii_XP_006816490.1  Q-----------------------------------------------------------Drosophila_melanogaster_tr|M9PCL9|       QPQVPP--------------------------HPQLVDNL------------------QRLimulus_polyphemus_XP_013776233.1        NPRLASVQHSYSHL-----MNAKG-----YPNHLLLNHNQE---------------LPTRDaphnia_magna_tr|A0A0P5C974|             NPPVPPSMA----------------------RHSPSFSSLG--LGLGLGVGVAPQPQPTRCaenorhabditis_elegans_tr|W6RRU8|        HQQHPLQQNQDQHHSFLHQLHAQQQH--QQQQFAADMNRQQDYMYSRLMSQQQQQSQQQROctopus_bimaculoides_tr|A0A0L8FVC4|      HPPTPPQQQPQQQT-----LNIA---------HQAYRNNMR---------------PPIHBiomphalaria_glabrata_tr|A0A2C9JPB9|     YPPHPLSQSPLQNN--------------------------------------------TLMizuhopecten_yessoensis_tr|A0A210Q4R3|   ------------------------------------------------------------Helobdella_robusta_tr|T1EF86|            QA-IPNNYN---------------------HHNASLFNSF-------------------QHomo_sapiens_sp|O95628|                  -AARYPWMAFP-----------------------------------------------RNHomo_sapiens_sp|O95628-9|                -AARYPWMAFP-----------------------------------------------RNGallus_gallus_tr|Q5ZJC9|                 -AARYPWMAFP-----------------------------------------------RNAnolis_carolinensis_tr|G1KKZ3|           -AARYPWMAFP-----------------------------------------------RNXenopus_laevis_XP_018109568.1            -AARYPWVAFP-----------------------------------------------RNDanio_rerio_tr|E7F2Q8|                   MAARHPWMGIP----------------------------------------------TRNAcanthaster_planci_XP_022096362.1        ---PPPGFTIPAN------------------------------------H----------Saccoglossus_kowalevskii_XP_006816490.1  ---PPPGFN-NHVDAF----------------------QSAPTPV-------------PSDrosophila_melanogaster_tr|M9PCL9|       ARMPPPGFNHVNT----------------------------------------------LLimulus_polyphemus_XP_013776233.1        VAPPPPGFGPNHINKL-----------------------------------------VNIDaphnia_magna_tr|A0A0P5C974|             TRIPPPGFNPTQH-------------------------LPGSVNQGGVSHFGLSLPNLSSCaenorhabditis_elegans_tr|W6RRU8|        QFDSTPGFSHPFGSMQ--------------------QQQQSSQQQ---QH----------Octopus_bimaculoides_tr|A0A0L8FVC4|      TPNIPPGFSIGHLQQLQQQIKQQHHQQQQQQQQQQQQQQQQQQQQ---QH-------QQLBiomphalaria_glabrata_tr|A0A2C9JPB9|     LRSLPPGFSISHI-------------------------QQQQLQQ---QH----------Mizuhopecten_yessoensis_tr|A0A210Q4R3|   --SLPPGFSLSQL-------------------------QQQQQQQ---MH----------Helobdella_robusta_tr|T1EF86|            TSTQQPSYA---------------------------QQPHQQQQQ---LHWG---TINGKHomo_sapiens_sp|O95628|                  SIMHLNHTANPTSNSNFLD--------LNLPPQ---------------------------Homo_sapiens_sp|O95628-9|                SIMHLNHTANPTSNSNFLD--------LNLPPQ---------------------------Gallus_gallus_tr|Q5ZJC9|                 SIMHLNHTANPTSNSNFLD--------LNLQPQ---------------------------Anolis_carolinensis_tr|G1KKZ3|           NIMHLNHTANPPSNSNFLD--------LNLPPP---------------------------Xenopus_laevis_XP_018109568.1            NIMHLNHTANPTSNSNFLD--------LSHPPQ---------------------------Danio_rerio_tr|E7F2Q8|                   NLTHLNHTATAAAHSHFLD--------LSMPAQH--------------------------Acanthaster_planci_XP_022096362.1        ---GLGSDAGSSKMLNWLQ--------MDRPADSKPQ--HISSPPSFNNRNPRP------Saccoglossus_kowalevskii_XP_006816490.1  QRPPYRPDLVGSKMMSWMP------HTYTQP-----------------------------Drosophila_melanogaster_tr|M9PCL9|       GLGGASRLQLTSKIIPFMN----------MPVNGVGN-----------------------Limulus_polyphemus_XP_013776233.1        PIPAFSSETSSNKMTSLMN----------MPPNHH----IMNGYNSFQEQVPLPL-----Daphnia_magna_tr|A0A0P5C974|             NRPVNRLDMGTSKMLPFMNNQSTANGGVNGPSTGY-------GPRLYHESPSLSL----GCaenorhabditis_elegans_tr|W6RRU8|        ---QSQSSQSSSLLQDLFNRQQQ-QHQAQQQAQQHQQQQMYAGINSYMYNDMLMPRVPFGOctopus_bimaculoides_tr|A0A0L8FVC4|      HQQMFRQEFNSGKVVEYIYNQ---PKLPQQPVQ-----------NSFHTVLPEM------Biomphalaria_glabrata_tr|A0A2C9JPB9|     ---YFRPDISNSKMPDILP-------QFSLNAQRF-------APLHYHSMPLDL------Mizuhopecten_yessoensis_tr|A0A210Q4R3|   HPAFLRPDIGNNGMLGFIQ--------PNMPP-----------PNSYHSMFDD-------Helobdella_robusta_tr|T1EF86|            SFYQIRHNSSGSKLIDFMS-----------------------SSKSYNNDDSVL------Homo_sapiens_sp|O95628|                  --------------------------------HNTGL----------GGIPVA-------Homo_sapiens_sp|O95628-9|                --------------------------------HNTGL----------GGIPVADNSSSV-Gallus_gallus_tr|Q5ZJC9|                 --------------------------------HSTGL----------GGIPISDNSSSV-Anolis_carolinensis_tr|G1KKZ3|           --------------------------------HGTGL----------GGIPIADNNSSV-Xenopus_laevis_XP_018109568.1            --------------------------------HNTGL----------GGITVADHSNSI-Danio_rerio_tr|E7F2Q8|                   --------------------------------HSTGL----------GGIPISENNGSV-Acanthaster_planci_XP_022096362.1        --------------------------------HH-------------SPSQAQTKHPE--Saccoglossus_kowalevskii_XP_006816490.1  -----------------------------------------------SNSVQHSSEPR--Drosophila_melanogaster_tr|M9PCL9|       -----------------------------------------------SGAQGQHQMPMGVLimulus_polyphemus_XP_013776233.1        ------------------------QGLNNHIKHL-------------PVHSMQSLRPKIGDaphnia_magna_tr|A0A0P5C974|             MSGIGGMSSGGMSSGGMSSNMSYMGGQNGSLSHMSNNKPQGYSTGMGSNMPYSNGSNSLGCaenorhabditis_elegans_tr|W6RRU8|        MAPPPGL-------GGPSTNRS--STTQQAPPHMTQQ----------SQQQQQQQQSSMGOctopus_bimaculoides_tr|A0A0L8FVC4|      --------------------------------HLQ------------GKEQLMQNSSQH-Biomphalaria_glabrata_tr|A0A2C9JPB9|     --------------------------------H----------------TPQHQHQPQR-Mizuhopecten_yessoensis_tr|A0A210Q4R3|   -----------------------------------------------GKEQNHVNAPR--Helobdella_robusta_tr|T1EF86|            ------------------------------------------------------------Homo_sapiens_sp|O95628|                  -----GEEEVKVSTMPLSTSSHSLQQGQQPTSLHTTVA----------------------Homo_sapiens_sp|O95628-9|                --------------------------------------------ESLNMKEWQDGLRALLGallus_gallus_tr|Q5ZJC9|                 --------------------------------------------ESLNMKEWQDGLRALLAnolis_carolinensis_tr|G1KKZ3|           --------------------------------------------ESLNMKEWQDGLRALLXenopus_laevis_XP_018109568.1            --------------------------------------------EGLNMKEWQDGLKALLDanio_rerio_tr|E7F2Q8|                   --------------------------------------------ESINVKEWQDGLRALLAcanthaster_planci_XP_022096362.1        -----------------------------------------------SVKSWQDGLRALLSaccoglossus_kowalevskii_XP_006816490.1  -----------------------------------------------TVKDWQDGLRALLDrosophila_melanogaster_tr|M9PCL9|       ------------------------------------------------------------Limulus_polyphemus_XP_013776233.1        --------------------------------------------NALNMKDWQESLRSMFDaphnia_magna_tr|A0A0P5C974|             L-----------------------------------AASSIGD-TAPSMKDWQDGLRALFCaenorhabditis_elegans_tr|W6RRU8|        LFGMGG------------------------------HQSMMQDHQSQQPQSAQDAFKALLOctopus_bimaculoides_tr|A0A0L8FVC4|      --------------------------------------------DGFHLKDLQDGLRALLBiomphalaria_glabrata_tr|A0A2C9JPB9|     --------------------------------------------ELYNMKDMQEQLRSMLMizuhopecten_yessoensis_tr|A0A210Q4R3|   --------------------------------------------DVFNTKDWQDGLRALLHelobdella_robusta_tr|T1EF86|            ------------------------------------------NSECAVSQDWQDNLKALLHomo_sapiens_sp|O95628|                  ------------------------------------------------------------Homo_sapiens_sp|O95628-9|                P-NININFGGLPNSSSPSNANHSA--------PTSNTATTDS------------------Gallus_gallus_tr|Q5ZJC9|                 P-NINISFGGLPNASSPSNANHSV--------PTSNTATTDS------------------Anolis_carolinensis_tr|G1KKZ3|           P-NININFGGLPNATSPSNANHSV--------PTSNTATTDS------------------Xenopus_laevis_XP_018109568.1            P-NININFGGLSNASTPSNATHSA--------PTSNTATTNS------------------Danio_rerio_tr|E7F2Q8|                   P-NININFGGLPNSTSSSSSSSTSSVNHIG-VPIGSAGISHS------------------Acanthaster_planci_XP_022096362.1        PNNINISFDVANPAGNTARSATHKN-----------------------------------Saccoglossus_kowalevskii_XP_006816490.1  P-NINISFGQQGLSVPPQKDN---------------------------------------Drosophila_melanogaster_tr|M9PCL9|       --NWNAPMGMHQNPGQPVGDSQLQH-------PMAHNKVYNN------------------Limulus_polyphemus_XP_013776233.1        P-NINISFGPPTTQGLPPPNAPPNP-----------------------KVH---------Daphnia_magna_tr|A0A0P5C974|             P-NVNISMGNGGATNSNGNSSNHGG--RIGAFPPGLSGLGNSQQQTMHHQHHQHQLAQQPCaenorhabditis_elegans_tr|W6RRU8|        P-NVNVRF----------------------------------------------------Octopus_bimaculoides_tr|A0A0L8FVC4|      P-NINISFGASPSQPPPPQQQPSSS--------TPCTVQQSQ------------------Biomphalaria_glabrata_tr|A0A2C9JPB9|     P-NINISFGSIPHQQQQQQQAQQHQLQQV---PTSLFSQQSQ--------H---------Mizuhopecten_yessoensis_tr|A0A210Q4R3|   P-NINISFGAA-------QQANNAM--------SAHTSLQQQ------------------Helobdella_robusta_tr|T1EF86|            P-NINISFSPSQTSAAGTLQQQQQQMRRNHVHAGGITIHPAQ------------------Homo_sapiens_sp|O95628|                  ------------------------------------------------------------Homo_sapiens_sp|O95628-9|                -----LSWDSPGS---------W---------------TDPAIITGIPASSGNSL-----Gallus_gallus_tr|Q5ZJC9|                 -----LNWDSSGS---------W---------------MDPAIITGIPASTGNSL-----Anolis_carolinensis_tr|G1KKZ3|           -----LNWDSPGS---------W---------------MDPAIITGIPASAGNSL-----Xenopus_laevis_XP_018109568.1            -----LNWDSSGS---------W---------------MDPAIISGIPSSTGNGL-----Danio_rerio_tr|E7F2Q8|                   -----LSWDSTAS---------W---------------MDPAIITGIPASTGNSL-----Acanthaster_planci_XP_022096362.1        ----SDIWPGMQS-------QTWM-------------VHDPAIISVNSHSTHAPG-FTE-Saccoglossus_kowalevskii_XP_006816490.1  -------WNSASNQ------SNWP-------------TSDPAIITSITVSSAPSTSYTNADrosophila_melanogaster_tr|M9PCL9|       --------------------SDWT-------------SMDPAILSFRQYSSFPQ------Limulus_polyphemus_XP_013776233.1        -----HMWNPYG--------PSWV-------------TQDPAILSSGSIAD---------Daphnia_magna_tr|A0A0P5C974|             NNLATKGWRNNG--------SDWT-------------SLDPAIVSSGQITD---------Caenorhabditis_elegans_tr|W6RRU8|        ------------------------------------------------------------Octopus_bimaculoides_tr|A0A0L8FVC4|      --------------------------------------NNP--------TQKSNF-----Biomphalaria_glabrata_tr|A0A2C9JPB9|     ---LNKSWPLSSSP----DSSPW---------------NDPAIVSTSQLTESGSL-----Mizuhopecten_yessoensis_tr|A0A210Q4R3|   --------------------------------------------------QKSSA-----Helobdella_robusta_tr|T1EF86|            -----NGFNSYHNLLHSQDNTDWLNSGGSMLNTNQSITSDPAIISTGVVTDGMSI-----Homo_sapiens_sp|O95628|                  ------------------------------------------------------------Homo_sapiens_sp|O95628-9|                ----DSLQDDNPPHWLKS----LQALTEMDGP-SAAPSQTH-----------HSAPF---Gallus_gallus_tr|Q5ZJC9|                 ----DTLQDDNPPHWLKS----LQALTEVDGP-SAAPSQTH-----------HSNPF---Anolis_carolinensis_tr|G1KKZ3|           ----DSLQDDNPPHWLKS----LQALTEMDGP-SAAPTQTP-----------HSNPF---Xenopus_laevis_XP_018109568.1            ----ESLQDDNPPHWLKS----LQALTEMDGP-SSSLLPHHP----------HNNLF---Danio_rerio_tr|E7F2Q8|                   ----DCLQDDNPPHWLKS----LQALTEMDGPPSSSALPQPP----------HTGLL---Acanthaster_planci_XP_022096362.1        ----KEVPTEEQPHWLKS----LQTLTDGDTPSHPSAN--------------HLFP----Saccoglossus_kowalevskii_XP_006816490.1  NSDRESNLSESPPHWMKS----LHTLTEVD-----SPT--------------HINNF---Drosophila_melanogaster_tr|M9PCL9|       --------NQIPPHPQQQQDLFLQHLAQQQNSQSGGFN----------NQAQQMLPM---Limulus_polyphemus_XP_013776233.1        ------CRSDSPPHWTKS----LHQLTTEGGT-MNNYN--------------HHMPF---Daphnia_magna_tr|A0A0P5C974|             ------SRSDSPPHWLRS----LEQLTETGNSPAQQPPSNPSNLFGLANSTHYTLPSLTGCaenorhabditis_elegans_tr|W6RRU8|        ------------------------------------------------------------Octopus_bimaculoides_tr|A0A0L8FVC4|      ------SHMPDNPHWMKS----IQQLTEFD---SQGHT--------------HR-PF---Biomphalaria_glabrata_tr|A0A2C9JPB9|     ------FPETSAPHWLKS----LQHLTESDGP-------------------SHRLPF---Mizuhopecten_yessoensis_tr|A0A210Q4R3|   ------LSSDSPPHWLKS----IHQLTDIEGSHTNSQN--------------HRLPF---Helobdella_robusta_tr|T1EF86|            ------------PRWMDQ----YSDLAADASCHIQHHN--------------HLQYRQ--Homo_sapiens_sp|O95628|                  ------------------------------------------------------------Homo_sapiens_sp|O95628-9|                ---------STQIPLHR-ASWNPYPPPS--NPSSFHSPPPGFQTAFRPPSKTPTDLLQSSGallus_gallus_tr|Q5ZJC9|                 ---------GTQIPLHR-ASWNPYSPPS--NPTSFHSPPPGFQTAFRPPSKTPTDLLQSSAnolis_carolinensis_tr|G1KKZ3|           ---------STQIPLHR-ASWNPYSPPS--NPASFHSPPPGFQTAFRPPSKTPTDLLQSSXenopus_laevis_XP_018109568.1            ---------SAQIPLHR-GNWSPYPPPS--NPASFHSPPPGFQTAFRPPTKTPTDLLQSSDanio_rerio_tr|E7F2Q8|                   -------DAAAHLSLHR-AAWAPYLPPPTLTPNQFHSPPPGFQTAFRPQAQTATDILQSAAcanthaster_planci_XP_022096362.1        ---------AHAYANRG-PAWTT------------HAPPPGLRPPTNST--DTHSHTLTESaccoglossus_kowalevskii_XP_006816490.1  ---------YPAHIGRA-GSWFSQ-----------QPPPPGFPTSTAGVHSPTEPQQSL-Drosophila_melanogaster_tr|M9PCL9|       --------GMPNSLLNG-Q----------------QTQPPQVNANVQGMLEFLKSRQFV-Limulus_polyphemus_XP_013776233.1        ---------PMPFAYRA-TQWQGIP----------QSPPPGFSPPFQPSLKSTEPQTLAEDaphnia_magna_tr|A0A0P5C974|             RSNVTPASGLDGLNSMG-SFWPPSV-----SHTTPSMPPPGF-SHIRPTPKTASTAETHKCaenorhabditis_elegans_tr|W6RRU8|        -----------MDDNSM-SRWSHENS----LRSSAVPPPPGFSSVMNR------------Octopus_bimaculoides_tr|A0A0L8FVC4|      ---------VQHFPVRG-TGWVPQT----------HNPPPGFHTQIRPPTQTTEPHKMTEBiomphalaria_glabrata_tr|A0A2C9JPB9|     ---------VQQFPLGGNGGWPPHT----------HNPPPGFRAAGLGSQANTDTHQMAEMizuhopecten_yessoensis_tr|A0A210Q4R3|   ---------VQQFPLRN-TGWPTQT----------QTPPPGFQTSIRPPTHPTEPHKISEHelobdella_robusta_tr|T1EF86|            QNNHYHNYGMHTEPQSS-SSWVQQH--------TSRYPPPGFASSRQHT-EAASEHRLPAHomo_sapiens_sp|O95628|                  -----Homo_sapiens_sp|O95628-9|                TLDRHGallus_gallus_tr|Q5ZJC9|                 ALDRHAnolis_carolinensis_tr|G1KKZ3|           ALDRHXenopus_laevis_XP_018109568.1            ALDRHDanio_rerio_tr|E7F2Q8|                   GIDRHAcanthaster_planci_XP_022096362.1        NPAV-Saccoglossus_kowalevskii_XP_006816490.1  -----Drosophila_melanogaster_tr|M9PCL9|       -----Limulus_polyphemus_XP_013776233.1        HL---Daphnia_magna_tr|A0A0P5C974|             IDNL-Caenorhabditis_elegans_tr|W6RRU8|        -----Octopus_bimaculoides_tr|A0A0L8FVC4|      GLQ--Biomphalaria_glabrata_tr|A0A2C9JPB9|     VLQ--Mizuhopecten_yessoensis_tr|A0A210Q4R3|   GLQ--Helobdella_robusta_tr|T1EF86|            I----
