# Supplementary material for: A conserved CAF40-binding motif in metazoan NOT4 mediates association with the CCR4–NOT complex
Source: Genes Dev. 2019 Feb 1;33(3-4):236–52. doi: 10.1101/gad.320952.118 (PMC6362812; doi:10.1101/gad.320952.118)
Supplement: Supplemental Material [file supp_gad.320952.118_Supplemental_Alignment_File_SF2.zip › Supplemental_Alginment_File_SF2.rtf]

CLUSTAL W (1.7) multiple sequence alignment of plant NOT4Arabidopsis_thaliana_tr|F4JXH7|        ----------MSDHGEKTCPLCAEEMDLTDQQLKPCKCGYQICVWCWHHIVDMAEKDQIEMedicago_truncatula_tr|G7JRI8|         ----------MSDEGERTCPLCAEEMDLTDQQLKPCRCGYEICVWCWHHIMDMAEKDDTDSolanum_tuberosum_tr|M1CY53|           ----------MSDQGDKMCPLCAEEMDLTDQQLKPCKCGYEICVWCWHHIMDMAEKENTECitrus_sinensis_tr|A0A067G3U7|         ----------MSDEGEKTCPLCAEEMDLTDQQLKPCKCGYEICVWCWHHIMDMAEKEETEVitis_vinifera_tr|D7SKR3|              ----------MSDEGEKTCPLCAEEMDLTDQQLKPCKCGYEICVWCWHHIMNMAEKDETEAnanas_comosus_XP_020101472.1          -------MTTMSDDGDRTCPLCAEEMDLTDQQLKPCKCGYDICVWCWHHIMDMAEKEDAEPhoenix_dactylifera_tr|A0A2H3ZM53|     ----------MSDDGERMCPLCAEEMDLTDQQLKPCKCGYEICVWCWHHIMDMAEKEETEAsparagus_officinalis_XP_020267325.1   -------MTTMSDDGDRTCPLCAEEMDLTDQQLKPCKCGYEICVWCWHHIMEMAEKEGSDSorghum_bicolor_tr|A0A1B6PAW2|         -------MTTMSDDGDRTCPLCAEEMDITDQQLKPCKCGYDICVWCWHHIIDMAEKEETEPhalaenopsis_equestris_XP_020579590.1  MAPIPPRMSSVSDDGDTVCPLCAEEMDITDQQLKPCKCGYEICVWCWHHIMEMAEKEKTEArabidopsis_thaliana_tr|F4JXH7|        GRCPACRTPYDKEKIVGMTVNCDSLASEGNMERK-KIQKSKSKSS---------EGRKQQMedicago_truncatula_tr|G7JRI8|         GRCPACRSPYDKEKIVGTAAKCERLLNEMNLEKKVKNQKAKSKSS---------DGRK-QSolanum_tuberosum_tr|M1CY53|           GKCPACRTPYNKEKIVGMEAKCDKVVAEMSTEKRLSSRKGKSKTA---------DSRK-QCitrus_sinensis_tr|A0A067G3U7|         GRCPACRSPYDKEKIVGMAAKCERLVAEISMERKMKSQKSKTKSS---------EGKKQQVitis_vinifera_tr|D7SKR3|              GRCPACRVPYNKEKIVGMAADCKRLVAEINLERKMKSQKAKTKLS---------EGRK-QAnanas_comosus_XP_020101472.1          GRCPACRTPYDKERIV--AANCKRVVAEISAEKKHKSQKVKPKASV--------EARK-HPhoenix_dactylifera_tr|A0A2H3ZM53|     GRCPACRTPYDKERIVGMAANCERVVAEINAEKKQKSQKAKPKASG--------EARK-HAsparagus_officinalis_XP_020267325.1   GRCPACRTTYDKQRIVGMSANCKRMVAEISAEKKQKPQKAKNKSSS------AIEDRK-HSorghum_bicolor_tr|A0A1B6PAW2|         GRCPACRTRYDKDRIVKMAATCDRTVAEKNAEKKHKTQKVKPKAAPPPTAMSTVESKK-HPhalaenopsis_equestris_XP_020579590.1  GRCPACRTPYDKERIVGMSVNCERLVAEMNGGRRHRSQKGKSKVSA--------EARK-HArabidopsis_thaliana_tr|F4JXH7|        LTSVRVIQRNLVYIVGLPLNLADEDLLQHKEYFGQYGKVLKVSMSRTASGVIQQFP-NNTMedicago_truncatula_tr|G7JRI8|         LSSVRVIQRNLVYIVGLPLDLADEDLLQKREYFGQYGKVLKVSMSRTAAGVIQQFP-NETSolanum_tuberosum_tr|M1CY53|           LSSVRVVQRNLVYIVGLPLSLADEDLLQRKEYFSQYGKVMKVSISRTAAGTIQHFA-NDTCitrus_sinensis_tr|A0A067G3U7|         LSSVRVIQRNLVYIVGLPLNLGDEDLLQRREYFGQYGKVLKVSMSRTAAGVIQQFP-NNTVitis_vinifera_tr|D7SKR3|              LGSVRVIQRNLVYIVGLPLNLADEDLLQRKEYFGLYGKVLKVSMSRTAAGVIQQFP-NNTAnanas_comosus_XP_020101472.1          LGGVRVIQRNLVYIIGLPYNLCDESILERREYFGQYGKVLKVSISRPTGAAAQQASLNNTPhoenix_dactylifera_tr|A0A2H3ZM53|     LSSVRVIQRNLVYIIGLPSNLCDESILERREYFGQYGKILKVSISRLTGAAAQQASNNNTAsparagus_officinalis_XP_020267325.1   LSSVRVVQRNLVYIMGMPSNLADESVLERKEYFGQYGKVLKVSVSRQAGTTPQQVASNNTSorghum_bicolor_tr|A0A1B6PAW2|         LASVRVIQRNLVYIIGLPAHLCHESVLERREYFGQYGKVLKVSVSRPTGPPSQASA-NSNPhalaenopsis_equestris_XP_020579590.1  LSTVRVVQRNLVYIIGLPADLCDESLLERKEYFGQYGEILKVSISRSASTTNQQAS-NG-Arabidopsis_thaliana_tr|F4JXH7|        CSVYITYGKEEEAVRCIQAVHGFILDGKPLKACFGTTKYCHAWLRNVACVNPDCLYLHEVMedicago_truncatula_tr|G7JRI8|         CSVYITYSSEEESIRCIQNVHGFILEGRPLRACFGTTKYCHAWLRNAPCINPDCLYLHEVSolanum_tuberosum_tr|M1CY53|           CSVYITYSKEEEAILCIQSVHGFVLDGRPLRACFGTTKYCHAWLRNVPCTNLDCLYLHEVCitrus_sinensis_tr|A0A067G3U7|         CSVYITYSKEEEAVRCIQSVHGFVLEGKSLKACFGTTKYCHAWLRNVPCTNPDCLYLHEVVitis_vinifera_tr|D7SKR3|              CSVYITYSKEEEAVRCIQTVHGFVLDGRPLRACFGTTKYCHQWLRNVPCNNPDCLYLHEIAnanas_comosus_XP_020101472.1          FSVYITYAKEEEAVRCIQAVHNFVLDGKSLRACFGTTKYCHAWLKNMTCSNPDCLYLHDVPhoenix_dactylifera_tr|A0A2H3ZM53|     FSVYITYAREEEAIRCIQAVHNFVLEGKSLRACFGTTKYCHAWLRNMACNNSDCLYLHDIAsparagus_officinalis_XP_020267325.1   FNVYITYAREEEAVRCIQATHNFVLEGKPLRACFGTTKYCYTWLRNMTCSNPDCLYLHDISorghum_bicolor_tr|A0A1B6PAW2|         ISVYITYAKEEEAIRCIQAVHNFVLEGKVLRACFGTTKYCHAWLRNITCGNPDCLYLHDVPhalaenopsis_equestris_XP_020579590.1  -NIYITYAREEDAVRCIQAVHNYVLDGKRLRACFGTTKYCHAWLRNMSCSNPDCLYLHDIArabidopsis_thaliana_tr|F4JXH7|        GSQDDSFTKDEIISAYTRSRVQQITGATNILQHHSGNMLPPPLDAYCSDS--SSAKPIIKMedicago_truncatula_tr|G7JRI8|         GSQEDSFTKDEIISAYT-SRVQQITGVTNSMQRRSGNVLPPPLDDWTNN---STEKPIVKSolanum_tuberosum_tr|M1CY53|           GSQEDSFSKDEIISAYTRSRVQQIAGAINSMQRRSGSVLPPPTEEYCSNNSASEDKPISKCitrus_sinensis_tr|A0A067G3U7|         GSQEDSFTKDEIISAYTRSRVQQITGTTNNLQRRSGNVLPPPFDDYCHINSVSTAKPSVKVitis_vinifera_tr|D7SKR3|              GSQEDSFTKDEIISSYT--RVQQITGATNNLQRRSGNMLPPPADEYCNNSSASMGKPITKAnanas_comosus_XP_020101472.1          GSQEDSFTKDEIISAYTRSRVPQI---ASNLQRRSGNILPPPADDFSSSGT-ASSKPALKPhoenix_dactylifera_tr|A0A2H3ZM53|     GSQEDSFTKDEIISAYTRSRVPQI--ASNNSQRRLGNVLPPPVDDFSSSGT-VTGKQAIKAsparagus_officinalis_XP_020267325.1   GSHEDSFTKDEIISAYTRSRVPQI--PSTILLPRAGNVLPPPIDDLYSNGA-FSTRPSTKSorghum_bicolor_tr|A0A1B6PAW2|         GSQEDSFTKDEIISAYTRTRVPQM--ASSVSQRRTGTVLPPPGDDFSYSAV-VSAKHTFKPhalaenopsis_equestris_XP_020579590.1  GSQEDSFTKDEVISAYTRSVVSQV--PSSNLQRRVGSLL-PPAEDWCNLQT-LSDKHSVNArabidopsis_thaliana_tr|F4JXH7|        VPSTNATSVPR--YSPPSGSGSSSRSTALPAAASWGTH----QSLATSVTSNGSS-----Medicago_truncatula_tr|G7JRI8|         SAPTNSVCAVR--SSPP--NGINGRHVSLPTSAAWGTQTTSCHPPVGGLSHPSVLSKPKPSolanum_tuberosum_tr|M1CY53|           NAATNSAPSVRGSSSPP--NSSSGRSAALPAGALWGTRASNNQHPPASVPCSNGT-----Citrus_sinensis_tr|A0A067G3U7|         NAANNTASISK--DPIP--NGSSARSVALPAAASWGMRASNQQSVATS-ACSNGP-----Vitis_vinifera_tr|D7SKR3|              NASNNSVSIAK--GSPP--NSSSGRSNALPAAASWGMRSSNSQTMASSLSCGNGP-----Ananas_comosus_XP_020101472.1          NGSNIASSQTK--ISPP--NSSAGKST-LPAAASWGNRGLNSKPTAASMSCSQAL-----Phoenix_dactylifera_tr|A0A2H3ZM53|     SVSNNAPSHAK--GSPP--NSSAGKPTILPAAASWGLRSSYCRSPAVSTACSQTP-----Asparagus_officinalis_XP_020267325.1   -PFNSTANQVK--SAPI--DISGTRSTVLPAAASWGLRASNSRSPSSNIASSQNP-----Sorghum_bicolor_tr|A0A1B6PAW2|         NGALNTTNQPR--LSPP--NSSSGRST-LPPAASWGQRDLNARTTATGATSSQSH-----Phalaenopsis_equestris_XP_020579590.1  NASK--ASEVQ--ATVS--NAIAGRLPILPAASFWGSRASKGSMLSATTVCSENS-----Arabidopsis_thaliana_tr|F4JXH7|        -----------DIQRSTSVNGTLPFSAVVAN-----AAHG-PVSSNDILKRPSRKEESQIMedicago_truncatula_tr|G7JRI8|         DTVNSMHPSVLSKPKPDTVNSALAFSTAVTG-----TIQA-SAAQCDGSRRPLLNDESRNSolanum_tuberosum_tr|M1CY53|           -----------LNKKPQTCNPTV-FSTAVES-----LSQV-SLLPAYAGKKVVHTEESVTCitrus_sinensis_tr|A0A067G3U7|         -----------SKQRPDTVGGALAFSSAVAN-----TPSV-STLHVDVVKRPTVHEDSQIVitis_vinifera_tr|D7SKR3|              -----------FKQKPDSFSGSVAFSSAVTSTTLPLTTQA-VALHSEVGKKPTLNEENRLAnanas_comosus_XP_020101472.1          -----------AKPKTETHSSSVLHSSVISS-----TKPVISAWHDDVDTSSKSPESKQVPhoenix_dactylifera_tr|A0A2H3ZM53|     -----------VKQKVEMLNSTSLLSSSTAS-----TKES-SAWHDDVVTTVKIPERRHVAsparagus_officinalis_XP_020267325.1   -----------LSQKIEVLNNSS-SSSMLLS-----TKQN-SAWNDNSVIASKIPEGRHASorghum_bicolor_tr|A0A1B6PAW2|         -----------TKPKSESQSNPFSSSPVISS-----TKTP-SSWNDDTSTATKMSSGQQVPhalaenopsis_equestris_XP_020579590.1  -----------MKQKVETKNHSSTYSSITMG-----AKEVSSAWHDDDVTASLTEEGNGNArabidopsis_thaliana_tr|F4JXH7|        VMDKVKPSVLKPLQHNV----VVSGSERITAPDRDP-TSNRLSSSVDSA-YGGRDID---Medicago_truncatula_tr|G7JRI8|         TIPRVKSEMPKSVKQYISMDSLASASEKTSACDVSP-VPVNLKNELSSR-PLSRDSD---Solanum_tuberosum_tr|M1CY53|           TQERGKIETLEPVKQHVGADPQTYTSENPTIP--APLGSSSMNSQLHSV-PSMSVKD---Citrus_sinensis_tr|A0A067G3U7|         TDSKSKSDISKPSRQHFGSEPPT--------PNGEP-ASVSLSNQASCP---TKYTD---Vitis_vinifera_tr|D7SKR3|              INPKGKLESLESMKQHISMDT----SEGLITPDEAP-ASLPLGGQLSCP-PTSKDND---Ananas_comosus_XP_020101472.1          IQPSNASKPLEPYQPGIQNDYKAKGS-----------AETFLDVDLSSG-PSAWNDDVVVPhoenix_dactylifera_tr|A0A2H3ZM53|     MPMNGISRSSEPSKSGSVKECRTVMS-----------SEGLTDADCASV-PSAWNDDMIVAsparagus_officinalis_XP_020267325.1   VHINGRYSVSEPLKHGSERDHLIKVVDES--------SGTTLDVDHSS--DSAWDDDITTSorghum_bicolor_tr|A0A1B6PAW2|         SEKE--SKTLQPYKPGISKETQALSS-----------LESSLDIDFSTI-PSAWNDDDIVPhalaenopsis_equestris_XP_020579590.1  PLPSGSSK----FHSGQSSLTTES----------DP-LSLSLHFDAHGFGDSAWDDEPIIArabidopsis_thaliana_tr|F4JXH7|        ------QPSAYS------------------------------------------------Medicago_truncatula_tr|G7JRI8|         ------------------------------------------------------------Solanum_tuberosum_tr|M1CY53|           ------------------------------------------------------------Citrus_sinensis_tr|A0A067G3U7|         ------------------------------------------------------------Vitis_vinifera_tr|D7SKR3|              ------------------------------------------------------------Ananas_comosus_XP_020101472.1          TSEGCEGKAAHFEN----------------------------------------------Phoenix_dactylifera_tr|A0A2H3ZM53|     TSKKSE--------P---------------------------------------------Asparagus_officinalis_XP_020267325.1   VKLPDGKHTMHTTGRFRNTITVRCTNAAFVSDTPEVILDEPRHSAWDDDTDLTLNMTEERSorghum_bicolor_tr|A0A1B6PAW2|         VSDGMS------------------------------------------------KGSDENPhalaenopsis_equestris_XP_020579590.1  ESKLVE------------------------------------------------------Arabidopsis_thaliana_tr|F4JXH7|        --------GRDIDK-------------------PSSTVSSFDAAN---------------Medicago_truncatula_tr|G7JRI8|         ---------RGNCT-------------------IANTLNATNITG---------------Solanum_tuberosum_tr|M1CY53|           ---------KDKQM------------------IPTSSTNALDISV---------------Citrus_sinensis_tr|A0A067G3U7|         ---------KSLNM-------------------PPNVIHSSDTTD---------------Vitis_vinifera_tr|D7SKR3|              ---------RGISL-------------------SPKVTNSSDFTR---------------Ananas_comosus_XP_020101472.1          ---------RSLSLES-----LKAVTDEVS--QPSTIIVSPEVDE---------------Phoenix_dactylifera_tr|A0A2H3ZM53|     --------FKSVNVEE-----FRTSGSDLS---PDDVLDAPRTSQKYGSCLSRLLAPAAEAsparagus_officinalis_XP_020267325.1   QIVDNDDRFRSTGTQEPSIAVFRASTTDVTSETFPDVVHTSGVSI---SCSSGQCSPQHESorghum_bicolor_tr|A0A1B6PAW2|         QVANENG-ELTHPASKPL---VLSKKDNITSKSPSDFVSSLAISK---------------Phalaenopsis_equestris_XP_020579590.1  ---------ESFPLQAPS---AVDGECQTSGAGLSTNCASDKISY---------------Arabidopsis_thaliana_tr|F4JXH7|        -----------------------------EAVEDVP------------TVSNLLDGVACMMedicago_truncatula_tr|G7JRI8|         -----------------------------HSFSTGPE---EAVSATNEVIRNLSSEFSSISolanum_tuberosum_tr|M1CY53|           -----------------------------KSSGPGFTK--YSNDTTDVKIQNVCLDMSSLCitrus_sinensis_tr|A0A067G3U7|         -----------------------------HSCLSGPEK--EENVTADVKMQGLCSDVSAMVitis_vinifera_tr|D7SKR3|              -----------------------------QPNCSGSER--EGNVATDGNLHNLLSDMSSMAnanas_comosus_XP_020101472.1          -----RDIPISR-SNSITARSPRSEGLCRKSIGTGRDKITEGHELVDENVQNLCSDLSSVPhoenix_dactylifera_tr|A0A2H3ZM53|     --DKGRGTTLAG-SYTESMNDPIPKGTDGRS--SSADKTAKGSITVNGNIESLCLGLSSVAsparagus_officinalis_XP_020267325.1   NHDTGGTASLPS-SYSENTTYSKDCSY--ELSRSCADKL-SHVSADNGNIESLSLGLSSVSorghum_bicolor_tr|A0A1B6PAW2|         -----SDVSTSDGDHSLTNITPKSLTSNAVDCQSG-EKILEDIGSRNTDMEKLSAQISSVPhalaenopsis_equestris_XP_020579590.1  -----SFSHVSQ-DKDIIFASGKNIDSVNLMCEESLHK--QRCSSTDVNQHTSNMDKSKQArabidopsis_thaliana_tr|F4JXH7|        RITMNCRDERPDITMAIGSQSDQGSIR----QPGSEVSKLPDL-EQCRIDSSINTDKKAIMedicago_truncatula_tr|G7JRI8|         NIDRSTSNELCRITKPSSLPTENALTK----SPQIQEGSHYDV-DRFKDPITTNTAGKTSSolanum_tuberosum_tr|M1CY53|           SIGRHKKSQGNCIDQNKESSP-------------------------------SELTEEYACitrus_sinensis_tr|A0A067G3U7|         SIDRNATNEHSGVARASSALPDHGMMK----LPRNQGLQPYNA-DLCREPLMSPETGKSIVitis_vinifera_tr|D7SKR3|              SIDRQLKSEHPGVLRSNCSLSDNGLTQ----TPGSQGLQQYYA-EQFKESLTSPVSRKVSAnanas_comosus_XP_020101472.1          CLDSSFGANGLKADQPQTLVLNTCSAE----LPLKQTSNLELSFTKQVEQLPSPQASK-GPhoenix_dactylifera_tr|A0A2H3ZM53|     NIDNHLGHGRSNMDKHQTSKSDLSSS-----------PQQCQP-KHEVEHLSSEPPSR-AAsparagus_officinalis_XP_020267325.1   NLDTCPGIDHINAGQQQSSVSDDRFVGKFGHLNSRQHQHQHHP-ENGVESLST------ASorghum_bicolor_tr|A0A1B6PAW2|         KLGGNNDIQSMAGNQQSDVMPCTSVV-----VPMDQNFDKDQS-HLNLDELLLPSENK-DPhalaenopsis_equestris_XP_020579590.1  NMGR---------LRKKNAFAD---------------------YTTLKNQLSRKEQYDSEArabidopsis_thaliana_tr|F4JXH7|        SLEDRIPRTRPGWDWISDLQSQMQGSSKLQVE-DISTLDSQRPHPEEDIIHSRLL--SNLMedicago_truncatula_tr|G7JRI8|         TSVNGVFSPKEQCGGILDSQSQVVSDAAD-IEDDVTSFDNQRLKDPEVCL--SYL--PKASolanum_tuberosum_tr|M1CY53|           TSADEICTTREKSDLRLDAQSKVTQVTTSEMENDLLTFNEQRYRDPEVVIEKVYS--PNLCitrus_sinensis_tr|A0A067G3U7|         TSKNDAFVSREPFDWRTDPTQAATDASPQ-EEEDVLSFDNQRLKDPEVVCRSNYL--PKSVitis_vinifera_tr|D7SKR3|              TTINGVCVPDEQNDWRSDSQTQVVPNMCSEMEDDLLSFDNQRLKDSEVVSGTTYL--PNSAnanas_comosus_XP_020101472.1          YPFYDQFGSSKPFDWSSELPKQGTVVNNN----------NQRLGHSNVITQTSSS--SYQPhoenix_dactylifera_tr|A0A2H3ZM53|     SVLPDACIANELSDWDLDPQKQGLTSPGIEKEDD-------------DNNQPSCS--PYLAsparagus_officinalis_XP_020267325.1   SVFPNVQATGNLSDWRTDQQKQALSSSGNGLVDALAASADQRHGLSELTNLPSCS--PSVSorghum_bicolor_tr|A0A1B6PAW2|         TILSCQYSSDKRLDWSSELQNCSVTPLND-IVDSTVLTDKLHSILLDGSKQPSYSSFAQFPhalaenopsis_equestris_XP_020579590.1  DLGNLASVSTP--SWNIELPKMDLESSGDGDQDSSLAVIDTRQIGSVDATQRSCS--PQFArabidopsis_thaliana_tr|F4JXH7|        SSS---SLDTNHMASRSSLPC---------------EVRGSDRLH------LPNGFG-EKMedicago_truncatula_tr|G7JRI8|         TNFLNISKLSSPCLMQYGEPC---TAGNDGSLSSNDRVR--DESILHSSSMLCNGYP-EKSolanum_tuberosum_tr|M1CY53|           LLSLHSPAQPSGYSSQLING---GGPVRANMQLD----RRTDSVSQPSRESSTNGYP-ENCitrus_sinensis_tr|A0A067G3U7|         ANSLH-------------------------------------VTNHSSSSLKSNGYP-EKVitis_vinifera_tr|D7SKR3|              SHLLHHSNDLRGKSSQHNDIHN-GVSFNADPIFVGRKFSEGSLTHAPGASVISNGFP-EKAnanas_comosus_XP_020101472.1          LHP-SNTANLASYSSWSNDFRSEHSSFTDDSRTGL-------LSSLDNSSILSNSRKGDEPhoenix_dactylifera_tr|A0A2H3ZM53|     HHS-HSSANQCSYSSQNNGVAIKHSTYIGDSKTMVTKADMASFTSRENKSALYNGHK-EDAsparagus_officinalis_XP_020267325.1   PNHLISSSHNCSVGDGLFSV--------GDSRTM--------GRRVNSESLFCNGNK-DQSorghum_bicolor_tr|A0A1B6PAW2|         PST-LDSSLWNDTESNPALMIGTRAS----SQTG--------FSSINNTYVLPNGGQ-DGPhalaenopsis_equestris_XP_020579590.1  THS-LSNHNHQPCDSLNNLDGTINSSS-GESFMH---------TNAVSNYALSNEGD-KVArabidopsis_thaliana_tr|F4JXH7|        SMSSVEH-------------------------SLFANEGRNKVNNAEDAILSNILSLDFDMedicago_truncatula_tr|G7JRI8|         LISGSSN--------GLLRDERNRQSIGRLVGDAVDAGCDAAIDKGESSIISNILSLDFDSolanum_tuberosum_tr|M1CY53|           VSNCVADLHTIDRSYYPLPDEGKRMHVERFQGEAPSENSSTNVDIGESSIISNILSLDFDCitrus_sinensis_tr|A0A067G3U7|         LARNTSGPGRAVENAFLLSNEGQRMPREL-QGD---ANIDAAVDTGENSIISNILSMDFDVitis_vinifera_tr|D7SKR3|              RVGNSAGLDR--------------------------ANASTTMDVGENSIISNILSLDFDAnanas_comosus_XP_020101472.1          QLSSFGNPERVFERPGMKSLEDKANCIGR-YENSSSVEKAASVDKGESSIISDILSLDFDPhoenix_dactylifera_tr|A0A2H3ZM53|     ELSCFSNSGKVFESPEMNCSEERIKYLGK-NDDAATSDKTASVDMGESSIISDILSLDFDAsparagus_officinalis_XP_020267325.1   MSSNFVKIDGANDPDG--SYIGKELGSSD-FGKISNMERSTTVNTEEENIVSKILSLDFDSorghum_bicolor_tr|A0A1B6PAW2|         -LGTVYTHGNVSGHPGIGSLQHRATG----SDSIGSFDKTISVNKDESRIISDMLSSEFNPhalaenopsis_equestris_XP_020579590.1  SLNNMYKPESSLGSAEISSIVG-CRYVDS-SNDLGGSDKDANVNTKESSIITNILSLDFDArabidopsis_thaliana_tr|F4JXH7|        PWDESLTSPHNLAELLGE-VDQRSSTLKPSNFLK-QHNNQSRFSFARYEESSNQAYDSE-Medicago_truncatula_tr|G7JRI8|         PWDDSLTSPHNIVKLLGDNTDSQPCPLKTSSSRNVQSNNQSRFSFARQEESKIQSFDVHPSolanum_tuberosum_tr|M1CY53|           PWNESLTSPQNLAKLLGE-TNDQQGSVRVSSSRK-LTSNQSRFSFAREEPTTNASADYQPCitrus_sinensis_tr|A0A067G3U7|         TWDDPLALPQNLAKLLSE-PEKEPSSLKMSSSWKGHNHNQSRFSFARQEESRSHTFDNERVitis_vinifera_tr|D7SKR3|              AWDDSITSPQNLAQLLGE-NDKQHSSLKTSGSWKVQNSNQSRFSFARQEESKNQVFDIEPAnanas_comosus_XP_020101472.1          PWDDSLSSANNLAKMLGE-SEKAENAFKFSNSWKLQNSNQSRFSFAWQESEGNI---PDPPhoenix_dactylifera_tr|A0A2H3ZM53|     PWDDS-SSANNFSKLLAE-TEKQDRSLKLSSSWRSLNSNQSRFSFARQESQAGG---LEAAsparagus_officinalis_XP_020267325.1   PWDNSWSSANDFAKLLAR-MNKQEGPAKQS-----QSANQSRFSFARQENQANI---ANSSorghum_bicolor_tr|A0A1B6PAW2|         PWDDSYSTANNFVRMLRE-SENNDVNFTAPSWKSGTASKESRFSFARQDNQGNL---LDSPhalaenopsis_equestris_XP_020579590.1  PWNDSLSLTNNLSMLLNE-NDGQNGSFMLSRPRILQNTNQSRFSFARQENQVKS---VEPArabidopsis_thaliana_tr|F4JXH7|        NYSIYGQLSRDKPI-QESAMSRDIYRNNLGSVNGFA-SNFAGGLDNFAASPLFSSHKNP-Medicago_truncatula_tr|G7JRI8|         SYTVSQQQPKSHILNQNLA-ERDFYMEKLGIANGFPTSNFEE----AGVHSIASSNKLSASolanum_tuberosum_tr|M1CY53|           SLNYIEQSFNHYHHGHDFPNSRNDHLDNIGTRNGFSMANNEETVDFGHSFSHLSSNKLS-Citrus_sinensis_tr|A0A067G3U7|         SFSGFIQQPKSHSFNQDFAGNRDPLLDKLGLRNGFHPSSFEESDNFSSNHAVFSPNKLSVVitis_vinifera_tr|D7SKR3|              SFSNIGQVPRNCSFNQNFVESRDPFLDKLGNGSLFSSNIFGESDNFAPGHSVISSNKIS-Ananas_comosus_XP_020101472.1          LIQN--NHEQKLSL------LQNSYGDRY--QGGPVFNASEVPNAATNSSSALTFDRPTGPhoenix_dactylifera_tr|A0A2H3ZM53|     SLRDSGNAQKLCSS------LQDSFGDGS--QHGFQFNNFEGPNAVFNSNLAISSDR-FGAsparagus_officinalis_XP_020267325.1   SYRDMGFAHQQVSS------KQEAYGNCF--GNDYPVSSFGPANSLGNSSSTISSDTNAGSorghum_bicolor_tr|A0A1B6PAW2|         SLRNCGTVTEQNFSLL----PQNSRGNIY--QNGLAFQSLENDFSNGNSLGVLDMAT-AGPhalaenopsis_equestris_XP_020579590.1  SIRDIENMKRGIIL-------KDSLVNDF--QNG-------STAAASSGFCGTLSDRPAGArabidopsis_thaliana_tr|F4JXH7|        VSRPQVSAPPGFSAPNRLPPPGFSSH----------------------------------Medicago_truncatula_tr|G7JRI8|         NSRSQVSAPPGFSIPSRLPPPGFSLH----------------------------------Solanum_tuberosum_tr|M1CY53|           VPRPQMSAPPGFSAPNRAPPPGFTSHF---------------------------------Citrus_sinensis_tr|A0A067G3U7|         AARSQISAPPGFSVPSRAPPPGFTSH----------------------------------Vitis_vinifera_tr|D7SKR3|              ASRAQISAPPGFTVPSRAPPPGFSSH----------------------------------Ananas_comosus_XP_020101472.1          ASRSKISAPPGFSTPNRAPPPGFSSQ----------------------------------Phoenix_dactylifera_tr|A0A2H3ZM53|     VSRAKIAAPPGFSAPSRAPPPGFSSQ----------------------------------Asparagus_officinalis_XP_020267325.1   VTKLKISAPPGFSVPNRAPPPGFSSQ----------------------------------Sorghum_bicolor_tr|A0A1B6PAW2|         TSRSKISAPPGFSAPARVPPPGFSSVFPSQDSLNPTPGFPSGISSHDGSVPLPRFSAFSSPhalaenopsis_equestris_XP_020579590.1  VSKLKMLAPPGFSVPTRSPPPGFSNQ----------------------------------Arabidopsis_thaliana_tr|F4JXH7|        ------------------------------------------------------------Medicago_truncatula_tr|G7JRI8|         ------------------------------------------------------------Solanum_tuberosum_tr|M1CY53|           ------------------------------------------------------------Citrus_sinensis_tr|A0A067G3U7|         ------------------------------------------------------------Vitis_vinifera_tr|D7SKR3|              ------------------------------------------------------------Ananas_comosus_XP_020101472.1          ------------------------------------------------------------Phoenix_dactylifera_tr|A0A2H3ZM53|     ------------------------------------------------------------Asparagus_officinalis_XP_020267325.1   ------------------------------------------------------------Sorghum_bicolor_tr|A0A1B6PAW2|         GISSQEVSKPPTRLPSPFSSGFSSQDGPNTSSRFPSAFSSGLPAQDGPNPPSRFTSAFSSPhalaenopsis_equestris_XP_020579590.1  ------------------------------------------------------------Arabidopsis_thaliana_tr|F4JXH7|        -----ERVGLSSDTTLGNRFLDSTS---LRNAYQVPPPVGNSNGASDIDFVDPAILAVGRMedicago_truncatula_tr|G7JRI8|         -----ERSDQIFDSLSGNSLLDHSSY--LRNSPQTLS-AGNIGGTGEIEFMDPAILAVGKSolanum_tuberosum_tr|M1CY53|           -----ERMEQNFDSFHASHLRDTSS---LHNLHQAPQ-VGHV-SNGDIEFMDPAILAVGKCitrus_sinensis_tr|A0A067G3U7|         -----ERVDQSFDTLSGNHLLDSSSL--LRNTYQMQS-VGNVGSTGDIEFMDPAILAVGKVitis_vinifera_tr|D7SKR3|              -----ERTEQAFDAISGNHLLDTSSL--LRNPYQTPS--GNIASAGDIEFIDPAILAVGKAnanas_comosus_XP_020101472.1          -----DRLNQVYDTPYSENNL-------FGDHYQSHI-AG---NPGDIEFIDPAILAVGKPhoenix_dactylifera_tr|A0A2H3ZM53|     -----DRFNQAYDSTYSDNHLLGSPS--LGNQYQAHL-TG---NPDDVEFIDPAILAVGKAsparagus_officinalis_XP_020267325.1   -----DRYDQTINT-FSESQLIGSP---LQDQFQAHS-TN---NAVDVEFIDPAILAVGKSorghum_bicolor_tr|A0A1B6PAW2|         GFSSQDGSNQSYGSTYQDNLLRDTVLGGNSNHYQSQF--GR--HTSDMEFDDPAILAVGKPhalaenopsis_equestris_XP_020579590.1  -----DRFDQSAMAKVTENYLHGNSL--SRNQYEVRP-TGK--NSVDVEFIDPAILAVGKArabidopsis_thaliana_tr|F4JXH7|        GMV-----NADLDMRS--GFSSQLNSFENETGLHMLRQQSLSSAQQVNGFHHDLRNLSPSMedicago_truncatula_tr|G7JRI8|         GRLQGAQNSQSLDVRS--NFMPQLNYFDNEARLQLLMQRSLAQQQNLR-FSE-IGNTFSQSolanum_tuberosum_tr|M1CY53|           G-FPNGLHLSNLDMSS--SCPPQSNTLQNEGRLQLLMQRSLTAHQNHS-FAD-TRNMFSACitrus_sinensis_tr|A0A067G3U7|         GRLQSGLNNPGLDMRN--NFPSQLNAFENEARLQLMMERSLSPHQNLR-YAN-IGDRLSPVitis_vinifera_tr|D7SKR3|              GRLPGGLNNPALDMRS--NFHPQLSAFENEARLQLLMQRSLSPHQNLR-FAD-IGEGFSPAnanas_comosus_XP_020101472.1          GRMP-GVNDSGLDLKS--GFPAQYSTPNTDPRIQLLMQQSISSHQNLR-LPNHIQDGFLPPhoenix_dactylifera_tr|A0A2H3ZM53|     GRMPLGLNSSGFSSKS--DFPSQFSPSDGDPRLQLLMQQPISSHQNLR-IPDHIGDRFLPAsparagus_officinalis_XP_020267325.1   GRLPLGINNAGIGSRS--SYHQQFTTSESDARIQLLMQQSISASHGPR-IPNHISDRFVPSorghum_bicolor_tr|A0A1B6PAW2|         GLMP-GIGGPELEMKNTPAFQAQLQSASSDPRFQLHVQPNVQSHQNLR-FSDPMQDGLNHPhalaenopsis_equestris_XP_020579590.1  GHMPIGTNINNFG--S--AFPERFRS-EDDLIHQLLTKRS-ASHQNMR-ISNPVMENFFAArabidopsis_thaliana_tr|F4JXH7|        LNDPYGFSSRL-------------------MDQTQGSSLSPFSQLPRQQPSANSILSNGHMedicago_truncatula_tr|G7JRI8|         LGDSYGVSSRL--------------------DQSQVSNLAPYPQLSMQQST-NAILSN-GSolanum_tuberosum_tr|M1CY53|           FGDAYGVSSRGVEQTLANNQYPFDGISSRGLEQTLANHQSPFSQLTLSQSR-NSVIPN-DCitrus_sinensis_tr|A0A067G3U7|         LNDSYGISSRL-------------------MDQPQANNLSPFAQLSIQQSR-NPLISNGGVitis_vinifera_tr|D7SKR3|              LGDAYGIPSRL-------------------MEQSQASNISPFAQLSLQQSR-NAIMSN-GAnanas_comosus_XP_020101472.1          INDNF-VTSRF-------------------LAQNNGA-LSPLELMSLQQPR-NNNFIN-GPhoenix_dactylifera_tr|A0A2H3ZM53|     LNDAY-ITSRL-------------------LAQNHGN-LSPFAQMSLQRPR-SAHISN-GAsparagus_officinalis_XP_020267325.1   HDDAY-TASRF-------------------SAQN--PSLSPFSQISHQQP---SCLLN-NSorghum_bicolor_tr|A0A1B6PAW2|         MNDNY-LASRF-------------------LAQNHGP-VSPYAQIP-QQPR-NSQVTN-GPhalaenopsis_equestris_XP_020579590.1  LNDSY-PTSYL-------------------FPQNHAT-LSPSLQFSLQQPQ-SSPILS-SArabidopsis_thaliana_tr|F4JXH7|        HWDKWNEGQSVNNIGMAELLRNERL-GFNGSLYNNGYEEPKFRIPSPGDVYNRTYGIMedicago_truncatula_tr|G7JRI8|         QWNGWNEVQSGNGLGVAELLRNERL-GFNK--FYPGYDDSKYRMPNSGDIYNRTFGMSolanum_tuberosum_tr|M1CY53|           HWDSWNGVQSGNSLGAAELLRTENL-GFNK--FFTGYEESKIHMPNSGNLYNRTFGMCitrus_sinensis_tr|A0A067G3U7|         HWDGWNEVQGGNSLGMAELLRNERLGGFNK--FYNGYEDSKFRMPSSGDIYNRTFGMVitis_vinifera_tr|D7SKR3|              HWDGWNEIQSGNDLNMAELLRNERL-GYNK--FYTGYEDSKFRMPPSGDLYNRTFGIAnanas_comosus_XP_020101472.1          QWDGWSDIRAGNNMGINEMLRTERF-GLNN--YYSNNEEPKFHIPRSGDLYNRAFGMPhoenix_dactylifera_tr|A0A2H3ZM53|     QWGGWNDVRNGSEMGMPEFLSNERF-GLNN--YYSGNGEHKFHMPSSGDLYNRAFGLAsparagus_officinalis_XP_020267325.1   QWNGWNDMQIGNGIGIPEVLRGERF-GVGN--YFPINEEPKFHLPSSGDIYNRAFGISorghum_bicolor_tr|A0A1B6PAW2|         HWDGWSDSRQGNNTAMSDMSR-----------MLYPSDVNKLHMLGSNDIYNRAFGMPhalaenopsis_equestris_XP_020579590.1  HWDSWNSKQSNTSTGMTDTLKNESF-GLDN--SCIGKAEYRFHIPSSGNIYDRSFGI
